# In-depth, high-accuracy proteomics of sea urchin tooth organic matrix

**DOI:** 10.1186/1477-5956-6-33

**Published:** 2008-12-09

**Authors:** Karlheinz Mann, Albert J Poustka, Matthias Mann

**Affiliations:** 1 Max-Planck-Institut für Biochemie, Abteilung Proteomics und Signaltransduktion, Am Klopferspitz 18, D-82152 Martinsried, Germany; 2 Max-Planck-Institut für Molekulare Genetik, Evolution and Development Group, Ihnestrasse 73, D-14195 Berlin, Germany

## Abstract

**Background:**

The organic matrix contained in biominerals plays an important role in regulating mineralization and in determining biomineral properties. However, most components of biomineral matrices remain unknown at present. In sea urchin tooth, which is an important model for developmental biology and biomineralization, only few matrix components have been identified. The recent publication of the *Strongylocentrotus purpuratus *genome sequence rendered possible not only the identification of genes potentially coding for matrix proteins, but also the direct identification of proteins contained in matrices of skeletal elements by in-depth, high-accuracy proteomic analysis.

**Results:**

We identified 138 proteins in the matrix of tooth powder. Only 56 of these proteins were previously identified in the matrices of test (shell) and spine. Among the novel components was an interesting group of five proteins containing alanine- and proline-rich neutral or basic motifs separated by acidic glycine-rich motifs. In addition, four of the five proteins contained either one or two predicted Kazal protease inhibitor domains. The major components of tooth matrix were however largely identical to the set of spicule matrix proteins and MSP130-related proteins identified in test (shell) and spine matrix. Comparison of the matrices of crushed teeth to intact teeth revealed a marked dilution of known intracrystalline matrix proteins and a concomitant increase in some intracellular proteins.

**Conclusion:**

This report presents the most comprehensive list of sea urchin tooth matrix proteins available at present. The complex mixture of proteins identified may reflect many different aspects of the mineralization process. A comparison between intact tooth matrix, presumably containing odontoblast remnants, and crushed tooth matrix served to differentiate between matrix components and possible contributions of cellular remnants. Because LC-MS/MS-based methods directly measures peptides our results validate many predicted genes and confirm the existence of the corresponding proteins. Knowledge of the components of this model system may stimulate further experiments aiming at the elucidation of structure, function, and interaction of biomineral matrix components.

## Background

The masticatory apparatus of sea urchins (Aristotle's lantern) contains five elongated teeth that have been attractive models for studying biomineralization processes. The constant wearing away of the tips is compensated by continuous tooth growth at the base. The cells responsible for tooth growth arise at the aboral end of the tooth, the plumula, and form multinucleated syncytia, which cover the entire tooth until they are removed by wear at the incisal edge. The syncitial cells form a thin sheet around a vacuole containing the growing tooth into which biomineral precursors are secreted [1-3]. The teeth themselves are complicated structures made of magnesium-enriched calcite crystals [4-7] using amorphous calcium carbonate as precursor [7]. The major building blocks of sea urchin teeth are thin calcite plates assembled at the plumula in vacuoles confined by odontoblast syncytia. The plates are then fused by production of calcareous discs, which enclose the odontoblasts in mineral, leaving them connected to the environment only by narrow, slit-like openings [2]. The mineral phase of teeth also contains a small amount of organic matrix, which is accessible after demineralization [8-11].

Similar to matrices of other biominerals, the organic matrix contained in sea urchin skeletal elements was suggested to play an important role in the mineralization process and in determining biomineral properties [12-14]. However, very few tooth integral matrix proteins have been previously identified at the protein level. Antibodies directed against the spicule matrix (SM) proteins SM30 and SM50, which were first detected as secretion products of embryonal skeletogenic primary mesenchyme cells (PMCs) [15,16], were shown to label the organic matrix of calcification sites confined by odontoblast syncytia [17]. Very recently mortalin, a member of the HSP70 family, was identified in acid-demineralized *Lytechinus variegatus *tooth extracts by Edman sequence analysis of peptides after in-gel digestion of PAGE-separated proteins [11]. However, mortalin was apparently not a constituent of the tooth matrix. It was visualized by antibodies against human mortalin in the interior of odontoblasts and may have to do with syncytium formation rather than tooth mineralization. The recent publication of the *Strongylocentrotus purpuratus *genome [18] renders possible the mass spectrometry-based high-throughput, high-accuracy proteomic analysis of the sea urchin tooth organic matrix. Using such techniques we have identified approximately 138 proteins in the organic matrix of powdered, sodium hypochlorite-washed teeth. Most of these components have not been previously characterized at the protein level and the peptide sequences provided in the present report confirm the existence of many predicted proteins. This is an aspect of proteomic research, which may become ever more important considering the rapidly increasing number of genomes [19,20].

## Methods

### Matrix preparation

Chewing apparatuses of *Strongylocentrotus purpuratus *were washed in 4 × 200 ml of sodium hypochlorite solution (6–14% active chlorine_;_Merck, Darmstadt, Germany) for 60 min at 4–6°C, with changes after 15 min and a 2-min sonication interval (Branson Sonifier model 1200) after every change, followed by extensive washing with de-ionized water. Air-dried lantern elements were collected separately. Teeth were ground to a fine powder with pestle and mortar and the powder was washed again with hypochlorite as above. Complete teeth or tooth powder was demineralized in 50% acetic acid (20 ml/g of dry biomineral) over night at 4–6°C. The turbid suspension was dialyzed successively against 2 × 10 vol. 10% and 2 × 10 vol. 5% acetic acid at 4–6°C (Spectra/Por 6, molecular weight cut-off 1000; Spectrum Europe, Breda, The Netherlands). A white precipitate, which formed during dialysis, and the clear supernatant were lyophilized together.

### Peptide preparation and data acquisition

SDS-PAGE was done with pre-cast 4–12% Novex Bis-Tris gels in the MES buffer system using reagents and protocols supplied by the manufacturer (Invitrogen, Carlsbad, CA). The kit sample buffer was modified by adding SDS and β-mercaptoethanol to a final concentration of 2%, and the sample was suspended in 40 μl sample buffer/200 μg organic matrix, boiled for 5 min, and cooled to room temperature. Gels were loaded with 200 μg of matrix per lane and stained with colloidal Coomassie (Invitrogen) after electrophoresis. Gels were cut into roughly equally sized slices, and slices of three lanes were used for in-gel digestion with trypsin [21] in each of three separate experiments. All slices were treated equally irrespective of staining intensity or presence of visible bands. The eluted peptides were cleaned with C_18 _STAGE-tips before MS analysis [22]. The peptide mixture was separated by nanoscale C_18 _RP-LC (EASY-nLC, Proxeon Biosystems, Odense, Denmark; software version 2.0) coupled on-line to a 7-T LTQ-FT mass spectrometer (Thermo Electron, Bremen, Germany, controlled by Thermo Electron Xcalibur version 2.0 SR2 and LTQ FT Ultra MS 2.2) via a nanoelectrospray ion source for LC-MS. The mass spectrometer operated in a data-dependent mode to automatically switch between MS, MS/MS and MS^3 ^[23,24].

### Data analysis

Raw files (42 for powdered tooth matrix, 39 for intact tooth matrix) were transformed to msm-files using the in-house-made software RAW2MSM, v.1.10 [25]. The single msm-files were used for database searches with the Mascot search engine (Matrix Science, London, UK; version 2.2.04) against a database containing the *Strongylocentrotus purpuratus *annotated gene models (Glean3) protein sequence database ([18]; see also  for further information about Glean [26]), the corresponding reversed database, and the sequences of common contaminants, including human keratins from IPIhuman. Carbamidomethylation was set as fixed modification. Variable modifications were oxidation (M), N-acetyl (protein) and pyro (N-term Q and N-term C). Peptide mass tolerance was set to 5 ppm and the MS/MS tolerance was set to 0.5 Da. Two missed cleavages were allowed. The minimal length required for a peptide was seven amino acids. MS^3 ^scoring, counting of unique and total peptides, and calculation of protein scores was done with MSQuant, v.1.4.2a13 . Each Mascot results file was analyzed separately. The score threshold for peptide acceptance was chosen such as to eliminate any reversed hits at p < 0.05 in each of the Mascot results files. Msm-files containing data of accepted peptides were then merged into one single msm-file for another Mascot search to obtain summed sequence coverage, scores, and peptide numbers. This combined msm-file was also used to search the IPIhuman v.3.46 database for human proteins with high similarity to sea urchin proteins. Identifications with only one unique peptide were accepted only if confirmed by MS^3 ^[23] with a score at least twice the threshold value for acceptance of MS/MS-sequenced peptides and using a MS/MS fragment of at least 5 amino acids, and after manual validation. Quality criteria for manual validation were the assignment of major peaks, the occurrence of uninterrupted y- or b-ion series of at least 3 consecutive amino acids, the preferred cleavages N-terminal to proline bonds and C-terminal to Asp or Glu bonds, and the possible presence of a2/b2 ion pairs.

The abundance of proteins was estimated using the exponentially modified Protein Abundance Index (emPAI) [27] as provided by Mascot (Matrix Science, London, UK; version 2.2.04). BLAST analysis was performed with the program provided by NCBI and searching against the non-redundant database for all organisms. FASTA and MPsrch search programs were used as provided by the European Bioinformatics Institute (EBI; ; ), searching against UniProt Knowledgebase and UniProtKB/Swiss-Prot protein sequence databases. Domains were predicted with NCBI Conserved Domain Search [28] and the MotifScan program , which includes searches against the Prosite and pfam databases.

## Results and Discussion

### Tooth powder matrix

**Figure 1:**
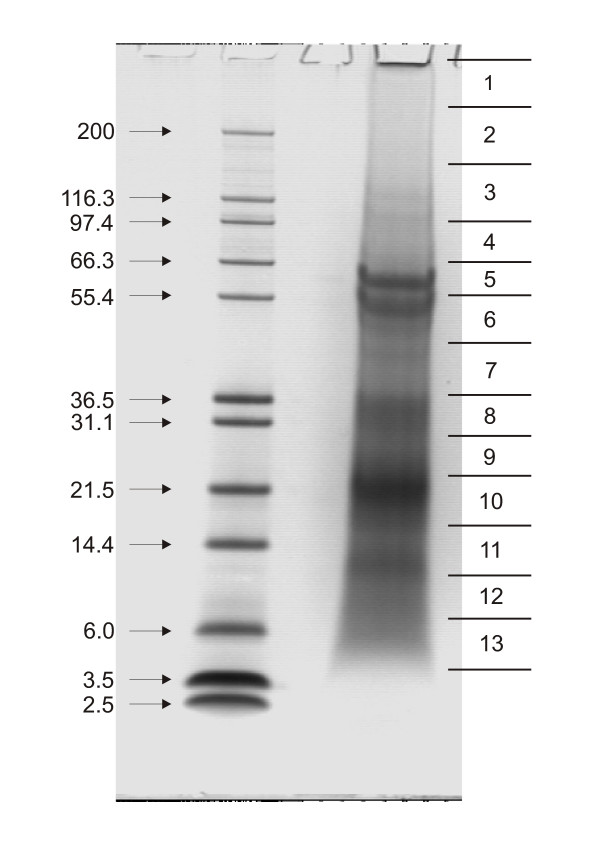
**SDS-PAGE separation of tooth powder organic matrix proteins**. The mass of marker proteins is shown in kDa to the left. Approximately 200 μg of matrix were applied per lane. Sections excised for in-gel digestion are indicated to the right.

Sea urchin teeth contain cavities occupied by odontoblasts, which are connected to the surrounding tissue only by narrow channels [1-5]. Teeth were powdered to destroy their structure and to open as many cavities as possible to render all surfaces accessible to the hypochlorite cleaning solution. Treatment with sodium hypochlorite solution is a widespread method to clean biominerals because it destroys organic material at the surfaces of crystals and effectively removes remnants of adhering tissue. Demineralization of sodium hypochlorite-washed tooth powder from 150 teeth yielded 3.6 μg of organic matrix/mg. The matrix proteins were separated by SDS-PAGE and the Coomassie-stained gels were cut into slices (Figure 1). The protein contained in the slices was digested with trypsin and the eluted peptides were analyzed by ESI-MS.

The matrix of sodium hypochlorite-washed tooth powder yielded approximately 138 identified proteins (Additional file 1: Proteins identified in demineralized tooth powder). The exact number remained unknown because some database entries may have contained sequences of several separate proteins, while the sequences of other proteins may have been erroneously distributed over several different entries. In addition, some peptide sets matched completely or partially to different, but very similar, predicted proteins making it difficult to unequivocally identify the protein isoform(s) possibly involved. Fifty-two proteins were identified only tentatively (Additional file 2: Proteins tentatively identified in the matrix of tooth powder). Tentative identifications were based on unique peptides that frequently were sequenced more than once and yielded good quality spectra with high Mascot scores, but lacked appropriate MS^3 ^confirmation. Fifty-six of the approximately 138 proteins constituting the tooth matrix proteome were identified previously in test (another name for the sea urchin shell) and spine matrix proteomes [29]. A list of the sequences of unique peptides is provided in additional file 3 (Additional file 3: Sequences of unique peptides identified in tooth powder matrix).

A rough estimate of protein abundances was done using the emPAI calculation method, which relates the number of experimentally observed unique ions to the number of peptides obtainable by *in silico *cleavage with a given enzyme [27]. Using this method, we identified spicule matrix protein SM50 as the by far most abundant protein in this matrix, indicating that this is the most abundant protein in all adult skeletal elements [29]. The 10 most abundant proteins also included SM37, SM29, a protein similar to SM29 [Glean3:05991], SM32, and SM30-E [30]. Two other proteins similar to SM29 [Glean3:05989; Glean3:05992] were of lower abundance. Other SM proteins, including SM30-F, which was previously identified as a low abundance protein in spine matrix [29], were not identified unequivocally. Two other proteins of the C-type lectin-like protein family, which were among the five most abundant test matrix proteins [29], were also identified in tooth matrix. However, while the protein contained in Glean3:13825 was also a highly abundant protein in tooth matrix (Additional file 1: Proteins identified in demineralized tooth powder), the protein of Glean3:11163 was of lower abundance and was identified only tentatively (Additional file 2: Proteins tentatively identified in the matrix of tooth powder).

The third most abundant protein after SM50 and SM37 was a hypothetical protein [Glean3:18406] without similarity to any other database entry, and which was previously detected in test and spine matrix with a less prominent abundance ranking position [29]. The peptide set matching this entry also partially matched Glean3:18407. The latter was described previously as a novel, potentially biomineralization-related protein because its expression in the embryo was restricted to PMCs and because its genomic location was adjacent the P16 gene [30], which codes for a protein playing an essential role in regulating spicule growth [31]. Glean3:18406 codes for a protein that lacks a clear signal peptide, contains 30% glycine, and is predicted to have random coil or extended conformation. The function of this quantitatively important protein in tooth matrix remains unknown at present.

Similar to test and spine matrix, the tooth matrix contained MSP130 and the related proteins 1, 2 and 3 (Additional file 1: Proteins identified in demineralized tooth powder). MSP130-related-4, which was part of the test and spine matrix [29], was not identified. Tooth matrix also nearly contains the complete set of metalloproteases identified in test and spine matrix previously [29]. The only exception was Sp-MT-MMP-a, which was identified in spine matrix, but not in test or tooth. Inhibitors of metalloproteases were previously shown to interfere with larval skeleton formation [32-34]. The known functions of metalloproteases in other animals may indicate a role for these enzymes in matrix protein maturation. Such maturation processes were shown recently to occur during and after secretion of the larval matrix protein SM30-B [35], a protein which was, however, not identified in adult skeletal elements.

Other proteins of known relevance to biomineralization are the carbonic anhydrases, which provide carbonate ions for mineral formation. The tooth matrix contained among the most abundant proteins the same carbonic anhydrase as test and spine matrix (Additional file 1: Proteins identified in demineralized tooth powder). A less abundant hypothetical protein [Glean3:25722] was predicted to contain a carbonic anhydrase domain and may be another member of this enzyme family. This entry did not contain the signal sequence of secreted proteins. However, the N-terminus of the carbonic anhydrase domain was also lacking, indicating that this entry did not contain the complete sequence.

Similar to test and spine matrix, tooth powder matrix also contained some proteins known to be intracellular. This group included ubiquitin and actin, two proteins widespread in tissues, body fluids, and frequently observed in proteomic studies. These proteins have amino acid sequences that are highly conserved in animals, rendering it difficult to unequivocally determine their origin [29]. Using the IPIhuman database as a reference, proteins sharing peptides with mammalian proteins were identified and flagged appropriately in protein and peptide tables of additional files.

### Comparison to the matrix of intact teeth

To study the contribution of odontoblast cells reported to be embedded in the mineralized tooth structure [2,4], or of residual cellular material protected in odontoblast cavities against destruction by hypochlorite, to the analysis results, we analyzed the matrix of intact, but sodium hypochlorite-treated teeth. The main reason for this experiment was to clarify, if possible, the origin of intracellular proteins identified in tooth powder matrix, and our expectation was that contaminants from extra-crystalline tissue would increase in abundance. Demineralization of 40 bleached teeth yielded ~2.5 μg of organic matrix/mg. At least 144 proteins were identified in this matrix (Additional file 4: Proteins identified in demineralized intact teeth). In addition, 37 proteins were identified tentatively. Tentative identifications were based on MS/MS spectra of single unique peptides without MS^3 ^confirmation, but with manual validation of the spectra (Additional file 5: Proteins tentatively identified in the matrix of intact teeth). A list of the sequences of unique peptides is provided in additional file 6 (Additional file 6: Sequences of unique peptides identified in intact tooth matrix). One hundred and ten of these 144 proteins were also identified in crushed tooth matrix. The most abundant protein in intact tooth matrix was SM50. However, its relative abundance, as determined by emPAI calculation, was drastically decreased in comparison to its abundance in matrix of crushed teeth (Additional file 4: Proteins identified in demineralized intact teeth). This was also observed for most of the other spicule matrix proteins, for carbonic anhydrase [Glean3:12518], for most metalloproteases, and for MSP130-related proteins 2 and 3. It has to be kept in mind however, that emPAI is only a rough indication of relative abundances and is not a substitute for exact protein quantitation, which is not possible at present for this experimental system. Nevertheless, there were some very distinct trends. Thus, many intracellular proteins appeared only in intact tooth matrix, showed increased emPAI in intact tooth matrix, or were identified only tentatively in powdered tooth matrix. This was especially conspicuous in the case of histones, which appeared in great number and high abundance in intact tooth matrix, with histone H4 even being the second most abundant protein (Additional file 4: Proteins identified in demineralized intact teeth), and a few other proteins such as tubulin, nuclear intermediate filament protein, elongation factor 1α, or β-catenin. However, given that intact cells contain thousands of different proteins, the identified differences were small. Almost all of the proteins that were new or increased in abundance were structural proteins of cytoskeletal elements or chromatin. This may indicate that hypochlorite treatment combined with sonication left no intact cells in tooth cavities but only remnants of particularly resistant structures tenaciously adhering to crystal surfaces. However, there were also some intracellular proteins, which decreased less dramatically, or not at all, in powdered tooth matrix. Examples were ubiquitin, endoplasmatic reticulum calcistorin, or cyclophilins. These intracellular proteins may have been present in the mineralization space as by-products of secretion of specific matrix proteins or may have been released by damaged, leaky syncytial cells, and were subsequently incorporated into the growing matrix.

In the following sections we will discuss some selected proteins, which were not identified previously in sea urchin skeletal elements, and which appeared to be of some interest for various reasons.

### Proteins with Ala- and Pro-rich and acidic Gly-rich motifs

**Figure 2:**
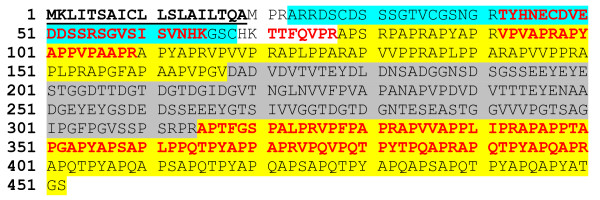
**Analysis of the AP-rich protein contained in entry Glean3:17589**. The predicted signal peptide is in bold and underlined. The predicted Kazal domain is shaded blue. Yellow and grey shading indicate Ala/Pro-rich motifs and acidic sequence regions, respectively. Peptides identified by MS/MS are in red.

The tooth matrix proteome included a group of five moderately acidic, hypothetical, proline-, alanine- and glycine-rich proteins, which were not identified in any sea urchin skeletal element previously. The proteins had theoretical global pI values of 4 to 5 and a global content of 17–21% Pro and 14–25% Ala. All of these proteins contained a predicted signal sequence indicating that they were secreted proteins. Their relative abundance did not vary much between powdered tooth matrix and intact matrix, suggesting a dual distribution. Because of the signal sequences typical for secreted proteins, the extra-crystalline fraction of this protein group may have resided in the extracellular matrix deposited by odontoblasts and lining the mineralization compartment [1,3]. The major amino acids were not distributed evenly along the sequence but arranged in more or less distinct blocks of AP-rich, neutral to basic sequences and acidic, often Gly-rich sequences. Furthermore, four of these five proteins contained either one or two predicted Kazal protease inhibitor domains. The most abundant protein of this group was encoded in entry Glean3:17589 (Figure 2). A predicted signal peptide was followed by a predicted domain of ~40 amino acids with a sequence similarity to Kazal-type serine protease inhibitor domains. The next stretch of amino acids (~aa78-167) contained 35% proline, 22% alanine, 13% valine, and 13% arginine, but was devoid of any negatively charged amino acid. By contrast, aa168-314 consisted of 13% aspartic acid, 10% glutamic acid, and 16% glycine. This acidic motif (calculated pI~3.1) was followed by another AP-rich sequence containing 35% proline, 22% alanine, and 10% glutamine. The tooth proteome contained four other, less abundant, proteins consisting of the same general elements, but with some variations. In entry Glean3:17590 (Additional file 7: Proteins with Ala and Pro-rich and acidic Gly-rich motifs) a predicted signal peptide of 38 amino acids was followed by a strongly matching acidic pfam_ls:Kazal_1 domain (aa39-88). The ensuing sequence consisted of alternating acidic Gly-rich motifs and Ala/Pro-rich motifs not containing any acidic amino acid. The six acidic motifs were of variable length and frequently contained a high percentage of Ser, and sometimes Tyr, in addition to acidic residues and Gly. This entry was identified with a set of peptides partially matching a similar protein contained in Glean3:22278 (Additional file 7: Proteins with Ala and Pro-rich and acidic Gly-rich motifs). However, the Glean3:22278 sequence contained an additional set of Kazal domain and alternating AP-rich and acidic domains. The C-terminal acidic domain of Glean3:22278 contained 15 DEED, or very similar, repeats, which were often preceded by lysine (Additional file 7: Proteins with Ala and Pro-rich and acidic Gly-rich motifs). Entry Glean3:17587 contained a protein that was almost as abundant as that encoded by Glean3:17590, but was the only one of this group lacking a predicted Kazal domain (Additional file 7: Proteins with Ala and Pro-rich and acidic Gly-rich motifs). The signal peptide was followed by a cysteine-containing acidic stretch of sequence that was in turn followed by two alternating AP-rich and Gly-rich acidic domains. Finally, Glean3:17588, similar to Glean3:22278, coded for a protein containing a duplicated set of features. The Glean3:17588 protein (Additional file 7: Proteins with Ala and Pro-rich and acidic Gly-rich motifs) contained a predicted signal peptide joined by an acidic Val-rich motif (~31% valine, 20% Glu, and ~16% alanine) to alternating AP-rich and acidic G-rich motifs interrupted twice by a predicted Kazal domain. The second Kazal domain, located approximately in the middle of the predicted sequence, was separated from a block of alternating basic AP-rich and acidic Gly-rich motifs by a short sequence of amino acids containing three APGGGGGQIPR repeats (Additional file 7: Proteins with Ala and Pro-rich and acidic Gly-rich motifs).

Several SM proteins contain Pro-rich domains. But the short repeats constituting these domains have a completely different composition due to a high frequency of Gln and Asn [30]. SM30 also contains in its sequence Gly- and Ala-rich motifs, but these are much less extended and there is no regular pattern of alternating Ala/Pro-rich and Gly-rich acidic sequence blocks as described for the novel tooth matrix proteins above. Furthermore, the proteins described in this section did not contain the mandatory C-type lectin-like domain characteristic for SM proteins. Various programs predicted a random coil conformation for the tooth proteins described in this section. This is a feature in common with the unrelated, intrinsically disordered, otolith matrix protein starmaker [36], an acidic protein that controls vertebrate otolith crystal formation and is part of the otolith matrix [37]. A random coil, disordered, extended, and flexible structure was also determined for the PGMG repeat motif of spicule matrix protein PM27 [38] and the PNNP repeat motif of SM50 [39]. This kind of structure may be a general feature of invertebrate biomineral matrix proteins containing simple amino acid repeats.

### Hypothetical protein with similarity to selenoprotein

**Figure 3:**
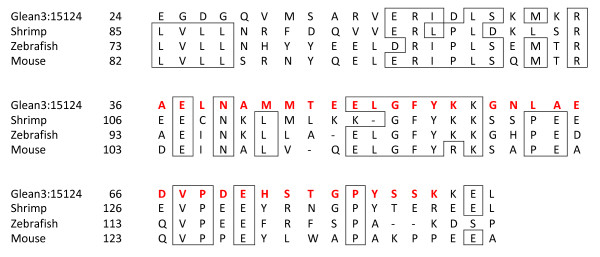
**Alignment of Glean3:15124 to selected selenoprotein M sequences**. Amino acids conserved in three of the four sequences are in boxes. The numbering of sequence positions corresponds to the numbering in precursor proteins. The sequences are from UniProtKB/TrEMBL entries A5J2A1_PENVA (*Litopenaeus vannamei*), SELM_DANRE (Zebrafish, primary accession number Q802G7), and SELM_MOUSE (primary accession number Q8VHC3). Peptides sequenced by MS/MS are shown in red.

The most abundant novel protein that occurred in tooth powder matrix, but not in intact tooth, test or spine matrix, was a small protein encoded in entry Glean3:15124 (Additional file 1: Proteins identified in demineralized tooth powder). A predicted signal sequence stretching from aa1-23 was followed by a sequence with high similarity to sequences of invertebrate and vertebrate selenoprotein M (Figure 3). Considering the matching part of the sequence only and starting at position 34 of Glean3:15124, sea urchin and shrimp sequences were 49% identical, and sequence identity to selected vertebrate selenoproteins was 44–47%. The peptides were eluted from section 11 of the gel, roughly corresponding to a mass of 12–15 kDa (Figure 1). This was twice the mass calculated for the presumed mature protein encoded in Glean3:15124. However, the apparent molecular mass, derived from relative mobility in PAGE, matched the mass of complete selenoproteins (~14 kDa). Thus, the sum of evidence indicated that Glean3:15124 contained only the C-terminal part of a selenoprotein M, erroneously joined to another piece of sequence containing a signal peptide sequence, while the N-terminal part, which would have included the selenocysteine, was contained in another entry. The distribution of partial sequences of a protein over several Glean entries was not exceptional. Another example of a protein sequence distributed over several Glean3 entries encountered in the present and the previous [29] study was the protein similar to ECM3 of *L. variegatus *([Glean3:23016, 01796 and 18054]; Additional file 1: Proteins identified in demineralized tooth powder).

### Protein P19

The predicted sea urchin protein P19 [Glean3:04136] was not identified in any skeletal element of the sea urchin previously, but the coding message of this protein was highly abundant in, and specific for, PMCs [40]. The predicted mass of this protein (~19 kDa) did not agree with its migration in gels where it was found in sections 1–3 (M_*r *_> 100,000) indicating that it formed aggregates via its predicted oligomerization motif [40], was bound to other proteins despite the denaturing electrophoresis buffer, or was part of a bigger protein. This protein was identified exclusively in the matrix of intact teeth (Additional file 4: Proteins identified in demineralized intact teeth). Therefore, if this protein has a function in biomineralization, as suggested previously [40], this function apparently does not depend on localization in the mineralization compartment.

### HSP70 and mortalin

Mortalin, a member of the HSP70 family, was identified in guanidine and HCl extracts of *Lytechinus variegatus *teeth [11]. Using antibodies against a vertebrate mortalin/GRP75, the protein was localized immunohistochemically in the syncytium-forming odontoblasts. However, the occurrence of acidic SSD and SD motifs in C-terminus of sea urchin mortalin was taken as a hint as to a possible direct interaction with calcium carbonate [11]. We identified several entries "similar to HSP70" in intact tooth matrix (Additional file 4: Proteins identified in demineralized intact teeth) and also tentatively in powdered tooth matrix (Additional file 2: Proteins tentatively identified in the matrix of tooth powder) but none of the proteins detected corresponded to the suggested *S. purpuratus *mortalin [11] contained in Glean3:22158 of the database. Thus mortalin was apparently not contained in the proteome of hypochlorite-cleaned *S. purpuratus *tooth matrix.

### Miscellaneous proteins

Proteins not identified previously in sea urchin skeletal elements included cyclophilin-1 (Additional file 1: Proteins identified in demineralized tooth powder), which was previously shown to be expressed exclusively in skeletogenic mesenchyme cells in the embryo and was suggested to play a role during mineral deposition [41]. Our results indicated that cyclophilin-1 may perform the same function(s) in tooth mineralization. Another peptidyl-prolyl cis/trans isomerase identified at the same abundance level (Additional file 1: Proteins identified in demineralized tooth powder) was Sp-FK506-binding protein 2 [Glean3:18964]. A protein similar to peptidyl-prolyl cis/trans isomerase B was also contained in Glean3:13756. An obvious role for these enzymes, which were identified at the same abundance level in both, powdered tooth matrix and intact tooth matrix, would be to assist the correct folding of matrix proteins along the secretion pathway.

The powdered tooth matrix also contained several proteins containing predicted galactosyltransferase [Glean3:10644, Glean3:20773], glycosyltransferase [Glean3:23855], and sulfotransferase domains [Glean3:10032, Glean3:15125] and a predicted α-mannosidase II [Glean3:21559]. Most of these proteins occurred at low abundance. Glean3:10644 and Glean3:23855 proteins were detected in powdered tooth and intact tooth matrices, the others were identified in powdered tooth matrix only (Additional file 1: Proteins identified in demineralized tooth powder). These enzymes are typical Golgi apparatus residents and may have reached the biomineralization space as by-products of secretion processes and may eventually have been incorporated into the growing tooth elements.

## Conclusion

Previous studies have shown that sea urchin and mammalian tooth proteins contain immunologically cross-reactive components, indicating the presence of very similar epitopes, if not proteins [9,10]. This may eventually turn out to be correct at the epitope level, but proteome analysis did not yield conclusive similarities between sea urchin and vertebrate tooth matrix in terms of protein homologies. Instead, the most abundant proteins of the tooth matrix were almost the same as those of test and spine matrix. An almost identical set of matrix spicule proteins was identified in the matrices of all three adult skeletal elements, with SM50 being the by far most abundant component. Metalloproteases, which have been shown to be essential for larval spicule mineralization [32-34] and may be important for the maturation of matrix proteins [35], were also present at high abundance and number, as were proteins of the MSP130 family and carbonic anhydrases. This suggested an important role for these proteins in sea urchin biomineralization processes in general. The ranking according to relative abundance (emPAI) indicated that many of these proteins may occur at different concentrations in different skeletal compartments. However, because emPAI is not a measure of absolute protein concentration and depends on too many variables, such as possible protein modifications or experimental conditions, we have used it only as a rough guide to discern major from minor proteins.

The tooth matrix also contained many intracellular proteins that were already identified in the previous analysis of test and spine matrix [29]. To evaluate the possible contribution of odontoblast material that resisted hypochlorite treatment because of its location in mineral-enclosed cavities, we also analyzed teeth that were washed with hypochlorite but not crushed before hypochlorite treatment. The results showed an increase in the abundance and number of intracellular proteins such as histones and cytoskeletal proteins, while SM proteins and other presumed crystal-occluded proteins mostly decreased in abundance indicating that their proportion to total protein was diminished. Other intracellular proteins, such as ubiquitin, calcistorin or peptidy-prolyl cis/trans isomerases, remained essentially unchanged. Several enzymes involved in carbohydrate synthesis and trimming, such as α-mannosidase, glycosyl- and galactosyltransferases or sulfotransferases, either remained essentially unchanged or appeared only in powdered tooth matrix, probably due to the depletion of bulk intracellular protein. Many of these proteins are Golgi apparatus residents and may have reached the mineralization space as by-products of secretion processes and may have become incorporated into the crystals as mineralization proceeded. Many of the histones, cytoskeletal proteins, and other intracellular proteins were also detected in matrices of hypochlorite-treated test plates and spines [29], indicating in retrospect a contribution of cellular remnants surviving in stereom cavities. However, the actual size of this contribution remains unclear at present, because some of these proteins remained unchanged or even increased in abundance and number in the matrix of crushed teeth. Furthermore, some proteins that are commonly known as intracellular proteins may also occur extracellularly and may have functions in the extracellular space. For instance, secreted histones and histone fragments were shown to have antimicrobial activity and were suggested to be part of an ancient innate immune system (reviewed in [42]).

The most interesting novel proteins in tooth matrix were a group of previously uncharacterized proteins that share the presence of acidic Gly-rich domains separated by neutral or basic Ala- and Pro-rich motifs, and the presence of short sequences similar to Kazal-type protease inhibitor domain sequences. These proteins occurred in both types of preparation at approximately the same abundance, indicating their presence in intra- and extra-mineral compartments. All of these proteins contained in their sequence a predicted signal peptide typical for secreted proteins. This may indicate that the extra-mineral fraction may have resided in the matrix lining the mineralization space [1] and may imply a role of these proteins at the mineral-matrix interface.

Similar to recent proteomic studies of biomineral matrices [29,43], the results of sea urchin tooth matrix analysis showed a previously unrecognized complexity of the proteome. The sea urchin tooth proteome analyzed in the present report represents an average of different tooth regions such as tip, shaft and plumula, and of different structures within these regions, such as the shaft plates and stone parts [1,4], and different proteins may be localized specifically in such specialized compartments. However, proteomic inventories like the one presented in this report will be instrumental in designing new experiments aimed at the elucidation of localization, structure, function, and interplay of biomineral matrix components.

## Abbreviations

Aa: amino acid; emPAI: exponentially modified Protein Abundance Index; PMC: primary mesenchyme cell; SM: spicule matrix.

## Competing interests

The authors declare that they have no competing interests.

## Authors' contributions

KM conceived the study, performed organic matrix and peptide isolation and data acquisition. AJP provided the animals. KM and AJP did database searches and annotations. MM supplied methodological expertise. All authors took part in the design of the study and were critically involved in data interpretation and manuscript drafting. All authors read and approved the final manuscript.

## Supplementary Material

Additional file 1 Proteins identified in demineralized tooth powder. List of proteins identified in the organic matrix of demineralized hypochlorite-treated tooth powder. Click here for file

Additional file 2 Proteins tentatively identified in the matrix of tooth powder. Identifications with a single unique peptide showing good quality, manually validated, spectra without MS^3 ^confirmation. Click here for file

Additional file 3 Sequences of unique peptides identified in tooth powder matrix. List of sequences of accepted peptides from tooth powder matrix. Click here for file

Additional file 4 Proteins identified in demineralized intact teeth. List of proteins identified in the organic matrix of demineralized hypochlorite-treated intact teeth. Click here for file

Additional file 5 Proteins tentatively identified in the matrix of intact teeth. Identifications with a single unique peptide showing good quality, manually validated, spectra without MS^3 ^confirmation. Click here for file

Additional file 6 Sequences of unique peptides identified in intact tooth matrix. List of sequences of accepted peptides from intact tooth matrix. Click here for file

Additional file 7 Proteins with Ala- and Pro-rich and acidic Gly-rich motifs. Identification of motifs in proteins with Ala and Pro-rich and acidic Gly-rich sequences contained in entries Glean3:17590, Glean3:17587 and Glean3:17588. Click here for file
