# Supplementary material for: In-depth, high-accuracy proteomics of sea urchin tooth organic matrix
Source: Proteome Sci. 2008 Dec 9;6:33. doi: 10.1186/1477-5956-6-33 (PMC2614417; doi:10.1186/1477-5956-6-33)
Supplement: Additional file 1 — Proteins identified in demineralized tooth powder. List of proteins identified in the organic matrix of demineralized hypochlorite-treated tooth powder. [file 1477-5956-6-33-S1.doc]

Proteins identified in demineralized tooth powder.

|  |  |  |  |  |  |  |  |  |  |  |
| --- | --- | --- | --- | --- | --- | --- | --- | --- | --- | --- |
| **GLEAN3**  **accession** | **SwissProt/**  **Trembl**  **accession** | **Protein** |  | **Protein scores** | **Unique pep-tides** | **Total**  **accep-ted**  **pep-tides** | **Sequ-ence**  **cover-age** | **Gel**  **section** | **emPAI** |  |
|  |  |  |  |  |  |  |  |  |  |  |
| 18811 | P11994 | Spicule matrix protein SM50 | **T,S** | 1622 | 16 | 2228 | 67% | 3-9 | 7687.3 | I, ↑ |
| 18813 | O76450 | Spicule matrix protein SM37 | **T,S** | 1032 | 11 | 578 | 47% | 3-8,11,12 | 10.0 | I, ↑ |
| 18406  18407 |  | Hypothetical protein; Gly-rich; gene close to P16 gene, PMC-specific expression | **T,S** | 1153 | 15 | 242 | 72% | 7-11 | 9.9 | I, ↑ |
| 21385  16506 | Q8MUK7 | MSP130-related-2; entries cover different but overlapping regions | **T,S** | 3022 | 32 | 787 | 64% | 1-11 | 9.1 | I, ↑ |
| 05990 | Q8MUL0 | Spicule matrix protein SM29 | **T,S** | 630 | 7 | 394 | 34% | 7-11,13 | 7.7 | I, ↑ |
| 05991 |  | Similar to spicule matrix protein SM29 | **S** | 588 | 8 | 99 | 50% | 7,9-11 | 7.4 | I, ↑ |
| 13825 |  | Hypothetical protein; domain: CLECT | **T,S** | 764 | 7 | 242 | 21% | 5-11,13 | 4.7 | I, ↑ |
| 13821  02088 | P08472 | (Similar to) MSP130 | **S,T** | 2167 | 20 | 650 | 31% | 3-5,7-10 | 4.0 | I |
| 18810 | Q8MUL1 | Spicule matrix protein SM32 | **T,S** | 406 | 5 | 347 | 15% | 1,3-9,11,12 | 3.8 | I, ↑ |
| 04867 |  | Similar to spicule matrix protein SM30/SM30-E | **T,S** | 665 | 7 | 490 | 20% | 3-9 | 3.5 | I, ↑ |
| 06387  13823 |  | Similar to MSP130/MSP130-related-3 | **T,S** | 2551 | 30 | 575 | 34% | 4-8,11,12 | 2.8 | I, ↑ |
| 13670 |  | Similar to matrix metalloproteinase 14/Sp-MT-MMP-e | **T,S** | 1523 | 15 | 361 | 28% | 6-13 | 2.4 | I, ↑ |
| 12518 |  | Similar to carbonic anhydrase | **T,S** | 873 | 9 | 239 | 20% | 6-12 | 2.2 | I, ↑ |
| 28749 |  | Similar to vertebrate metalloproteinase MMP-24/Sp-MT-MMP-b | **T,S** | 1259 | 13 | 270 | 25% | 5-9,12,13 | 1.8 | I, ↑ |
| 15124 |  | Hypothetical protein; possibly partial selenoprotein |  | 136 | 2 | 3 | 41% | 11 | 1.7 |  |
| 28887 |  | Similar to cytoplasmic cystatin |  | 274 | 4 | 8 | 47% | 11,12 | 1.7 | (I), ↑ |
| 03536 |  | Similar to Os09g0542200; domain: DSBA (thioredoxin) |  | 649 | 6 | 24 | 29% | 9,10 | 1.4 | I, ↑ |
| 17589 |  | Hypothetical protein; domains: Kazal, AP-, G- and DE-rich motifs |  | 860 | 10 | 64 | 29% | 1-4 | 1.3 | I |
| 07484 | Q45UE8 | Cyclophilin 1 |  | 523 | 4 | 18 | 27% | 9,11 | 1.2 | I |
| 23052 |  | Hypothetical protein LOC753888 | **T,S** | 377 | 4 | 14 | 30% | 9-11 | 1.2 | I |
| 18964 |  | Similar to MGC53657 protein/Sp-FK506 binding protein 2; domain: FKBP_C |  | 435 | 4 | 14 | 37% | 10,11 | 1.2 | I |
| 27172 |  | Hypothetical protein LOC585657; domain: partial esterase_lipase superfamily (MhpC) |  | 638 | 8 | 13 | 36% | 8,9 | 1.1 | I |
| 03918 |  | Similar to vitellogenin receptor | **S** | 806 | 7 | 27 | 24% | 2-4 | 1.1 | I, ↑ |
| 26000 |  | Hypothetical protein; domain: IG | **T,S** | 1028 | 12 | 57 | 12% | 6-9,11 | 1.1 | I |
| 17590 |  | Hypothetical protein; peptide set partially overlapping with entry Glean3_22278; domain: Kazal, AP- and G-rich motifs |  | 785 | 6 | 329 | 14% | 1-5 | 1.0 | I |
| *04721* |  | *Similar to ubiquitin/ribosomal protein S27a; all peptides in ubiquitin domain (aa1-~76)* | ***T,S*** | *250* | *3* | *38* | *21%* | *1-9,13* | *1.0* | *I* |
| 04746 |  | Similar to *P. lividus* FGF receptor 2 | **T** | 684 | 8 | 42 | 22% | 6-9 | 1.0 | I |
| 13669 |  | Similar to metalloproteinase/Sp-MT-MMP-d | **T,S** | 779 | 8 | 94 | 19% | 6-9,11,12 | 1.0 | I, ↑ |
| *09481* | *P53472* | *Actin; peptide set matches to many actin-encoding entries* | ***S*** | *618* | *8* | *12* | *27%* | *6* | *1.0* | *I* |
| 25722 |  | Hypothetical protein/Sp-carbonic anhydrase-12-like-B; domain: α-CA (carbonic anhydrase) |  | 235 | 3 | 6 | 21% | 7,11 | 0.9 | I |
| 17587 |  | Hypothetical protein; AP- and G-rich motifs |  | 514 | 6 | 114 | 12% | 1-5 | 0.9 | I |
| 05989 |  | Similar to spicule matrix protein SM29 | **T,S** | 228 | 3 | 22 | 23% | 9-11 | 0.9 | I |
| 15848 |  | Hypothetical protein LOC764009 |  | 221 | 3 | 7 | 28% | 8,9 | 0.8 | I |
| 09606 |  | Similar to MGC86229 protein; domain: FReD (fibrinogen-related) |  | 431 | 6 | 11 | 25% | 7,8 | 0.8 |  |
| 22598 |  | Similar to angiopoietin-like protein, partial/Sp-Fred/Sp-fmo5-like; domain: FReD |  | 476 | 6 | 19 | 19% | 7,8 | 0.8 |  |
| 00204  26040 |  | Similar to thrombospondin, type I, domain containing 4 |  | 230 | 3 | 6 | 34% | 4,6-9 | 0.8 |  |
| 13756 |  | Similar to peptidyl prolyl isomerase B; domain: cyclophilin_ABH_like |  | 367 | 4 | 9 | 21% | 9 | 0.7 | I |
| 10169 |  | Hypothetical protein; domains: 2 IG | **T** | 410 | 5 | 41 | 17% | 5-9,11 | 0.7 | I, ↑ |
| 23016  01796  18054 |  | Similar to extracellular matrix protein 3 (ECM3) of *L.variegatus;* N-terminus  Similar to ECM3*;* C-terminus  Similar to ECM3*;* joining N- and C-terminus | **S**  **S** | 2015  1732  346 | 26  20  5 | 200  203  7 | 19%  17%  15% | 1-4,7,8  2-5,7  2,3 | 0.7  0.6  0.5 | I  I  I, ↑ |
| 10644 |  | Hypothetical protein/Sp-B3galt1; domain: Galactosyl_Transferase |  | 593 | 7 | 25 | 23% | 4,7-11 | 0.7 | I |
| 07930 |  | Hypothetical protein/Sp-Dnase-gamma; domain: Exo_endo_phos |  | 422 | 5 | 8 | 25% | 8 | 0.7 | I, ↑ |
| 09549 |  | Similar to Sdcbp-prov protein; domains 2 PDZ_signaling; N-term: acetyl-S2 | **S** | 371 | 5 | 6 | 15% | 7 | 0.6 |  |
| 21260 |  | Hypothetical protein; domains: 3 LDLR | **T,S** | 408 | 5 | 22 | 26% | 4,6,7 | 0.6 | I |
| 06812 |  | Hypothetical protein LOC579471; domains: IG, 2 IGcam | **T** | 920 | 10 | 89 | 11% | 6-12 | 0.6 | I |
| 25235 |  | Similar to Egfl6-prov protein; domains: CCP/SUSHI, EGF_Ca, 2 partial vWA_matrilin | **T,S** | 1204 | 13 | 43 | 19% | 4-11 | 0.6 | I, ↑ |
| 25966 |  | Hypothetical protein/Sp-LRR15-like; domains: IG, FN3, LRR | **T,S** | 1346 | 15 | 124 | 15% | 3-9,11,12 | 0.6 | I |
| 11332 |  | Similar to Ca^2+^-activated chloride channel | **T,S** | 1289 | 14 | 76 | 18% | 4-12 | 0.5 | I |
| 14602 |  | Similar to scavenger receptor cysteine-rich protein  type 12/Sp-SRCR-115; domains: 4 complete 1 partial SRCR |  | 505 | 6 | 24 | 14% | 2-4 | 0.5 |  |
| 22278 |  | Hypothetical protein; peptide set overlapping with entry Glean3_17590; AP- and G-rich motifs |  | 326 | 4 | 27 | 8% | 1-5 | 0.5 | I |
| 05538 |  | Hypothetical protein LOC576239/Sp-LRR/Ig receptor; domains: IG | **T,S** | 580 | 7 | 60 | 12% | 5-12 | 0.5 | I |
| 27906 |  | Hypothetical protein LOC577685/SpC-lectin-PMC1; domain: partial CLECT | **T,S** | 419 | 5 | 89 | 14% | 1,3-9,11,12 | 0.5 | I, ↑ |
| 06103 |  | Hypothetical protein LOC756971/similar to calsyntenin-1 |  | 178 | 2 | 13 | 8% | 3,4 | 0.5 | I |
| 00439 |  | Hypothetical protein LOC575608 | **T** | 462 | 6 | 19 | 14% | 5-7,11 | 0.5 | I |
| 17586 |  | Similar to hepatopancreas kazal-type proteinase inhibitor; domains: 6 Kazal |  | 288 | 3 | 32 | 3% | 2-5 | 0.5 | I, ↑ |
| 17588 |  | Hypothetical protein LOC575627; domains: 2 Kazal, AP- and G-rich motifs, V-rich motif |  | 1315 | 16 | 164 | 11% | 1-5,9,11 | 0.5 | I |
| 13822 | Q8MUK8 | MSP130-related-1 | **T,S** | 467 | 5 | 34 | 12% | 3,4,8 | 0.4 | I |
| 20612 |  | Hypothetical protein; domains: 2 tentative EGF-like domains |  | 373 | 5 | 27 | 14% | 2-4,7 | 0.4 | I, ↑ |
| 12486 |  | Similar to MEGF (multiple EGF) 11 protein; domain: EMI |  | 231 | 2 | 5 | 8% | 9 | 0.4 | I, ↑ |
| 11562 |  | Similar to phospholipase A2; domain: PLA2c |  | 272 | 2 | 3 | 19% | 10,11 | 0.40 | I |
| 27236 |  | Similar to voltage-dependent anion channel 2 isoform 1; domain: Porin_3; N-term: N-acetylated Ala2 | **S** | 272 | 3 | 3 | 12% | 7 | 0.4 | I |
| 05228 |  | Hypothetical protein LOC586019/Sp-calsyntenin-like; domains: CA (cadherin), LamG |  | 316 | 4 | 9 | 13% | 3,4,7 | 0.4 | I, ↑ |
| 00469 |  | Similar to cell adhesion molecule OCAM; domains: 5 IG | **T** | 471 | 6 | 10 | 13% | 3,11 | 0.4 | I, ↑ |
| 07341  04145 |  | Similar to neurexin IV/Sp-CASPR_C-term; domains: 2 LamG; shares peptide with Glean3_18348 |  | 397 | 4 | 9 | 11% | 2,3 | 0.3 |  |
| 26042 |  | Similar to thrombospondin type I domain containing protein 4/similar to CG6232-PA; domains: TSP_1, PLAC (protease and lacunin) |  | 280 | 4 | 14 | 11% | 5-7,9 | 0.3 | I |
| 14421 |  | Hypothetical protein |  | 161 | 2 | 3 | 12% | 7,8,11 | 0.3 | I |
| 04105 |  | Similar to T cell-specific protein | **S** | 127 | 2 | 2 | 9% | 7 | 0.3 |  |
| 18348 |  | Similar to contactin-associated protein 5, partial/ similar to neurexin IV; domains: 1 FA58C, 2 LamG; stretch of Asp at N-term; pI~4.4; shares peptide with Glean3_07341 |  | 625 | 8 | 30 | 11% | 2-4 | 0.3 | I, ↑ |
| 00438 |  | Similar to peptidylaminoacyl-L/D-isomerase | **T** | 477 | 6 | 18 | 14% | 5,7,9,10 | 0.3 | I |
| 05385 |  | Similar to membrane-type matrix metalloproteinase 1 alpha/Sp-MMP-f | **T** | 319 | 4 | 6 | 8% | 6,7,9,12 | 0.3 | I, ↑ |
| 04584 |  | Similar to MGC80358 protein, domains: 2 MIR , 1 partial MIR |  | 192 | 2 | 5 | 14% | 8,9 | 0.3 | (I), ↑ |
| 08354  27667 |  | Hypothetical protein; domains: IG, partial IG |  | 494 | 5 | 49 | 7% | 5-9,11,13 | 0.3 | I |
| 09601 |  | Similar to cathepsin Z precursor/Sp-Cts4; domain Peptidase_C1 |  | 207 | 2 | 4 | 10% | 7 | 0.3 |  |
| 03612  19655 |  | Hypothetical protein LOC752450/Sp-astacin 1; domain: ZnMc_astacin_like; overlapping peptide sets | **S** | 362 | 4 | 14 | 8% | 7-9,11 | 0.3 | I |
| 20457 |  | Hypothetical protein LOC762504; domains: 2 IG | **T** | 345 | 4 | 33 | 10% | 4,6-9 | 0.2 | I, ↑ |
| 11180 |  | Hypothetical protein; domains: FN3, EGF_CA | **T,S** | 429 | 5 | 17 | 8% | 5-7 | 0.2 | I |
| 00475  25962 |  | Similar to MGC139263 protein; domain: annexin |  | 108 | 1 | 3 | 8% | 4,7 | 0.2 | I, ↓ |
| 05420 |  | Similar to scavenger receptor cysteine-rich protein type 12/Sp-SRCR-42; domains: 5 SRCR, 1 CCP |  | 358 | 4 | 17 | 7% | 2-4 | 0.2 | I |
| 08863 |  | Hypothetical protein LOC575414/Sp-timp3; domain: partial NTR_TIMP |  | 319 | 4 | 9 | 8% | 9,10 | 0.2 | I, ↑ |
| 26949 |  | Similar to Solute carrier family 34 (sodium phosphate), member 2, partial (aa38-698)  Similar to melanotransferrin/EOS47 (aa699-1419);  peptides in transferring domains | **T,S** | 806 | 9 | 13 | 7% | 4,5 | 0.2 | I, ↑ |
| 23330 |  | Hypothetical protein LOC592585; domain: rDP_like (dipeptidase) |  | 241 | 3 | 5 | 7% | 5 | 0.2 |  |
| 05238 |  | hypothetical protein LOC589368; domain:PSI superfamily | **T** | 309 | 3 | 10 | 9% | 4-7 | 0.2 |  |
| 09352 |  | Hypothetical LOC585121 protein |  | 83 | 1 | 6 | 7% | 10-13 | 0.2 | (I) |
| 08613  13077 |  | Similar to TFP250; possibly fragments of one protein |  | 271  411 | 3  5 | 12  17 | 6%  3% | 2-4,9 | 0.2  0.1 | I  I |
| 18919 |  | Similar to RPGR; domain: partial MDN1; very acidic (pI~3.9); contains many short repeats of the type EXSSGEEQPK |  | 251 | 3 | 31 | 7% | 1-5 | 0.2 | I |
| 05992 |  | Similar to SM29 | **T** | 177 | 3 | 6 | 4% | 4,8 | 0.2 | I, ↑ |
| 10589 |  | Hypothetical protein LOC585716; Gly-rich, pro-rich and Asp-rich motifs; pI~4 |  | 154 | 1 | 11 | 4% | 1-3,5-7 | 0.2 | I |
| 01892 |  | Similar to IP13724p; domain: partial sema | **T** | 235 | 3 | 5 | 6% | 3,4 | 0.2 | I, ↑ |
| 11138 |  | Hypothetical protein; domains: partial SRCR, WSC |  | 162 | 3 | 10 | 8% | 6,7 | 0.2 | I |
| 10032 |  | Hypothetical LOC579709 protein/Sp-C6ST (chondroitin sulfotransferase); domain: partial Sulfotransf_1 |  | 210 | 2 | 3 | 5% | 9,13 | 0.2 |  |
| 08505 |  | Hypothetical protein/Sp-Cys-rich-FGFR; domains: 8 Cys-rich FGFR |  | 327 | 3 | 6 | 5% | 4,7,8 | 0.2 | I |
| 23855 |  | Similar to MGC81998 protein; domain: Glycosyl_transferase_8 |  | 182 | 2 | 6 | 5% | 6,7,9 | 0.2 | I, ↑ |
| 15906 |  | Hypothetical protein LOC587327 |  | 518 | 8 | 18 | 4% | 7,8 | 0.2 | I, ↑ |
| 27169 |  | Similar to fibulin-6 | **T,S** | 197 | 2 | 2 | 6% | 6 | 0.2 | I, ↑ |
| 04850 |  | Similar to Synaptotagmin IX; domains:  partial CLCA_N, vWFA |  | 443 | 5 | 15 | 5% | 4-9 | 0.2 | I, ↑ |
| 26072 |  | Similar to neprilysin/Sp-endothelin-converting enzyme; domains: 2 peptidase_M13 |  | 273 | 4 | 4 | 5% | 3 | 0.2 |  |
| 11293 |  | Similar to HrES-AP; domain: alkPPc (alkaline phosphatase) |  | 260 | 3 | 4 | 5% | 5 | 0.2 | (I), ↑ |
| 21559 |  | Similar to α-mannosidase II isozyme; domains: partial Glyco_hydro_38, α-mannosidase_middle |  | 234 | 3 | 3 | 5% | 3 | 0.2 |  |
| 20773 |  | Hypothetical protein/Sp-B3galt5; domain: Galactosyl_transferase |  | 173 | 2 | 2 | 5% | 7 | 0.2 |  |
| 28030 |  | Similar to furin1-X; domains: partial ABC_membrane, peptidase_S8 (subtilisin family; P_preprotein, FU (furin) |  | 110 | 2 | 4 | 2% | 4 | 0.2 |  |
| 18702 |  | Hypothetical protein LOC587099/Sp-Txndc4; domains: 3 PDI/ERp44 (thioredoxin superfamily) |  | 196 | 2 | 4 | 5% | 6,7 | 0.1 | I |
| 23289 |  | Similar to thrombospondin, type I, domain containing 4 |  | 128 | 2 | 4 | 6% | 7 | 0.1 |  |
| 03540 |  | Similar to prominin |  | 273 | 3 | 7 | 3% | 1-3 | 0.1 | (I) |
| 16731 |  | Hypothetical protein LOC575507; domain: partial glycerophosphodiesterase |  | 160 | 2 | 5 | 4% | 2-4 | 0.1 |  |
| 04412 |  | Hypothetical protein/Sp-CUB/EGF; domains: 1 partial and 1 complete CUB, 7 partial vWA_M (matrilin_like) |  | 283 | 4 | 5 | 5% | 2,3,8 | 0.1 |  |
| 16052 |  | Similar to apolipophorin/Sp-vitellogenin 2 |  | 1229 | 15 | 31 | 3% | 4,6,7,9,11,  12 | 0.1 | I |
| 25068 |  | Similar to tetraspannin | **T** | 147 | 1 | 15 | 4% | 1-5,7,9 | 0.1 | I, ↓ |
| *14869*  *06211* |  | *Similar to thioredoxin peroxidase* | ***T*** | *81* | *1* | *1* | *3%* | *9* | *0.1* | *I, ↓* |
| 07682 |  | Hypothetical protein LOC757239/Sp-CPE(carboxypeptidase E); domain: peptidase_M14 | **T,S** | 181 | 2 | 5 | 7% | 6-8 | 0.1 | I |
| 28748 | Q4G2F5 | Matrix metalloproteinase 16/Sp-MT-MMP-h | **T,S** | 158 | 2 | 6 | 3% | 5-7 | 0.1 | I |
| 16497 |  | Hypothetical protein; domain: SCP, HX |  | 238 | 3 | 3 | 3% | 4 | 0.1 |  |
| 15125 |  | Hypothetical protein LOC577607; domain: Gal-3-O_sulfotransferase |  | 120 | 2 | 4 | 3% | 4,6,7,12 | 0.1 |  |
| 15321 |  | Hypothetical protein; domain: partial 7tm_2 (secretin family) |  | 239 | 3 | 3 | 4% | 7 | 0.1 |  |
| 22672 |  | Hypothetical LOC581872 protein; domain: partial alkppc (alkaline phosphatase) |  | 84 | 1 | 1 | 2% | 6 | 0.1 |  |
| 15404 |  | Similar to DEAH (Asp-Glu-Ala-His) box polypeptide 33/Sp-LamG/EGF fragment 3; domains: 3 complete and 1 partial LamG, 1 EGF |  | 274 | 4 | 10 | 3% | 2-4 | 0.1 | I, ↑ |
| 22057 |  | Similar to MGC68835 protein/Sp-SemaA; domains: Sema, PSI, 3 TSP_1 |  | 259 | 3 | 16 | 3% | 2-7,12 | 0.1 | I |
| 23115 |  | Similar to brain RPTPmam4 isoform II/Sp-PTPRiz (receptor tyrosine phosphatase; domains: 12 FN3, 2 PTPc (protein tyrosine phosphatase), 1 EGF_Ca | **T,S** | 610 | 7 | 16 | 4% | 3,4,7-9,11 | 0.1 | I |
| 27046 |  | Similar to GA21473-PA, partial; domains: 3 IG |  | 111 | 1 | 2 | 1% | 6 | 0.1 |  |
| 18452 |  | Flagellasialin/Ps-sema6; domain: partial sema |  | 212 | 2 | 4 | 3% | 6,9,10 | 0.1 | (I) |
| 21428 |  | Similar to Nr-CAM protein/Sp-L1 |  | 288 | 3 | 4 | 5% | 6,7 | 0.1 |  |
| 12695  17753 |  | Similar to Usp16/Sp-ATP6ap1 (Vacuolar ATP synthase subunit 1); domain: partial AZP_synt_S1  Or: Similar to metalloproteinase; domains: ZnMc_MMP, HX, PG_binding_1 |  | 113 | 1 | 2 | 4% | 9 | 0.1 |  |
| 26094 |  | Similar to cathepsin 1; domains: partial pancreatic_lipase_like, peptidase_C1A; partial overlap with entries Glean3_10929 and 00416 (similar to lipase H) |  | 171 | 2 | 11 | 3% | 6,7 | 0.1 | I |
| 28091  25310 |  | Similar to echinonectin | **T,S** | 176 | 2 | 3 | 2% | 7,10,12 | 0.1 | I |
| 25502 |  | Hypothetical protein/Sp-neogenin/DCC-like adhesion receptor; domains: 7 IGcam, 4 FN3 |  | 334 | 4 | 10 | 2% | 2,3 | 0.1 | I |
| 24565 |  | Similar to thioester-containing protein; domains: A2M_N, A2M_N_2, A2M, A2M_2, A2M_receptor; | **T,S** | 164 | 2 | 8 | 2% | 2-5 | 0.1 | I |
| 00983 |  | Similar to ADAMTS-like 3, domains: 2 TSP_1 |  | 107 | 1 | 2 | 3% | 4 | 0.1 |  |
| 25772 | Q27780 | ERcalcistorin/PDI |  | 85 | 1 | 1 | 2% | 7 | 0.1 | I, ↓ |
| 00453 |  | Similar to hemicentin, partial; domains 4 IG, 6 IGcam |  | 222 | 3 | 8 | 2% | 3,4,6,7 | 0.1 |  |
| 18768 |  | Hypothetical protein LOC589972/similar to X-prolyl aminopeptidase; domains: APP, partial creatinase |  | 160 | 2 | 3 | 2% | 4 | 0.1 |  |
| 16016 |  | Hypothetical protein/Sp-Notch ligand 5;domains: 9 EGF_Ca |  | 127 | 2 | 3 | 2% | 2 | 0.1 | I |
| 26146 |  | Hypothetical protein; domain: PLA2c |  | 100 | 1 | 3 | 2% | 9,11 | 0.1 | I |
| *05296* |  | *ATP synthase β-subunit/similar to H^+^-transporting ATPase β-subunit* |  | *107* | *1* | *1* | *2%* | *1* | *0.1* |  |
| 27885 |  | Similar to ferroxidase; domains: 2 partial Cu-oxidase_3 |  | 118 | 2 | 2 | 1% | 4 | 0.1 |  |
| 19695 |  | Hypothetical protein/Sp-IGF2R, domains: 10 complete and 2 partial CIMR (Cation-independent mannose-6-phosphate receptor repeat) |  | 165 | 2 | 3 | 1% | 2,3 | <0.1 |  |
| 24019  19665 |  | Similar to nephrin; domains: 7 IG, 1 FN3 |  | 155 | 2 | 3 | 1% | 3 | <0.1 | I |
| 20031 |  | Similar to Peptidyl-glycine alpha-amidating monooxygenase-B |  | 80 | 1 | 2 | 1% | 7 | <0.1 |  |
| 19034 |  | Hypothetical protein; domains: 3 IG-like |  | 115 | 1 | 2 | 1% | 4,5 | <0.1 |  |
| 11588 |  | Similar to 2 alpha fibrillar collagen (no triple helix!); domains: Kazal, Zip (zinc transporter) | **S** | 215 | 2 | 4 | <1% | 7,8 | <0.1 | (I) |

Proteins are ordered according to decreasing emPAI. The average absolute mass accuracy was 0.8 ppm (p<0.05). Mascot protein scores were calculated with MSQuant from unique peptide scores including MS^3^ scores. If the protein was identified in more than three gel sections only sections containing more than 5% of the total peptide number are indicated. S, also identified in spines; T, also identified in test [29]. Proteins sharing peptides with human entries are shown in *italics*. I, proteins also identified in intact tooth matrix; (I), tentatively identified in intact tooth matrix. ↑, emPAI at least doubled compared to intact tooth matrix; ↓, emPAI at least halved compared to intact tooth matrix.
