# Supplementary material for: In-depth, high-accuracy proteomics of sea urchin tooth organic matrix
Source: Proteome Sci. 2008 Dec 9;6:33. doi: 10.1186/1477-5956-6-33 (PMC2614417; doi:10.1186/1477-5956-6-33)
Supplement: Additional file 2 — Proteins tentatively identified in the matrix of tooth powder. Identifications with a single unique peptide showing good quality, manually validated, spectra without MS3 confirmation. [file 1477-5956-6-33-S2.doc]

Proteins tentatively identified in the matrix of tooth powder

|  |  |  |  |  |  |  |  |  |  |  |
| --- | --- | --- | --- | --- | --- | --- | --- | --- | --- | --- |
| **GLEAN3**  **accession** | **SwissProt/**  **Trembl**  **accession** | **Protein** |  | **Protein scores** | **Unique pep-tides** | **Total**  **accep-ted**  **pep-tides** | **Sequ-ence**  **cover-age** | **Gel**  **section** | **emPAI** |  |
|  |  |  |  |  |  |  |  |  |  |  |
| 11163 |  | Hypothetical LOC588710 protein; domain: CLECT | **T,S** | 114 | 1 | 11 | 8% | 9-11 | 0.6 | (I) |
| 00164 |  | Similar to SM30; domain: CLECT |  | 105 | 1 | 22 | 5% | 3-7,10,12 | 0.6 | (I) |
| 17737 |  | Similar to ECM (extracellular matrix protein) 18 |  | 98 | 1 | 3 | 12% | 12 | 0.3 | I, ↓ |
| 22347  28902 |  | Hypothetical protein LOC592275/LOC581743; domain: IG |  | 60 | 1 | 2 | 9% | 7 | 0.3 |  |
| 15869 |  | Similar to out at first protein |  | 84 | 1 | 1 | 12% | 12 | 0.3 | I |
| 22631 |  | Similar to TRIAD1 type I | **T** | 45 | 1 | 2 | 8% | 6,7 | 0.3 |  |
| 12773  17162 |  | Hypothetical LOC581130 protein; domain: Erp19 |  | 117 | 1 | 1 | 22% | 11 | 0.3 |  |
| *00465* | *Q27866*  *P69141* | *Histone sequences; contains: Histone H3, partial (aa1-90) Histone H2A (aa382-492); H3 and H2A peptide subsets match numerous other Glean3 entries coding for histone sequences* | ***T,S*** | *77* | *1* | *1* | *7%* | *1* | *0.3* | *I* |
| 01236 |  | Similar to interferon, gamma-inducible protein 30 |  | 109 | 1 | 1 | 11% | 8 | 0.3 |  |
| 27894 |  | Hypothetical protein |  | 113 | 1 | 1 | 12% | 11 | 0.2 |  |
| 13893 |  | Similar to LOC494800 protein/Sp-Cts5; domain: Peptidase_C1A_cathepsinX |  | 111 | 2 | 3 | 11% | 7 | 0.2 | (I), ↑ |
| 25926 |  | Similar to CG18405-PB; domain: partial sema | **T** | 93 | 1 | 1 | 9% | 4 | 0.2 |  |
| 20738 |  | Hypothetical protein/Sp-Ependymin-related |  | 53 | 1 | 1 | 8% | 8 | 0.2 |  |
| 06172 |  | Similar to related to cofilin |  | 78 | 1 | 1 | 7% | 10 | 0.2 | I |
| *04038*  *11065*  *16009* | *O76727* | *Integrin beta L/G subunit* |  | *52* | *1* | *2* | *4%* | *3* | *0.2* | *(I), ↑* |
| 14207 |  | Similar to IP13724p; domain: partial sema |  | 74 | 1 | 1 | 5% | 4 | 0.1 |  |
| 13716 |  | Similar to sorcin; domain:EF_hand |  | 110 | 1 | 1 | 5% | 9 | 0.1 |  |
| 20701 |  | Hypothetical protein |  | 125 | 1 | 1 | 3% | 4 | 0.1 |  |
| *00811* | *O16151* | *Similar to RAB35, peptides matches several other RAB-containing entries.* |  | *77* | *1* | *1* | *1%* | *9* | *0.1* | *I, ↓* |
| 07098 |  | Hypothetical protein LOC752806; domain: EF_hand |  | 127 | 1 | 1 | 5% | 12 | 0.1 |  |
| 27436 |  | Hypothetical LOC578301 protein |  | 90 | 1 | 2 | 5% | 9 | 0.1 |  |
| 14594 |  | Similar to 14-3-3-like protein 2 |  | 100 | 1 | 2 | 4% | 7 | 0.1 |  |
| 12548 |  | Similar to secreted protein acidic and rich in cysteine (SPARC, BM-40) |  | 61 | 1 | 2 | 3% | 8 | 0.1 | I |
| 02804 |  | Similar to mannosidase, alpha, class 2A, member 2,  Partial; domain: partial Glyco_hydro_38C |  | 48 | 1 | 2 | 3% | 3 | 0.1 |  |
| 09605 |  | Similar to tenascin R; domain: FReD |  | 65 | 1 | 3 | 3% | 7,8 | 0.1 |  |
| 06930 |  | Hypothetical protein; domains: 8 EGF_like |  | 87 | 1 | 3 | 3% | 5,6 | 0.1 | I |
| 11106 |  | Similar to annexin A4 | **S** | 83 | 1 | 1 | 2% | 7 | 0.1 | I, ↓ |
| 21651 |  | Similar to selective LIM binding factor, partial; domain: WD40 superfamily |  | 73 | 1 | 5 | 2% | 7,8,13 | 0.1 |  |
| 15781  25024  25036 |  | Similar to UDP-Gal:betaGlcNAc beta 1,3-galactosyltransferase I/Sp-B3gt1 |  | 82 | 1 | 1 | 4% | 7 | 0.1 |  |
| 16020 |  | Similar to Glutamyl aminopeptidase; domain: partial PepN |  | 59 | 1 | 1 | 2% | 4 | 0.1 |  |
| 13763 |  | Similar to family with sequence similarity 20, member C (LOC586575); domain: DUF1193 |  | 54 | 1 | 1 | 2% | 5 | 0.1 |  |
| 20268 |  | Hypothetical protein LOC582388; domain: CNPase (2',3'-cyclic-nucleotide 3'-phosphodiesterase) |  | 86 | 1 | 3 | 2% | 7,9 | 0.1 |  |
| 14914 |  | Similar to cysteine protease/Sp-Cts10, domains: Cy (cystatin_like), Inhibitor_I29, Peptidase_C1A |  | 71 | 1 | 2 | 2% | 5,9 | 0.1 | I, ↓ |
| 05691  15382 |  | Similar to Grp58-prov protein; domains 4 PDI (protein disulfide isomerase)/Sp-Pdia3 |  | 51 | 1 | 1 | 1% | 5 | 0.1 | I |
| *00796*  *28221* |  | *Similar to α-tubulin; peptide matches to several other tubulin entries* | ***S*** | *96* | *1* | *18* | *4%* | *1-4* | *0.1* | *I, ↓* |
| 21355 |  | Hypothetical protein/Sp-angiotensin-converting enzyme; domain: partial Peptidase_M2 |  | 65 | 1 | 2 | 2% | 4 | 0.1 |  |
| 08080  20850 |  | Similar to beta-1,4-galactosyltransferase 5 isoform 1 |  | 70 | 1 | 1 | 1% | 6 | 0.1 |  |
| 03084 |  | Glypican-6 |  | 60 | 1 | 1 | 1% | 7 | 0.1 | (I) |
| *05808*  *09165* |  | *Similar to 71 kDa heat shock cognate protein/similar to HSP70; domain:HSP70; peptide matches to numerous other entries containing predicted HSP70 domains* | ***T*** | *66* | *1* | *2* | *1%* | *4* | *0.1* | *I, ↓* |
| 24181 |  | Similar to protein kinase c-binding protein nell1; domains: partial TSPN, vWC, EGF_CA |  | 67 | 1 | 2 | 2% | 3,4 | 0.1 |  |
| 09922 | Q4G2F5 | Matrix metalloproteinase 16 |  | 84 | 1 | 3 | 1% | 7,8 | 0.1 | (I) |
| *17605* | *Q94760* | *Mitochondrial ATP synthase alpha subunit* |  | *75* | *1* | *2* | *1%* | *1* | *0.1* | *I* |
| 02117 |  | Similar to leishmanolysin-like (metallopeptidase M8 family); domains: Peptidase_M8, Chromo, ChSh |  | 71 | 1 | 1 | 1% | 4 | <0.1 | (I) |
| 00680 |  | Similar to ADAMTS6 variant 2; domains: ZnMc_ADAMTS_like, TSP_1; ADAM_spacer1 |  | 85 | 1 | 1 | 1% | 4 | <0.1 |  |
| 04758 |  | Hypothetical protein; domains: 4 HYR (hyaline repeat), 2 FN3 |  | 62 | 1 | 1 | <1% | 5 | <0.1 |  |
| 00152  13510 |  | Similar to neurogenic locus notch/Sp-Notch ligand 4; domains: 12 EGF |  | 68 | 1 | 1 | <1% | 2 | <0.1 |  |
| 01638 |  | Thrombospondin A/Sp-thrombospondinA1 |  | 89 | 1 | 1 | <1% | 7 | <0.1 |  |
| 05508 |  | Similar to neural adhesion molecule L1.1/Sp-FN3/Ig |  | 116 | 1 | 1 | <1% | 7 | <0.1 |  |
| *10054* |  | *Similar to myosin heavy chain* |  | *108* | *1* | *2* | *<1%* | *1,3* | *<0.1* | *I, ↓* |
| 17202 |  | Similar to kielin/tenascin-C/Sp-Ig/EGF/VWC adhesion protein; domains: 5 IG, 2 vWC, 1 partial vWF(A) |  | 96 | 1 | 1 | <1% | 2 | <0.1 |  |
| 23956  26984 |  | Hypothetical LOC581149/LOC578082 protein/Sp-QPCTL_like (glutaminyl-peptide cyclotransferase-like; domain: peptidase_M28 |  | 96 | 1 | 5 | <1% | 6,7,11 | <0.1 |  |
| 07308 |  | Similar to MEGF6¸domains: 3 partial vWA_Matrilin |  | 118 | 1 | 1 | <1% | 5 | <0.1 |  |

Proteins are ordered according to decreasing emPAI. The average absolute mass accuracy was 0.8 ppm (p<0.05). Mascot protein scores were calculated with MSQuant from unique peptide scores including MS^3^ scores. If the protein was identified in more than three gel sections only sections containing more than 5% of the total peptide number are indicated. S, also identified in spines; T, also identified in test [29]. Proteins sharing peptides with human entries are shown in *italics*. I, proteins also identified in intact tooth matrix; (I), tentatively identified in intact tooth matrix. ↑, emPAI at least doubled compared to intact tooth matrix; ↓, emPAI at least halved compared to intact tooth matrix.
