# Supplementary material for: In-depth, high-accuracy proteomics of sea urchin tooth organic matrix
Source: Proteome Sci. 2008 Dec 9;6:33. doi: 10.1186/1477-5956-6-33 (PMC2614417; doi:10.1186/1477-5956-6-33)
Supplement: Additional file 3 — Sequences of unique peptides identified in tooth powder matrix. List of sequences of accepted peptides from tooth powder matrix. [file 1477-5956-6-33-S3.doc]

**Sequences of unique peptides identified in tooth powder matrix**

| **Glean3_entry** | **Sequence** |
| --- | --- |
| *GLEAN3_00152* | *VYCGDGYFGPR* |
| *GLEAN3_00164* | *MSSGESYTATGAR* |
| GLEAN3_00204 | LCNAKPCPEESTDFR |
| GLEAN3_00204 | SSAPCELNCR |
| GLEAN3_00204 | VVDGTTCGIVSDSAICVDGMCK |
| GLEAN3_00438 | APMDLTGTYITADKPVAVVSGATCTR |
| GLEAN3_00438 | FMFAMPSNFNVASK |
| GLEAN3_00438 | TNDAFVAIPTDALGK |
| GLEAN3_00438 | VHMAGVIYTLGQR |
| GLEAN3_00438 | VMVNIPGFK |
| GLEAN3_00438 | VTLMIGSSNPGIAR |
| GLEAN3_00439 | LSLLIGANEPGLTR |
| GLEAN3_00439 | NILTNYEK |
| GLEAN3_00439 | NVDINPDVAAR |
| GLEAN3_00439 | QGPEAEYDCPETHR |
| GLEAN3_00439 | SGGFIFR |
| GLEAN3_00439 | TFTADATVPLDDVR |
| GLEAN3_00453 | GPGESVDIECR |
| GLEAN3_00453 | IPSFTPVTR |
| GLEAN3_00453 | LEANQDNSLR |
| *GLEAN3_00465* | *AGLQFPVGR histone H2A* |
| GLEAN3_00469 | FVQVPPSVLDR |
| GLEAN3_00469 | LNDVYTGDAGKPLVLECK |
| GLEAN3_00469 | QDLTSPEGAK |
| GLEAN3_00469 | SDSLIINQISK |
| GLEAN3_00469 | TDAGFYTASVTDNTR |
| GLEAN3_00469 | VSQQDLGTYICK |
| GLEAN3_00475 | GAGTDDDTLIR |
| *GLEAN3_00680* | *FCNNEYFR* |
| *GLEAN3_00796* | *AFVHWYVGEGMEEGEFSEAR α-tubulin* |
| *GLEAN3_00811* | *LQIWDTAGQER Rab-39A (I/L)* |
| GLEAN3_00983 | GLNEVAGPAEDTK |
| *GLEAN3_01236* | *YELYYESLCPGCK* |
| *GLEAN3_01638* | *LFGLVIDR* |
| GLEAN3_01796 | AIGSEGDAGR |
| GLEAN3_01796 | ASNPVSAIAGEDYAGISR |
| GLEAN3_01796 | GYPLVCVSPCNPK |
| GLEAN3_01796 | HPTYEVNENDIR |
| GLEAN3_01796 | LASDALFGVDYGR |
| GLEAN3_01796 | LTVTMPHVDGMLPIISTR |
| GLEAN3_01796 | MASITVR |
| GLEAN3_01796 | NLDFAPGVNQQTVK |
| GLEAN3_01796 | NPNVVGDTYAYQYSPELR |
| GLEAN3_01796 | QADSDTFFSTTK |
| GLEAN3_01796 | QLSNFELALSK |
| GLEAN3_01796 | SITLDSIYFGPGSR |
| GLEAN3_01796 | TCSVLLMGDNIYEPDESFR |
| GLEAN3_01796 | TGDLSIESSVR |
| GLEAN3_01796 | VANAIGAEPFTAR |
| GLEAN3_01796 | VFLCTGR |
| GLEAN3_01796 | VYIIDDR |
| GLEAN3_01796 | WMVSAPTSETGVTSPLR |
| GLEAN3_01796 | YDPAANEYGCVADTPNLLYAFK |
| GLEAN3_01796 | YTGPADPDYPNK |
| GLEAN3_01892 | EPTNFVGSMER |
| GLEAN3_01892 | LLDVDSDR |
| GLEAN3_01892 | VCGTYGSLNPR |
| *GLEAN3_02117* | *QSLSTVLLNR* |
| *GLEAN3_02804* | *GLQQGVTDNK* |
| *GLEAN3_03084* | *TFGQGLGFGR* |
| GLEAN3_03536 | GVFQTPQTFINGAVVSSEPR |
| GLEAN3_03536 | QAALSDANISEQPDSYILK |
| GLEAN3_03536 | QVIDPLLAK |
| GLEAN3_03536 | TYSWLNTAFDK |
| GLEAN3_03536 | VADYYGPDTVR |
| GLEAN3_03536 | VLSEWASEVGYSK |
| GLEAN3_03540 | GICQPIETR |
| GLEAN3_03540 | VESSLIR |
| GLEAN3_03540 | VYETESFLR |
| GLEAN3_03612 | DQSLYDQSLLK |
| GLEAN3_03612 | ICTSFLGR |
| GLEAN3_03612 | VCLCPPTVWGK |
| GLEAN3_03918 | ANPEFGAPK |
| GLEAN3_03918 | CDCDENYTLENVNDR |
| GLEAN3_03918 | HTVLISSNLDEPR |
| GLEAN3_03918 | IFDPSTSSEDYIK |
| GLEAN3_03918 | TPDGIAVDWINK |
| GLEAN3_03918 | TQLQAENEVVPVVVTDK |
| GLEAN3_03918 | TTLGICSQR |
| *GLEAN3_04038* | *LGFGSFVDK β-integrin-5* |
| GLEAN3_04105 | AGNLNYYGYR |
| GLEAN3_04105 | LGAAYFHV |
| GLEAN3_04282 | AGITRPK |
| GLEAN3_04412 | DIDECASELR |
| GLEAN3_04412 | FCGEEDPGVLVSTGNTMMLR |
| GLEAN3_04412 | TCSPDAIGSSCR |
| GLEAN3_04412 | YPGNYLDNLNCVFK |
| GLEAN3_04584 | AVEGIFVKPSEGSTVS |
| GLEAN3_04584 | EVCGLSSLSTANQWR |
| GLEAN3_04721 | ESTLHLVLR ubiquitin |
| GLEAN3_04721 | TITLEVEPSDSIENVK |
| GLEAN3_04721 | TLSDYNIQK ubiquitin |
| GLEAN3_04746 | IGAPAIAYMTPTIATTIGSTVR |
| GLEAN3_04746 | NSLAVISKPMSLNVK |
| GLEAN3_04746 | QYQDGVSTLSITPQGR |
| GLEAN3_04746 | RVSVGPVLSVTVSSISDGGR |
| GLEAN3_04746 | SNPGQLLPSGR |
| GLEAN3_04746 | VQTNGITTQAIAQLR |
| GLEAN3_04746 | VSVGPVLSVTVSSISDGGR |
| GLEAN3_04746 | YSIDISVR |
| *GLEAN3_04758* | *FNVIVEDR* |
| GLEAN3_04850 | FISLTPGYISNR |
| GLEAN3_04850 | FTGCGLPGR |
| GLEAN3_04850 | GVELAVQK |
| GLEAN3_04850 | IAPANENFPYTEK |
| GLEAN3_04850 | STDPEFIIVR |
| GLEAN3_04867 | IDPVVHDPNR |
| GLEAN3_04867 | MMVEPVPVWIGLHVGPMGR |
| GLEAN3_04867 | QPGFGNPGTPGGR |
| GLEAN3_04867 | QPGQPGVGGQPGVGGR |
| GLEAN3_04867 | QPGVGGQPGFGNPGTPGGR |
| GLEAN3_04867 | QPGVGGQPGVGGR |
| GLEAN3_04867 | QPGWGQPGVGQPGTPGGR |
| GLEAN3_05228 | FLQNSYEATVEEGTVPHK |
| GLEAN3_05228 | ISAVSPLTSDGGPTYSLQIK |
| GLEAN3_05228 | QETGDGFYPSMYR |
| GLEAN3_05228 | QVCDQEWHR |
| GLEAN3_05238 | DNGQITFAYK |
| GLEAN3_05238 | MGIADAFYIDQK |
| GLEAN3_05238 | QIPIPIASISDEAHPMK |
| GLEAN3_05296 | IGLFGGAGVGK mito ATP synthase β subunit |
| GLEAN3_05385 | GQVDLGFPR |
| GLEAN3_05385 | NELFLFK |
| GLEAN3_05385 | SFNAVAFIR |
| GLEAN3_05385 | VWSDVTPLTFR |
| GLEAN3_05420 | EITDCQSDANVICK |
| GLEAN3_05420 | HSLEGNVEISK |
| GLEAN3_05420 | SDANVLCR |
| GLEAN3_05420 | SSQDTGVTDLR |
| *GLEAN3_05508* | *LDCLNDLPAIPR* |
| GLEAN3_05538 | HPVICSGPNVMR |
| GLEAN3_05538 | IESISASTFSGAAK |
| GLEAN3_05538 | ITVGAFNGLSQLR |
| GLEAN3_05538 | LETLDLSR |
| GLEAN3_05538 | LLVPQQAGAGLLAVR |
| GLEAN3_05538 | LTNLLQDAFR |
| GLEAN3_05538 | QFYNLER |
| *GLEAN3_05691* | *ESPPIALAK* |
| *GLEAN3_05808* | *VEIIANDQGNR HSP70* |
| GLEAN3_05989 | FQHNFLTFTGGANNK |
| GLEAN3_05989 | LPFMEAQMQCLSFR |
| GLEAN3_05989 | MPGQAVYPLQGCQPGWTNFGK |
| GLEAN3_05990 | FKPDQPQQNAHR |
| GLEAN3_05990 | FQHNWLR |
| GLEAN3_05990 | FSAQAVPGQRPGFGMPPR |
| GLEAN3_05990 | GHLIVTK |
| GLEAN3_05990 | IWMGLAELPSAPESNR |
| GLEAN3_05990 | LPYDEANMFCAR |
| GLEAN3_05990 | YFWADGTEFFFTR |
| GLEAN3_05991 | DQQHNNFQHNWLR |
| GLEAN3_05991 | GYLMTSK |
| GLEAN3_05991 | MNWLQAQR |
| GLEAN3_05991 | TEMSFICQYQYML |
| GLEAN3_05991 | TNNNYFWADGTPLLPTQWNK |
| GLEAN3_05991 | VWLGLSEK |
| GLEAN3_05991 | YSALNGTTPR |
| GLEAN3_05991 | YTGGGLNK |
| GLEAN3_05992 | ELQTWVTSNWR |
| GLEAN3_05992 | TPMSYVCK |
| GLEAN3_05992 | VDQPQLNWHR |
| GLEAN3_06103 | DYQNVLR |
| GLEAN3_06103 | QISYSQVASGK |
| *GLEAN3_06172* | *VVGWTQVAVGER* |
| GLEAN3_06211 | GLFIIDDK peroxiredoxin-1 |
| GLEAN3_06387 | DQNPQGQNPNGQSPQGGVTTSR |
| GLEAN3_06387 | GGTGWNNQGNNQGPNQGPNQGPNR |
| GLEAN3_06387 | GGTGWNNQGPNQGGR |
| GLEAN3_06387 | GGTGWNNQGPQGGGAR |
| GLEAN3_06387 | QPDAIESYTASNGETYIVTANEGK |
| GLEAN3_06387 | VPNQGPNQGGR |
| GLEAN3_06387 | VPTQGPNR |
| GLEAN3_06812 | CVYGNQFISAR |
| GLEAN3_06812 | GEYYLTIR |
| GLEAN3_06812 | GTGETNVTGYR |
| GLEAN3_06812 | HGETAQFTCR |
| GLEAN3_06812 | HGPSDTVLVQGK |
| GLEAN3_06812 | KGEYYLTIR |
| GLEAN3_06812 | LIAGPTVHANSVQR |
| GLEAN3_06812 | NPPMVTVTPPEVNIK |
| GLEAN3_06812 | QEDNGVLFTCSATTPALTYPR |
| GLEAN3_06812 | TCTVMVLK |
| *GLEAN3_06930* | *IGGETTCYCPR* |
| *GLEAN3_07098* | *VSLLELTIVTGTK* |
| *GLEAN3_07308* | *LAQDGNNCEDIDECATR* |
| GLEAN3_07341 | FQVDDYIPLK |
| GLEAN3_07341 | ISYSLPDELK |
| GLEAN3_07341 | LDSENEETLTEMK |
| GLEAN3_07341 | TSQPQGQLFR |
| GLEAN3_07484 | LGITMPVVGSTGDF |
| GLEAN3_07484 | NFMIQGGDFASEDGSGSR |
| GLEAN3_07484 | TEDLPPQDEFIVEK |
| GLEAN3_07484 | TVANFLFFADPLSK |
| GLEAN3_07682 | LLMPGLYTITATAPGYEPQSR |
| GLEAN3_07682 | SFYTASPDDAVFK |
| GLEAN3_07930 | DSSGDAIVDLLDAVNSASGNQYSMELGPR |
| GLEAN3_07930 | EQYAYFYK |
| GLEAN3_07930 | FDWLISDSADTTVK |
| GLEAN3_07930 | SSDWSEISLK |
| GLEAN3_07930 | YDMVLIQEIR |
| *GLEAN3_08080* | *VAIVVPFR* |
| GLEAN3_08354 | FEGIQFGTGK |
| GLEAN3_08354 | LNPTSSFLTSGTQSFSSEEGK |
| GLEAN3_08354 | SMSTVDEGQEVR |
| GLEAN3_08354 | SVDSSILWEPR |
| GLEAN3_08354 | TRFEGIQFGTGK |
| GLEAN3_08505 | LCPTVTQGNAR |
| GLEAN3_08505 | NSNIMLNPLLR |
| GLEAN3_08505 | SLFFACK |
| GLEAN3_08505 | VAELSSNDYHEDR |
| GLEAN3_08613 | GLVLDAVDIK |
| GLEAN3_08613 | SPPSVIR |
| GLEAN3_08613 | VCIPIDPTR |
| GLEAN3_08863 | EKDVCPYNPYLSGNTDCEGK |
| GLEAN3_08863 | SGTLYWPPEAK |
| GLEAN3_08863 | SSDGTCNWMETR |
| GLEAN3_08863 | STVNVFTEPEESMCGK |
| GLEAN3_09352 | TATNLQQTSR |
| GLEAN3_09481 | AGFAGDDAPR actin |
| GLEAN3_09481 | DLTDYLMK actin |
| GLEAN3_09481 | DLYANTVLSGGSTMFPGIADR |
| GLEAN3_09481 | GYSFTTTAER actin |
| GLEAN3_09481 | IIAPPER actin |
| GLEAN3_09481 | QEYDESGPSIVHR actin |
| GLEAN3_09481 | SYELPDGQVITIGNER actin |
| GLEAN3_09481 | VAPEEHPVLLTEAPLNPK actin |
| GLEAN3_09549 | ASPGSITLAIR |
| GLEAN3_09549 | DSAGYVGFVFK |
| GLEAN3_09549 | DSPGAMGGLR |
| GLEAN3_09549 | SLYPSLEDMK |
| GLEAN3_09549 | SLYPSLEDMKVDK |
| GLEAN3_09601 | GPISCGIDATSK |
| GLEAN3_09601 | LEAYTGGIYEEFK |
| *GLEAN3_09605* | *DSQWTEIFTK* |
| GLEAN3_09606 | DGGGWTVIQR |
| GLEAN3_09606 | DILDFGGGR |
| GLEAN3_09606 | FISVSDAAK |
| GLEAN3_09606 | GATTGVGPRPPPR |
| GLEAN3_09606 | IDLQDFQYEEAHTVYR |
| GLEAN3_09606 | QSVSYGGSSVPVPLAK |
| *GLEAN3_09922* | *FDNAAAEVCR* |
| GLEAN3_10032 | DNVPLAK |
| GLEAN3_10032 | YEDLALNPVK |
| *GLEAN3_10054* | *LAGADIETYLLEK* |
| GLEAN3_10169 | ATFTCALMSTNNIGVR |
| GLEAN3_10169 | GEEELEENEGLR |
| GLEAN3_10169 | QILRPQDTNIQYTCLMR |
| GLEAN3_10169 | TCSIRPMDVPPEVQIEPAVNSVK |
| GLEAN3_10169 | VLEIQDIR |
| GLEAN3_10589 | VIEKPIYIPVSVPR |
| GLEAN3_10644 | FLSGGFQMATK |
| GLEAN3_10644 | FLTPESLYPDK |
| GLEAN3_10644 | FTLGTVLFATPVR |
| GLEAN3_10644 | IVFLLGAVR |
| GLEAN3_10644 | QSFPNLAR |
| GLEAN3_10644 | TELAENDNAIDEIR |
| GLEAN3_10644 | VDDDIIINAPLVYNILQTASPTK |
| *GLEAN3_11106* | *TGYLNLVR* |
| GLEAN3_11138 | FTDPALTGAVVVEQSMTLSR |
| GLEAN3_11138 | QLNYPGANR |
| GLEAN3_11138 | YPAGDYPMSEFAFR |
| *GLEAN3_11163* | *QQVTDILNFNQMAR* |
| GLEAN3_11180 | GKGPIVVEPVGPR |
| GLEAN3_11180 | GPIVVEPVGPR |
| GLEAN3_11180 | GVCTNDPFTGFK |
| GLEAN3_11180 | VPPSIDEEISGFIVQYNK |
| GLEAN3_11180 | VQPGAGPGNNPNTGR |
| GLEAN3_11293 | AAVEEALLR |
| GLEAN3_11293 | AGEPSLAQMTEK |
| GLEAN3_11293 | FGTLGVDDR |
| GLEAN3_11332 | ANLMSVVSR |
| GLEAN3_11332 | APPEAHVEVTGDVASLMR |
| GLEAN3_11332 | DVAILIPK |
| GLEAN3_11332 | FISESPEDSQVGIASYSNAGR |
| GLEAN3_11332 | GIWYVSIK |
| GLEAN3_11332 | GQGVEVQASEYGPPSSGR |
| GLEAN3_11332 | LLALDDVGNR |
| GLEAN3_11332 | LPGLIQK |
| GLEAN3_11332 | LQSPQTSSQTNPLLALR |
| GLEAN3_11332 | SPDFLGGANPPSADIVDTTPTFTLIR |
| GLEAN3_11332 | STTVAVVDTSR |
| GLEAN3_11332 | SYLQDMTPEGK |
| GLEAN3_11332 | TVIVIDQSATMGDDEVWQDVVR |
| GLEAN3_11332 | VQWDQNPVTVELR |
| GLEAN3_11562 | EGVCAGDTFGQSYGYK |
| GLEAN3_11562 | NGPTDGIVPVDQIDK |
| GLEAN3_11588 | DYLLALSGLDSSGSVPK |
| GLEAN3_11588 | LGSSFAFGK |
| GLEAN3_12486 | DQQAGTSSGTKPIYR |
| GLEAN3_12486 | GDSAHVGDVYVIK |
| GLEAN3_12518 | ALHGYNSAPTTK |
| GLEAN3_12518 | DMLPTDLSCFYR |
| GLEAN3_12518 | KQSPINIESR |
| GLEAN3_12518 | MFTTDNNQNAYAPR |
| GLEAN3_12518 | QSPINIESR |
| GLEAN3_12518 | TIAEAVK |
| GLEAN3_12518 | VEVSNDGHTLK |
| GLEAN3_12518 | VEYYAHLPLR |
| GLEAN3_12518 | VSTEGMYVLK |
| *GLEAN3_12548* | *ITLVDNALR* |
| GLEAN3_12695 | VTGEITEISNPVDVK |
| *GLEAN3_12773* | *ILFLNSDGVVQPDLINTLGNPQYK* |
| GLEAN3_13077 | LECICPAGLR |
| GLEAN3_13077 | LYECGPCPR |
| GLEAN3_13077 | MVPAPLVGDEYR |
| GLEAN3_13077 | NPTSDVITLYK |
| GLEAN3_13077 | YAEAVFNFK |
| GLEAN3_13669 | DNEVYFVTPK |
| GLEAN3_13669 | GNDAVSNALQTESVFVDMQDIPDAAFIR |
| GLEAN3_13669 | GRPGPFNAVWTENK |
| GLEAN3_13669 | GRPGPFNAVWTENKDR |
| GLEAN3_13669 | IWEFSQGVYQPR |
| GLEAN3_13669 | MVFFSGSK |
| GLEAN3_13669 | RGNDAVSNALQTESVFVDMQDIPDAAFIR |
| GLEAN3_13669 | TSYFLIGGQVSTFK |
| GLEAN3_13670 | ADIIVQFAR |
| GLEAN3_13670 | DNEVFFIAETR |
| GLEAN3_13670 | FTLNTDDVR |
| GLEAN3_13670 | GGVSYFLIDK |
| GLEAN3_13670 | GIQSLYGAR |
| GLEAN3_13670 | GTEPVSNALR |
| GLEAN3_13670 | GYMQIYEYVEGAETPEELR |
| GLEAN3_13670 | KGTEPVSNALR |
| GLEAN3_13670 | LDFNPTR |
| GLEAN3_13670 | NGPINAAWTEGK |
| GLEAN3_13670 | QVSTFKDNEVFFIAETR |
| GLEAN3_13670 | SLMAPYYQGFQPR |
| GLEAN3_13670 | TEAVFTEMSAVPDAAFIR |
| GLEAN3_13670 | TNGIDAAFK |
| GLEAN3_13670 | YAIQHGWSK |
| *GLEAN3_13716* | *LSPNAINALVR* |
| GLEAN3_13756 | DFMIQGGDFTK |
| GLEAN3_13756 | DTNGSQFFLTTIK |
| GLEAN3_13756 | IVIGLFGQTVPK |
| GLEAN3_13756 | VLEGMDVVR |
| *GLEAN3_13763* | *DLFELADPAFR* |
| GLEAN3_13821 | DGHIDASYNALYK |
| GLEAN3_13821 | ELCSVEVGSQPIAVR |
| GLEAN3_13821 | FNAPAEVQR |
| GLEAN3_13821 | GPECESLAVGDVQGR |
| GLEAN3_13821 | GQTGGMYALNNGVAFK |
| GLEAN3_13821 | IDASSADR |
| GLEAN3_13821 | IFNGAAFSRPPQR |
| GLEAN3_13821 | IYEKYNPATMMMK |
| GLEAN3_13821 | KTGDLSPESLSFIPPEK |
| GLEAN3_13821 | KYVNPEGTITTVR |
| GLEAN3_13821 | LPVGGGAGGAGGAGGAGGGGGGGGGAGGR |
| GLEAN3_13821 | LYLPFDK |
| GLEAN3_13821 | LYLPFDKLPVGGGAGGAGGAGGAGGGGGGGGGAGGR |
| GLEAN3_13821 | QIATEGPVADIAECGDLVAFTQPGKPHFTDVGSLK |
| GLEAN3_13821 | QLAYVGGGQFVQIVDFSDVVQPK |
| GLEAN3_13821 | QPYTGQLGDPGPHTFSR |
| GLEAN3_13821 | SQTNKPLLLVTGR |
| GLEAN3_13821 | TGDLSPESLSFIPPEK |
| GLEAN3_13821 | YNPATMMMK |
| GLEAN3_13821 | YVNPEGTITTVR |
| GLEAN3_13822 | CDTIIIANEGPAAENVEQEMFVNPEGTVSVVR |
| GLEAN3_13822 | LFNDPALAPLMEQQFIR |
| GLEAN3_13822 | TDPGMVHIYQK |
| GLEAN3_13822 | VIIVGIK |
| GLEAN3_13822 | VSGTVNLYR |
| GLEAN3_13823 | DQNPQGQNPNGQPPQGGVTTSR |
| GLEAN3_13823 | EFGDSDPESITFIPPEK |
| GLEAN3_13823 | FDSPRPVTDIAECGR |
| GLEAN3_13823 | GDEFPEDYWLTAK |
| GLEAN3_13823 | GPGGGGHGMNPPNR |
| GLEAN3_13823 | GTCSGGGNFNPTVTTLDFTK |
| GLEAN3_13823 | HDDVYFYGGR |
| GLEAN3_13823 | ILDKFDSPRPVTDIAECGR |
| GLEAN3_13823 | ISAIALFSVPPDGNLPIFESIHR |
| GLEAN3_13823 | LAYSAGK |
| GLEAN3_13823 | LGNLEFSR |
| GLEAN3_13823 | LIFVGIDR |
| GLEAN3_13823 | MGPECESIEIGDVQGTK |
| GLEAN3_13823 | NCEFIVGSRPK |
| GLEAN3_13823 | QPDAIESYTAANGETYIVTANEGK |
| GLEAN3_13823 | QPYTGQMGDK |
| GLEAN3_13823 | STEQPLKHDDVYFYGGR |
| GLEAN3_13823 | TKEFGDSDPESITFIPPEK |
| GLEAN3_13823 | VGDPSLTMASTFDSQSDK |
| GLEAN3_13823 | VSGTITIYQIR |
| GLEAN3_13823 | VSNAEMLDAFR |
| GLEAN3_13823 | YDIESFR |
| GLEAN3_13823 | YDLDPSPGNAR |
| GLEAN3_13825 | AENALGWR |
| GLEAN3_13825 | FWTGFYK |
| GLEAN3_13825 | IMLSYLTTVFGQGR |
| GLEAN3_13825 | MTFNEATFFCNR |
| GLEAN3_13825 | QKFWTGFYK |
| GLEAN3_13825 | YGGSLYALDSPSK |
| GLEAN3_13825 | YGGSLYALDSPSKNR |
| *GLEAN3_13893* | *GPMMAEISAR* |
| *GLEAN3_13893* | *SGAGDSYNLGIETECAYGVPIVN* |
| *GLEAN3_14207* | *APTNFVGSIER* |
| GLEAN3_14421 | LELPYIVSNR |
| GLEAN3_14421 | VAEDGQFSVQQIHR |
| *GLEAN3_14594* | *FLIPIAENEIK* |
| GLEAN3_14602 | FAPTETEPMR |
| GLEAN3_14602 | LQDCPVAPGSQPPTR |
| GLEAN3_14602 | LQGSDSTVQGR |
| GLEAN3_14602 | LVSNDYSFLQGK |
| GLEAN3_14602 | QVGDLAAVEATTGMR |
| GLEAN3_14602 | SPSEGYVAVYDGLK |
| *GLEAN3_14914* | *FADMTEAEFR* |
| GLEAN3_15124 | AELNAMMTEELGFYK |
| GLEAN3_15124 | GNLAEDVPDEHSTGPYSSK |
| GLEAN3_15125 | TGSSTVQNIFLR |
| GLEAN3_15125 | TNVTFLK |
| GLEAN3_15321 | GYLTPFGDCIK |
| GLEAN3_15321 | IPSTIPTSCGPFK |
| GLEAN3_15321 | YQSVIAANGIYYR |
| GLEAN3_15404 | EVVQIVSR |
| GLEAN3_15404 | GTLTVGPLR |
| GLEAN3_15404 | TCTEDVGATFK |
| GLEAN3_15404 | YSLTLPVGQK |
| *GLEAN3_15781* | *GVVIGNVNLDMPVVR* |
| GLEAN3_15848 | FNSISVTPQPIAMPGTIR |
| GLEAN3_15848 | IAVNVEIVR |
| GLEAN3_15848 | NILQTMSAEITVSR |
| *GLEAN3_15869* | *AEFISSDAMSK* |
| GLEAN3_15906 | FDYQDSR |
| GLEAN3_15906 | FEDQNPNQIPIR |
| GLEAN3_15906 | GYVSFGYK |
| GLEAN3_15906 | HEDKVFPIFPEYSTK |
| GLEAN3_15906 | SHADEGALTVR |
| GLEAN3_15906 | TDIKFEDQNPNQIPIR |
| GLEAN3_15906 | VFPIFPEYSTK |
| GLEAN3_15906 | WTITASNR |
| GLEAN3_16016 | GYSTTIIEAK |
| GLEAN3_16016 | LVSYANAANR |
| *GLEAN3_16020* | *LLYGMAQTR* |
| GLEAN3_16052 | AIGNMGLIQSK |
| GLEAN3_16052 | DQNPGLLR |
| GLEAN3_16052 | ELPLQVGGTAIIR |
| GLEAN3_16052 | GFNDVFVK |
| GLEAN3_16052 | ICPEVETNEVIK |
| GLEAN3_16052 | IFSTIDNDSELR |
| GLEAN3_16052 | IGDSIFLK |
| GLEAN3_16052 | ISGPTSDQSSTVR |
| GLEAN3_16052 | LAANYPFR |
| GLEAN3_16052 | LDNLVVR |
| GLEAN3_16052 | MCSIAVAYK |
| GLEAN3_16052 | MDTEYTLTPSR |
| GLEAN3_16052 | MSGLEAFK |
| GLEAN3_16052 | NMINLVK |
| GLEAN3_16052 | VAAIQAYR |
| GLEAN3_16497 | FYDYETNSCEEGR |
| GLEAN3_16497 | SPGWPAR |
| GLEAN3_16497 | WALPETTSSSR |
| GLEAN3_16506 | ANNAVVQLILTPGNEAITGFNPMGMK |
| GLEAN3_16506 | APMDGEFSILMDNK |
| GLEAN3_16506 | AQQQQQQGGQPNYPGQGAGAGTR |
| GLEAN3_16506 | DLASTER |
| GLEAN3_16506 | FATTQGNCAAQFGHR |
| GLEAN3_16506 | FFHNCETIVIANK |
| GLEAN3_16506 | GTPAADAATNTFTDPEGTISIVR |
| GLEAN3_16506 | GVAETYPQVFNSK |
| GLEAN3_16506 | KAPMDGEFSILMDNK |
| GLEAN3_16506 | KSGTLSMYR |
| GLEAN3_16506 | KWNLLCDAK |
| GLEAN3_16506 | MPYDGSMDGSRPTLSK |
| GLEAN3_16506 | MTVTTIDFR |
| GLEAN3_16506 | NMGDIDPAK |
| GLEAN3_16506 | NPQNPNSGYDYVSFFGGR |
| GLEAN3_16506 | NQYNLASYLFTADEGATTSYR |
| GLEAN3_16506 | QPNAIESFEIR |
| GLEAN3_16506 | SATPDNYNYLMVMGK |
| GLEAN3_16506 | SFTDAVPFSR |
| GLEAN3_16506 | SGTLSMYR |
| GLEAN3_16506 | STTADAQSHGPK |
| GLEAN3_16506 | TWFNNDLDASSTPR |
| GLEAN3_16506 | VGADPVSLK |
| GLEAN3_16506 | WNLLCDAK |
| GLEAN3_16506 | YNMYSMR |
| GLEAN3_16731 | QPADMFTMDELR |
| GLEAN3_16731 | TTNAIDVFPENYR |
| *GLEAN3_17202* | *TFTEATSDNTVR* |
| GLEAN3_17586 | ANCDGSLVQR |
| GLEAN3_17586 | CPANMVK |
| GLEAN3_17586 | YACFTNTPK |
| GLEAN3_17587 | APAPPAPVPR |
| GLEAN3_17587 | APAPPAQAPR |
| GLEAN3_17587 | APAPQAPAR |
| GLEAN3_17587 | APAPSAPR |
| GLEAN3_17587 | APAPSVPR |
| GLEAN3_17587 | APAYVPR |
| GLEAN3_17588 | AAPGFAPPGFAPPAPR |
| GLEAN3_17588 | APAPGHSPGYPPAR |
| GLEAN3_17588 | APAPPMPR |
| GLEAN3_17588 | APAPYPPAR |
| GLEAN3_17588 | APGGGGGQIPAR |
| GLEAN3_17588 | APGVAQAAPR |
| GLEAN3_17588 | APPPAAAPGSFAPSPGYSAPAGGR |
| GLEAN3_17588 | APSVPAVPRPR |
| GLEAN3_17588 | APTPGHPGYQPPR |
| GLEAN3_17588 | APTRPGVPVRPYAPGPR |
| GLEAN3_17588 | CDLEEEDAYTQQNIR |
| GLEAN3_17588 | DSCDLGTPGAACGSDGQSWGAGGTPAR |
| GLEAN3_17588 | FVPVVVFPSK |
| GLEAN3_17588 | GGVGVAR |
| GLEAN3_17588 | LYNDKCDLEEEDAYTQQNIR |
| GLEAN3_17588 | RPSAPSVPYAPSGGR |
| GLEAN3_17589 | APAPPTAPGAPYAPSAPLPPQTPYAPPAPR |
| GLEAN3_17589 | APQTPYAPQAPR |
| GLEAN3_17589 | APTFGSPALPR |
| GLEAN3_17589 | APVVAPPLIPR |
| GLEAN3_17589 | APYAPPVPAAPR |
| GLEAN3_17589 | SGVSISVNHK |
| GLEAN3_17589 | TTFQVPR |
| GLEAN3_17589 | TYHNECDVEDDSSR |
| GLEAN3_17589 | VPFPAPR |
| GLEAN3_17589 | VPQVPQTPYTPQAPR |
| GLEAN3_17590 | APAPPAPPAAPAAPAAPAAPAPK |
| GLEAN3_17590 | APAPQAPVVPYAPAVPR |
| GLEAN3_17590 | APGLPFAPQAR |
| GLEAN3_17590 | APSPAAPSAPR |
| GLEAN3_17590 | APVQVPR |
| GLEAN3_17590 | QGETGYQAPVAPR |
| *GLEAN3_17605* | *HALIIYDDLSK mito ATP synthase α subunit* |
| *GLEAN3_17737* | *LISEGGSLCTK* |
| GLEAN3_18054 | EYYEVNETER |
| GLEAN3_18054 | GEDLAYCR |
| GLEAN3_18054 | LSNPMGGR |
| GLEAN3_18054 | MEEDPDNMVAFDK |
| GLEAN3_18054 | QVQFNPGQTEGFWR |
| GLEAN3_18348 | GCLQNVR |
| GLEAN3_18348 | GELSSDFAK |
| GLEAN3_18348 | MVNWGGAPVGTGR |
| GLEAN3_18348 | TFQANANSDDVIK |
| GLEAN3_18348 | TTLPEPVEGR |
| GLEAN3_18348 | TTVDPSDPSDPATAAVFVAADAEK |
| GLEAN3_18348 | VVAVATQGR |
| GLEAN3_18348 | YNSNEFVK |
| GLEAN3_18406 | FPNIGTGGYPGSVFPHGPGYPR |
| GLEAN3_18406 | GGGTIPGVSAGAGGGVGGGGGGVRPGKPNFGLPGSGGGGTGFGGGPGGAG |
|  | GAGGAGGAGGAGGR (cont.) |
| GLEAN3_18406 | GGGYGGAGGR |
| GLEAN3_18406 | GIHPAAGGPAYNGR |
| GLEAN3_18406 | GTGSGSRYPGYNPR |
| GLEAN3_18406 | GVGGAGGAGGGTGAAGR |
| GLEAN3_18406 | HIPGYIPGR |
| GLEAN3_18406 | NGLPVGGPGFR |
| GLEAN3_18406 | NPSMTDPK |
| GLEAN3_18406 | RPSASDSGSGGTGVNGGTGGGAR |
| GLEAN3_18406 | RTGVGILPDIQVIDPR |
| GLEAN3_18406 | STYPGQNYPGSR |
| GLEAN3_18406 | TGVGILPDIQVIDPR |
| GLEAN3_18406 | YPGYNPR |
| GLEAN3_18406 | YPNVGNPGMNYPGGYPGVGVGGFPGQGGYPGNNYPGQNYPGNNFPGSR |
| GLEAN3_18452 | EVAVEYTSGDAVYSR |
| GLEAN3_18452 | SQGPQNVLLTK |
| GLEAN3_18702 | DLPVIAIDSFR |
| GLEAN3_18702 | SYEDAEYTAFR |
| GLEAN3_18768 | AYIEASPILLIK |
| GLEAN3_18768 | IENVMFAK |
| GLEAN3_18810 | ELGTIPGR |
| GLEAN3_18810 | LVASFSQDNQMER |
| GLEAN3_18810 | NPFGMPPGFAPVMR |
| GLEAN3_18810 | RELGTIPGR |
| GLEAN3_18810 | SPQENMEIYR |
| GLEAN3_18811 | AFVCEVPAGR |
| GLEAN3_18811 | MASEFCEMVTPCGNGPAK |
| GLEAN3_18811 | NIPIGQQPGMGQGGFGNQQPGMGGR |
| GLEAN3_18811 | QIPQGVGPQWEAVEVTAMR |
| GLEAN3_18811 | QPGFGNQPGMGGQQPGMGGQQPGWGNQPGVGGR |
| GLEAN3_18811 | QPGFGNQPGMGGR |
| GLEAN3_18811 | QPGFGNQPGVGGR |
| GLEAN3_18811 | QPGMGGQPGVGGR |
| GLEAN3_18811 | QPGMGGQQPGMGGQPGVGGR |
| GLEAN3_18811 | QPGMGGQQPGWGNQPGVGGR |
| GLEAN3_18811 | QPGMGGQQPNNPNNPNPNNPNNPNNPNPR |
| GLEAN3_18811 | QPGMGGR |
| GLEAN3_18811 | QPGVGGR |
| GLEAN3_18811 | QPGWGNQPGVGGR |
| GLEAN3_18811 | RQNPPVRPGQGGR |
| GLEAN3_18811 | SWPVNPQNPMSGPPGR |
| GLEAN3_18813 | CMTLPGQMQMNGAVQK |
| GLEAN3_18813 | FDLLRPIGGNR |
| GLEAN3_18813 | GGWGAGAGTGQGAGGGWGGQNPQNPGAGGGR |
| GLEAN3_18813 | GGWGQGGQGQGGQGGR |
| GLEAN3_18813 | LRPSNVWMGFR |
| GLEAN3_18813 | TFTGCDGISPGHLAAPTTFEER |
| GLEAN3_18813 | TYDMASQSCK |
| GLEAN3_18813 | VENSCYR |
| GLEAN3_18813 | WNPNQGAGAGAGAGGR |
| GLEAN3_18813 | WNPNQGAGAGAGAGGRWNPNQGAGAGAGAGGR |
| GLEAN3_18813 | WNPQTPQNPGQGGR |
| GLEAN3_18919 | EISSGEGEQPK |
| GLEAN3_18919 | EMSSGQVEEQPK |
| GLEAN3_18919 | EVSSGQVEELK |
| GLEAN3_18964 | GWDQGLLNMCEGEK |
| GLEAN3_18964 | LEDGTEFDSSIPR |
| GLEAN3_18964 | LVIPSNLGYGDR |
| GLEAN3_18964 | QTFDFTLGAGQVIK |
| GLEAN3_19034 | IDTNVILSTDGK |
| GLEAN3_19655 | FMPFKPLLAK |
| GLEAN3_19695 | DGTSQYDLAPLSK |
| GLEAN3_19695 | LESLSDDELK |
| GLEAN3_20031 | VITGYLIR |
| *GLEAN3_20268* | *VIGLLVTPR* |
| GLEAN3_20457 | FTCVATNPALQQPSTCSLTPLR |
| GLEAN3_20457 | GGDPPATLSWVR |
| GLEAN3_20457 | IEITGDVDR |
| GLEAN3_20457 | LNPYGQVGEEQLSR |
| GLEAN3_20612 | CPVGLYGPYCR |
| GLEAN3_20612 | DPVVASPPR |
| GLEAN3_20612 | TGTDTASDTASSSSSSSSSHSVSTTGSK |
| GLEAN3_20612 | VPAPSGPR |
| GLEAN3_20612 | YPAADPNDGTSTSK |
| *GLEAN3_20701* | *VTVIVVK* |
| *GLEAN3_20738* | *ASEWDHIQGVNNR* |
| GLEAN3_20773 | DILIGDYVDSYR |
| GLEAN3_20773 | TVFLIGK |
| GLEAN3_21260 | DVLSYPR |
| GLEAN3_21260 | IVISDPGNGLVVSMNLDGSLPK |
| GLEAN3_21260 | QVSSTSHTEPTSLSIDETNQQIYVAYK |
| GLEAN3_21260 | VISSLSSNYK |
| GLEAN3_21260 | VLAATLGAYR |
| *GLEAN3_21355* | *IFDGTTPESK* |
| GLEAN3_21385 | CGFLVAWITR |
| GLEAN3_21385 | FARPNIVR |
| GLEAN3_21385 | FIQVVDYSR |
| GLEAN3_21385 | ITDPGTVHVWDMYR |
| GLEAN3_21385 | TTAPAADIAR |
| GLEAN3_21385 | YDQGAANSVAFDPASSFAYVAGNK |
| GLEAN3_21385 | YIPSDYINNGVGEHK |
| GLEAN3_21428 | AFTNAGEGPASDVVEVVTDSAPPGPVYK |
| GLEAN3_21428 | VDVYSYR |
| GLEAN3_21428 | VTNQEGSTVSTTTEFK |
| GLEAN3_21559 | AEILLDQYR |
| GLEAN3_21559 | IAPVPITDANVASK |
| GLEAN3_21559 | MGLEAMIIQR |
| *GLEAN3_21651* | *VFNWAPR* |
| GLEAN3_21853 | LASVQACNAHR |
| GLEAN3_22057 | IVDQVSGIAR |
| GLEAN3_22057 | IVVDTVQGR |
| GLEAN3_22057 | NALYVGLPEAIVK |
| GLEAN3_22278 | APLPAPPAPR |
| GLEAN3_22278 | EEGNTRPNASLK |
| GLEAN3_22278 | KVNLFNE |
| GLEAN3_22278 | QGDTPEAPR |
| *GLEAN3_22347* | *ATMFLEVADA* |
| GLEAN3_22598 | ETDGPPFYSYK |
| GLEAN3_22598 | IVGPAPLDCHEVR |
| GLEAN3_22598 | LQQEMDELK |
| GLEAN3_22598 | QQQIITLETR |
| GLEAN3_22598 | VEVTDWFNR |
| GLEAN3_22598 | YIADYSGFEVDSK |
| *GLEAN3_22631* | *YPFQPGLGGR* |
| GLEAN3_22672 | NLVTLLTTK |
| GLEAN3_23016 | AIDPNTELVINVEEGDLCTIK |
| GLEAN3_23016 | EDYIPLVVELQNLDDGQVVK |
| GLEAN3_23016 | FDITDGTNPLIDR |
| GLEAN3_23016 | FTDMDSNDR |
| GLEAN3_23016 | GGMPISAFSIK |
| GLEAN3_23016 | GTTVIDNTMLDAIDPDTDR |
| GLEAN3_23016 | GVEPEEDQFTFR |
| GLEAN3_23016 | IIDQPLLR |
| GLEAN3_23016 | ISFTDIDNK |
| GLEAN3_23016 | ISFTDIDNKEPVVR |
| GLEAN3_23016 | IYYVHTAEDEIR |
| GLEAN3_23016 | LITPFELAIDDR |
| GLEAN3_23016 | LITVDEGGQR |
| GLEAN3_23016 | LVPSNFPCDFGPGEVK |
| GLEAN3_23016 | NQPITSFTQK |
| GLEAN3_23016 | NTGISVPFGR |
| GLEAN3_23016 | QEYFQSMVR |
| GLEAN3_23016 | QLEIVTR |
| GLEAN3_23016 | QPQQVPIEIVPVDNGAPQIVVNR |
| GLEAN3_23016 | QSEPLSQIPGR |
| GLEAN3_23016 | VIVVQPPQFGVVSLDGIPLQK |
| GLEAN3_23016 | VSYRPPSTELGIAPR |
| GLEAN3_23016 | VTVLSSSSGLPR |
| GLEAN3_23016 | VVDSSDPQPNESPVQEFLVK |
| GLEAN3_23016 | YGPEHGLLQR |
| GLEAN3_23016 | YVHTSDVGGR |
| GLEAN3_23052 | CYQCFGLGSR |
| GLEAN3_23052 | DFSQLPNIDEVR |
| GLEAN3_23052 | HPIASCTDIGPQSQTPTIDCTGK |
| GLEAN3_23052 | NQLLAQTASK |
| GLEAN3_23115 | AAAPGQVMVVDVMER |
| GLEAN3_23115 | HGPEVIYHYR |
| GLEAN3_23115 | LSNLEPYSTYDVYVQAVSDAGMAPVATR |
| GLEAN3_23115 | TLDSIELR |
| GLEAN3_23115 | VPAPISPK |
| GLEAN3_23115 | VTYVLTNLGMCGPISSSLR |
| GLEAN3_23115 | WNTLECGDR |
| GLEAN3_23289 | GMLEASAIVPSK |
| GLEAN3_23289 | SVIGTDPTVTCVYDHK |
| GLEAN3_23330 | LGMLVDLSHVSVK |
| GLEAN3_23330 | MLYELGVR |
| GLEAN3_23330 | NAPNSILTR |
| GLEAN3_23855 | WLSQSSLR |
| GLEAN3_23855 | YASYLNFGNEK |
| *GLEAN3_23956* | *VWSGSSTTPYFK* |
| GLEAN3_24019 | IDATSQLR |
| GLEAN3_24019 | LQDASSSIIIQPTR |
| *GLEAN3_24181* | *TIEANEETYQK* |
| GLEAN3_24565 | LNELQTVEDEGR |
| GLEAN3_24565 | TSDILSDTTK |
| GLEAN3_25068 | YNTPGEGGLTK |
| GLEAN3_25235 | DPGQVTEGVR |
| GLEAN3_25235 | DTCAQNPCFEGVR |
| GLEAN3_25235 | GICQPICER |
| GLEAN3_25235 | INVNTLDYTSQEEAK |
| GLEAN3_25235 | INVNTLDYTSQEEAKR |
| GLEAN3_25235 | LPVPNDANIVVVASK |
| GLEAN3_25235 | LTCVDDNECQSNR |
| GLEAN3_25235 | LTEDGLGCEDVNECEEGLAK |
| GLEAN3_25235 | NNENNLVTLTLTR |
| GLEAN3_25235 | NSICTYGAFK |
| GLEAN3_25235 | TFTFTDPNTPDGQLQVEIPAR |
| GLEAN3_25235 | TGTIVTFVK |
| GLEAN3_25235 | VSETVNYACK |
| GLEAN3_25502 | DAILYTPR |
| GLEAN3_25502 | DGTGPASENAVAK |
| GLEAN3_25502 | LNIADTLPESAGR |
| GLEAN3_25502 | VSGTLQINNVR |
| GLEAN3_25722 | FKDESQLVTEPIR |
| GLEAN3_25722 | LTELFPK |
| GLEAN3_25722 | QPITISR |
| GLEAN3_25772 | SLAPQYSIAAK |
| *GLEAN3_25926* | *NSAGIEGVSCPGPR* |
| GLEAN3_25966 | ADAMPENAALK |
| GLEAN3_25966 | CAFPAITTLPK |
| GLEAN3_25966 | ELFLQDNLINSVSR |
| GLEAN3_25966 | GAFSSMTQLQTLR |
| GLEAN3_25966 | IDTSELVCGTPFGVR |
| GLEAN3_25966 | KYGGSLCQILKPGNPTK |
| GLEAN3_25966 | LDINFLK |
| GLEAN3_25966 | LDLSHNYITEIAPDTFK |
| GLEAN3_25966 | QQATYDPIGHLK |
| GLEAN3_25966 | SPQQTYSLGPGAYR |
| GLEAN3_25966 | TLQLADNPLER |
| GLEAN3_25966 | TVSTGATFILPCTLTAQAAAR |
| GLEAN3_25966 | VDGAMFSR |
| GLEAN3_25966 | VFYSLVGDR |
| GLEAN3_25966 | YGGSLCQILKPGNPTK |
| GLEAN3_26000 | CIVILPSGVK |
| GLEAN3_26000 | FLVSADR |
| GLEAN3_26000 | FSIAGDSNLGEHDLMIR |
| GLEAN3_26000 | LIGESTLK |
| GLEAN3_26000 | NQPDTVTVYAGDTIYMR |
| GLEAN3_26000 | NVDYEDAGMYWCQASGAGGVIESVAGR |
| GLEAN3_26000 | SDNTNPQTQYISIR |
| GLEAN3_26000 | SEFTVIR |
| GLEAN3_26000 | VLPADTQPAAVNPR |
| GLEAN3_26000 | VPIPIPTCSMTPPQPSVGQR |
| GLEAN3_26000 | YSDGSIFFTR |
| GLEAN3_26000 | YVTEGSSAMFGCQSR |
| GLEAN3_26042 | CSVGCGTGTQTR |
| GLEAN3_26042 | GVLCSTEGTTAGAVPDGQCSGQQKPDEVK |
| GLEAN3_26042 | LCSYSYYR |
| GLEAN3_26042 | WFTSEWSR |
| GLEAN3_26072 | AVMFGVGADDAR |
| GLEAN3_26072 | IEELDDAPIR |
| GLEAN3_26072 | LNEEYEDLDFR |
| GLEAN3_26072 | SSFSSFSTLR |
| GLEAN3_26094 | AIAYYTESISK |
| GLEAN3_26094 | ISGLDPAGPGFGGK |
| GLEAN3_26146 | DSYNTVFANFEK |
| GLEAN3_26949 | ATDSTLTLETLK |
| GLEAN3_26949 | AVGEFFSAGSCAPGAK |
| GLEAN3_26949 | CLADQAGDVAFVK |
| GLEAN3_26949 | GANLTPQISCYEETSK |
| GLEAN3_26949 | GLMSSDFR |
| GLEAN3_26949 | MCPANSLR |
| GLEAN3_26949 | NANEPYYDYAGAFR |
| GLEAN3_26949 | WCTTSSIENK |
| GLEAN3_26949 | YAGIAVVR |
| GLEAN3_27046 | QVYQEVNDLER |
| GLEAN3_27169 | IPDYWVVDNER |
| GLEAN3_27169 | MQAPAFGLASTTFK |
| GLEAN3_27172 | AVAVDLPGYGESK |
| GLEAN3_27172 | AVVEGQAETTQVCK |
| GLEAN3_27172 | DESIGLESLK |
| GLEAN3_27172 | EVLPATEPK |
| GLEAN3_27172 | GFVPVAPVGTNK |
| GLEAN3_27172 | GTLLFLHGMR |
| GLEAN3_27172 | LTNEEYGAIEVPTLIVYGEKDESIGLESLK |
| GLEAN3_27172 | QAVIISPSMSGSFSIPFLK |
| GLEAN3_27236 | AVPSSYSDLGK |
| GLEAN3_27236 | ITLSTLIDGK |
| GLEAN3_27236 | LTLDTSFSPQTGK |
| *GLEAN3_27436* | *ICIEDITTDAATR* |
| GLEAN3_27885 | TYASGVTPAMK |
| GLEAN3_27885 | YNSDFPVEELR |
| *GLEAN3_27894* | *QTGTVLTANAMNPFGTK* |
| GLEAN3_27906 | FFPMPLIK |
| GLEAN3_27906 | LLDIADFDSFR |
| GLEAN3_27906 | NLYTHTLPFYQPPTGQQLDFIPPEK |
| GLEAN3_27906 | NPGNPNQPVR |
| GLEAN3_27906 | RPNQPVWPR |
| GLEAN3_28030 | ISLDFPIR |
| GLEAN3_28030 | QVVTTDLR |
| GLEAN3_28091 | TPIVNFFDR |
| GLEAN3_28091 | VTGVITQGR |
| GLEAN3_28748 | TYDAITLIR |
| GLEAN3_28748 | VWEEVTPLK |
| GLEAN3_28749 | GAPSNIDAIFEKPGGTTVMIK |
| GLEAN3_28749 | GELYTFSGALMWR |
| GLEAN3_28749 | MGLSNGISAAFSWPQDR |
| GLEAN3_28749 | NSQLVQGYPVR |
| GLEAN3_28749 | NVLEDIPGLPLGIDAAFSSK |
| GLEAN3_28749 | QRVDNNEYPR |
| GLEAN3_28749 | TASYFVR |
| GLEAN3_28749 | TTITFTFDNYTPDLPMNQVR |
| GLEAN3_28749 | VDNNEYPR |
| GLEAN3_28749 | VWSDVTPLK |
| GLEAN3_28749 | YDHSSGSLSQGFPR |
| GLEAN3_28749 | YVLSGASWGR |
| GLEAN3_28749 | YWEYSGVNLKPGFPR |
| GLEAN3_28887 | LQDDKTEADHLTYF |
| GLEAN3_28887 | LYKPFQGEITFNK |
| GLEAN3_28887 | SFQDADAGISAEDR |
| GLEAN3_28887 | VRGEAEEAAGR |
|  |  |

Tentative identifications are shown in italics. Peptides shared with human proteins are shaded yellow. Peptides are arranged according to increasing Glean3 entry number.
