# Supplementary material for: In-depth, high-accuracy proteomics of sea urchin tooth organic matrix
Source: Proteome Sci. 2008 Dec 9;6:33. doi: 10.1186/1477-5956-6-33 (PMC2614417; doi:10.1186/1477-5956-6-33)
Supplement: Additional file 4 — Proteins identified in demineralized intact teeth. List of proteins identified in the organic matrix of demineralized hypochlorite-treated intact teeth. [file 1477-5956-6-33-S4.doc]

**Proteins identified in organic matrix of intact teeth**.

|  |  |  |  |  |  |  |  |  |  |  |
| --- | --- | --- | --- | --- | --- | --- | --- | --- | --- | --- |
| **GLEAN3**  **accession** | **SwissProt/**  **Trembl**  **accession** | **Protein** |  | **Protein scores** | **Unique pep-tides** | **Total**  **accep-ted**  **pep-tides** | **Sequ-ence**  **cover-age** | **Gel**  **section** | **emPAI** |  |
|  |  |  |  |  |  |  |  |  |  |  |
| 18811 | P11994 | Spicule matrix protein SM50 | **T,S** | 1558 | 14 | 988 | 61% | 3-9 | 31.7 | P, ↓ |
| *00685* |  | *Similar to histone H4; the same peptide set matches to >60 Glean3 histone entries* | ***T,S*** | *414* | *4* | *79* | *40%* | *1,2,7-12* | *3.9* |  |
| 21385  16506 | Q8MUK7 | MSP130-related-2; entries cover different but overlapping regions | **T,S** | 2451 | 23 | 532 | 46% | 3-12 | 3.0 | P, ↓ |
| 18813 | O76450 | Spicule matrix protein SM37 | **T,S** | 764 | 7 | 154 | 29% | 4-7,10,11 | 2.6 | P, ↓ |
| 18406  18407 |  | Hypothetical protein; Gly-rich; gene close to P16 gene, PMC-specific expression | **T,S** | 1167 | 11 | 146 | 47% | 7-11 | 2.5 | P, ↓ |
| 13821 | P08472 | (Similar to) MSP130 | **S,T** | 2008 | 16 | 590 | 28% | 3-9 | 2.2 | P |
| 05990 | Q8MUL0 | Spicule matrix protein SM29 | **T,S** | 581 | 6 | 279 | 27% | 6-12 | 2.2 | P, ↓ |
| *09481* |  | *Actin; peptide set matches many actin entries* | ***S*** | *1245* | *9* | *91* | *30%* | *4,6,7,11,12* | *1.9* | *P* |
| 17589 |  | Hypothetical protein; domains: Kazal_follistatin, AP-rich and DE-rich motifs |  | 1060 | 10 | 198 | 29% | 1-4 | 1.8 | P |
| 04867 |  | Similar to spicule matrix protein SM30/SM30-E | **T,S** | 624 | 7 | 372 | 21% | 3-9 | 1.5 | P, ↓ |
| *16845*  *16946*  *19138*  *23661*  *24347* | *P16890* | *Similar to late histone H2B family* | ***S*** | *313* | *3* | *33* | *28%* | *1* | *1.5* |  |
| 13825 |  | Hypothetical protein; domain: CLECT | **T,S** | 574 | 5 | 279 | 18% | 6-13 | 1.4 | P, ↓ |
| 04136 | Q8MUL4 | P19 |  | 356 | 4 | 9 | 30% | 1-3 | 1.2 |  |
| 18810 | Q8MUL1 | Spicule matrix protein SM32 | **T,S** | 348 | 4 | 217 | 15% | 3,6-12 | 1.2 | P, ↓ |
| 23052 |  | Hypothetical protein LOC753888 | **T,S** | 416 | 4 | 11 | 30% | 9,10 | 1.2 | P |
| 15848 |  | Hypothetical protein LOC764009 |  | 335 | 4 | 13 | 35% | 8-10 | 1.2 | P |
| 13670 |  | Similar to matrix metalloproteinase 14/Sp-MT-MMP-e | **T,S** | 1363 | 12 | 262 | 21% | 6-12 | 1.2 | P, ↓ |
| *02788* |  | *Hypothetical protein LOC586940; domain: β-tubulin; peptide set partially matches many similar entries* | ***T,S*** | *1026* | *11* | *35* | *30%* | *1,7* | *1.1* |  |
| 05991 |  | Similar to spicule matrix protein SM29 | **S** | 483 | 5 | 74 | 26% | 8-13 | 1.1 | P, ↓ |
| 05989 |  | Similar to spicule matrix protein SM29 | **T,S** | 510 | 5 | 22 | 29% | 10-12 | 1.1 | P |
| 12518 |  | Similar to carbonic anhydrase | **T,S** | 737 | 7 | 170 | 14% | 6-13 | 1.0 | P, ↓ |
| 17590 |  | Hypothetical protein; peptide set partially overlapping with entry Glean3_22278; domains: Kazal, AP- and G-rich motifs |  | 749 | 7 | 449 | 20% | 1-5 | 1.0 | P |
| 28749 |  | Similar to vertebrate metalloproteinase MMP-24/Sp-MT-MMP-b | **T,S** | 955 | 9 | 224 | 17% | 6-12 | 0.9 | P, ↓ |
| 17587 |  | Hypothetical protein; AP- and G-rich motifs |  | 565 | 6 | 155 | 12% | 1-6 | 0.9 | P |
| 06387  13823 |  | Similar to MSP130/MSP130-related-3 | **T,S** | 1796 | 20 | 327 | 22% | 3-12 | 0.9 | P, ↓ |
| 18964 |  | Similar to MGC53657 protein; domain:FKBP_C |  | 370 | 3 | 22 | 22% | 10-12 | 0.8 | P |
| 17737 |  | Similar to ECM (extracellular matrix protein) 18 |  | 180 | 2 | 6 | 25% | 12 | 0.8 | P, ↑ |
| 04746 |  | Similar to *P. lividus* FGF receptor 2 | **T** | 675 | 8 | 31 | 23% | 6-8,11 | 0.8 | P |
| *03825* |  | *Similar to 1433_CANAL 14-3-3 protein homolog* |  | *310* | *4* | *7* | *13%* | *1,7* | *0.7* |  |
| 07484 | Q45UE8 | Cyclophilin 1 |  | 519 | 4 | 10 | 27% | 8,9,11 | 0.7 | P |
| 27236 |  | Similar to voltage-dependent anion channel 2 isoform 1; domain: Porin_3; N-term: N-acetylated Ala2 | **S** | 582 | 5 | 10 | 19% | 7-9 | 0.7 | P |
| *04721* |  | *Similar to ubiquitin/ribosomal protein S27a; all peptides in ubiquitin domain (aa1-~76)* | ***T,S*** | *389* | *3* | *40* | *45%* | *1-7,10,12* | *0.7* | *P* |
| 21260 |  | Hypothetical protein; domains: 3 LDLR | **T,S** | 374 | 4 | 24 | 16% | 6,7 | 0.6 | P |
| 27172 |  | Hypothetical protein LOC585657; domain: partial esterase_lipase superfamily |  | 537 | 5 | 13 | 19% | 8-10 | 0.6 | P |
| 26000 |  | Hypothetical protein; domain: IG | **T,S** | 805 | 10 | 37 | 17% | 6-10 | 0.6 | P |
| 00475  25962 |  | Similar to MGC139263 protein; domain: annexin |  | 200 | 2 | 3 | 15% | 1,4,7 | 0.5 | P, ↑ |
| 11223 | P07796 | Late histone H1-gamma |  | 210 | 2 | 6 | 13% | 1,2,12 | 0.5 |  |
| 25722 |  | Hypothetical protein/Sp-CA-12-like B; domain: partial carbonic anhydrase (alpha_CA) |  | 162 | 2 | 3 | 11% | 7,9 | 0.5 | P |
| 02219 | Q9TX29 | Nuclear intermediate filament protein |  | 690 | 8 | 10 | 15% | 1 | 0.5 |  |
| 06103 |  | Hypothetical protein LOC756971/similar to calsyntenin-1 |  | 184 | 2 | 10 | 9% | 4,6,7 | 0.5 | P |
| 24800 |  | Hypothetical protein |  | 131 | 1 | 3 | 17% | 12 | 0.5 |  |
| *28221* |  | *Similar to α-tubulin* | ***S*** | *554* | *5* | *17* | *13%* | *1,3,5-7,9* | *0.5* | *(P), ↑* |
| 23016  01796  18054 |  | Similar to extracellular matrix protein 3 (ECM3) of *L.variegatus;* N-terminus  Similar to ECM3*;* C-terminus  Similar to ECM3*;* joining N- and C-terminus | **S**  **S** | 1978  1569  101 | 20  16  1 | 136  122  2 | 14%  13%  3% | 1-8  1-4  3 | 0.4  0.5  0.1 | P  P  P, ↓ |
| 10644 |  | Hypothetical protein; domain: Galactosyl_Transferase |  | 473 | 5 | 30 | 18% | 8-12 | 0.4 | P |
| 06812 |  | Hypothetical protein LOC579471; domains: IG, 2 IGcam | **T** | 794 | 8 | 95 | 9% | 6-12 | 0.4 | P |
| 22278 |  | Hypothetical protein; peptide set overlapping with entry Glean3_17590; AP- and G-rich motifs |  | 361 | 4 | 32 | 4% | 1-5 | 0.4 | P |
| 13669 |  | Similar to metalloproteinase/Sp-MT-MMP-d | **T,S** | 721 | 6 | 74 | 14% | 6-13 | 0.4 | P, ↓ |
| 11562 |  | Similar to phospholipase A2; domain: PLA2c |  | 265 | 2 | 5 | 19% | 10 | 0.4 | P |
| 13756 |  | Similar to peptidyl prolyl isomerase B; domain: cyclophilin_ABH_like |  | 258 | 3 | 7 | 9% | 8,9,11 | 0.4 | P |
| 00439 |  | Hypothetical protein LOC575608 | **T** | 572 | 6 | 36 | 12% | 4-7,10,11 | 0.4 | P |
| 25966 |  | Hypothetical protein; domains: IG, FN3, LRR | **T,S** | 1162 | 13 | 70 | 13% | 3,4,6,8-11 | 0.4 | P |
| 28135 | P15870 | Histone H1-delta; N-acetyl (Ala2) | **S** | 153 | 2 | 5 | 12% | 1 | 0.4 |  |
| 19691 |  | Similar to ENSANGP00000009431/similar to flotillin | **S** | 362 | 5 | 8 | 11% | 1 | 0.4 |  |
| *02503*  *00465* | *Q27866*  *P69141* | *Histone sequences; contains: Histone H3, partial (aa1-90) Histone H2A (aa382-492); H3 and H2A peptide subsets match numerous other Glean3 entries coding for histone sequences* | ***T,S*** | *424* | *5* | *74* | *18%*  *35%* | *1,2,6,-8,11* | *0.4* | *(P)* |
| 03918 |  | Similar to vitellogenin receptor | **S** | 532 | 4 | 9 | 14% | 4,5 | 0.4 | P, ↓ |
| *14869* |  | *Similar to thioredoxin peroxidase* | ***T*** | *141* | *2* | *7* | *9%* | *1,7,9,10* | *0.4* | *P, ↑* |
| 03536 |  | Similar to Os09g0542200; domain: DSBA (thioredoxin) |  | 345 | 3 | 5 | 13% | 9 | 0.3 | P, ↓ |
| 08305 |  | Similar to cyclophilin; domain: cyclophilin_ABH_like | **S** | 227 | 2 | 2 | 9% | 1 | 0.3 |  |
| 15869 |  | Similar to out at first protein |  | 117 | 1 | 1 | 12% | 12 | 0.3 | (P) |
| 13822 | Q8MUK8 | MSP130-related-1 | **T,S** | 409 | 5 | 22 | 8% | 3,4,6-9,12 | 0.3 | P |
| 00438 |  | Similar to peptidylaminoacyl-L/D-isomerase | **T** | 547 | 5 | 19 | 12% | 7-10 | 0.3 | P |
| 17588 |  | Hypothetical protein LOC575627; domains: 2 Kazal, AP-rich and G-rich motifs, V-rich motif |  | 1237 | 13 | 270 | 9% | 1-6 | 0.3 | P |
| *09027*  *00811* | *O16151* | *Similar to RAB35, member of the RAS oncogene family/Rab3 GTPase* |  | *176* | *2* | *3* | *10%* | *7,9* | *0.3* | *(P), ↑* |
| *09165*  *09477*  *14864* |  | *Similar to 71 kDa heat shock cognate protein/similar to HSP70; domain:HSP70; overlapping peptide sets* | ***T*** | *934* | *8* | *14* | *9%* | *1,2,4,7,11* | *0.3* | *P, ↑* |
| 10169 |  | Hypothetical protein; domains: 2 IG | **T** | 253 | 4 | 16 | 12% | 6-8 | 0.3 | P, ↓ |
| 11332 |  | Similar to Ca^2+^-activated chloride channel | **T,S** | 678 | 7 | 41 | 7% | 4,6-8,10,11 | 0.3 | P |
| 13736 |  | Hypothetical protein LOC762462 |  | 139 | 1 | 2 | 12% | 1 | 0.3 |  |
| *00595* | *Q6PTG0* | *Similar to elongation factor 1 alpha* | ***T*** | *282* | *3* | *7* | *8%* | *1,6,7,10* | *0.3* |  |
| 25235 |  | Similar to Egfl6-prov protein; domains: CCP/SUSHI, EGF_Ca, 2 partial vWA_matrilin | **T,S** | 725 | 7 | 54 | 9% | 4-8,10-13 | 0.3 | P, ↓ |
| 25068 |  | Similar to tetraspannin | **T** | 276 | 2 | 14 | 8% | 2-7,11,12 | 0.3 | P, ↑ |
| 10198 |  | Similar to tropomyosin 1 |  | 123 | 1 | 1 | 11% | 6 | 0.3 |  |
| 05538 |  | Hypothetical protein LOC576239; domains: IG | **T,S** | 416 | 4 | 33 | 7% | 6-11 | 0.3 | P |
| 15595 |  | Similar to calponin; domain: CH/SCP1 |  | 394 | 3 | 14 | 8% | 8-11 | 0.3 |  |
| *05061* | *A9LYL5* | *Similar to flotillin* |  | *256* | *3* | *5* | *8%* | *1* | *0.3* |  |
| 04958 |  | Hypothetical protein; domains: partial PX |  | 213 | 2 | 4 | 8% | 6,7 | 0.2 |  |
| 08354  27667 |  | Hypothetical protein; domains: IG, partial IG |  | 398 | 4 | 14 | 6% | 6-8 | 0.2 | P |
| *14564*  *22298*  *22300*  *14567*  *14568*  *14566*  *14565* |  | *Hypothetical protein LOC590213*  *Hypothetical protein LOC584143*  *similar tomechanosensory abnormality protein 2*  *similar tomechanosensory abnormality protein 2*  *similar to Epb7.2-prov protein*  *similar tomechanosensory abnormality protein 2*  *similar tomechanosensory abnormality protein 2; domain: band_7_stomatin_like* |  | *109* | *2* | *6* | *6%* | *1,2,7,8* | *0.2* |  |
| 27906 |  | Hypothetical protein LOC577685/SpC-lectin-PMC1; domain: partial CLECT | **T,S** | 277 | 3 | 69 | 10% | 3-8,11-13 | 0.2 | P, ↓ |
| 14914 |  | Similar to cysteine protease, domains: Cy (cystatin_like), Inhibitor_I29, Peptidase_C1A |  | 327 | 3 | 9 | 7% | 6,8,9 | 0.2 | (P), ↑ |
| 11106  11107 |  | Similar to annexin A4 |  | 186 | 2 | 2 | 5% | 1,7 | 0.2 | (P), ↑ |
| 10589 |  | Hypothetical protein LOC585716; Gly-ich, Pro-rich and Asp-rich motifs; pI~4 |  | 109 | 1 | 13 | 4% | 3,4,6-8 | 0.2 | P |
| 11138 |  | Hypothetical protein; domains: partial SRCR, WSC |  | 220 | 3 | 5 | 8% | 7 | 0.2 | P |
| 20612 |  | Hypothetical protein |  | 223 | 3 | 4 | 10% | 7,8 | 0.2 | P, ↓ |
| 26042 |  | Similar to thrombospondin type I domain containing protein 4/similar to CG6232-PA; domains: TSP_1, PLAC (protease and lacunin) |  | 167 | 2 | 9 | 4% | 5-7 | 0.2 | P |
| 07682 |  | Hypothetical protein LOC757239/Sp-CPE(carboxypeptidase E); domain: peptidase_M14 | **T,S** | 330 | 3 | 4 | 6% | 6-8 | 0.2 | P |
| *10054* |  | *Similar to myosin heavy chain* |  | *1114* | *12* | *21* | *6%* | *1,11,12* | *0.2* | *(P), ↑* |
| 24188 |  | Similar to annexin; domains: 4 annexin |  | 192 | 2 | 2 | 5% | 1 | 0.2 |  |
| 12486 |  | Similar to MEGF11 protein |  | 113 | 1 | 3 | 4% | 9 | 0.2 | P, ↓ |
| 05420 |  | Similar to scavenger receptor cysteine-rich protein type 12; domains: 5 SRCR, 1 CCP |  | 311 | 4 | 8 | 6% | 3,4,7 | 0.2 | P |
| 05709 |  | Similar to 60S ribosomal protein L27A-related |  | 90 | 1 | 1 | 4% | 1 | 0.2 |  |
| 06172 |  | Similar to related to cofilin |  | 120 | 1 | 2 | 5% | 10 | 0.2 | (P) |
| 01874 | B0FLR0 | Advillin |  | 169 | 2 | 2 | 6% | 1 | 0.2 |  |
| 03612 |  | Hypothetical protein LOC752450; domain: ZnMc_astacin_like | **S** | 193 | 2 | 7 | 5% | 8-10 | 0.2 | P |
| 21991 |  | Similar to Carboxypeptidase A1 |  | 100 | 2 | 4 | 5% | 7,9,11,12 | 0.2 |  |
| *21511* |  | *Similar to calmodulin 2* |  | *113* | *2* | *2* | *8%* | *7* | *0.2* |  |
| 25772 | Q27780 | ERcalcistorin/protein disulfide isomerase |  | 173 | 2 | 2 | 5% | 1,7 | 0.2 | P, ↑ |
| 14421 |  | Hypothetical protein |  | 125 | 1 | 1 | 5% | 10 | 0.2 | P |
| 11180 |  | Hypothetical protein; domains: FN3, EGF_CA | **T,S** | 237 | 3 | 15 | 5% | 6,7 | 0.1 | P |
| 18919 |  | Similar to RPGR; domain: partial MDN1; acidic (pI~3.9); contains many short repeats of the type EXSSGEEQPK |  | 208 | 2 | 21 | 4% | 2-5 | 0.1 | P |
| 18702 |  | Hypothetical protein LOC587099; domains: 3 PDI/ERp44 (thioredoxin superfamily) |  | 210 | 2 | 2 | 5% | 7 | 0.1 | P |
| 08613  13077 |  | Similar to TFP250; possibly fragments of one protein |  | 246  214 | 2  2 | 5  4 | 4%  1% | 2,3  9 | 0.1  <0.1 | P  P |
| 05228 |  | Hypothetical protein LOC586019; domains: CA (cadherin), LamG |  | 184 | 2 | 4 | 6% | 3,4,7 | 0.1 | P, ↓ |
| 15537 |  | Hypothetical protein; domain: Arrestin_N |  | 200 | 2 | 2 | 6% | 8,11 | 0.1 |  |
| *12112* |  | *Similar to Rab5 protein* | ***S*** | *117* | *1* | *2* | *5%* | *9* | *0.1* |  |
| 07930 |  | Hypothetical protein/Sp-DNAse-gamma; domain: Exo_endo_phos |  | 105 | 1 | 2 | 3% | 8 | 0.1 | P, ↓ |
| 28748 | Q4G2F5 | Matrix metalloproteinase 16/Sp-MT-MMP-h |  | 164 | 2 | 4 | 3% | 6,8 | 0.1 | P |
| 12548 |  | Similar to secreted protein acidic and rich in cysteine (SPARC, BM-40) |  | 99 | 1 | 3 | 3% | 8 | 0.1 | (P) |
| 18348 |  | Similar to contactin-associated protein 5, partial/ similar to neurexin IV; domains: 1 FA58C, 2 LamG; stretch of Asp at N-term; pI~4.4 |  | 289 | 3 | 8 | 3% | 4,5 | 0.1 | P, ↓ |
| 20457 |  | Hypothetical protein LOC762504; domains: 2 IG | **T** | 174 | 2 | 9 | 30% | 7,9 | 0.1 | P, ↓ |
| *17605* | *Q94760* | *Mitochondrial ATP synthase alpha subunit* |  | *187* | *2* | *2* | *3%* | *1* | *0.1* | *(P)* |
| 08505 |  | Hypothetical protein; domains: 8 cys-rich FGFR |  | 197 | 3 | 6 | 3% | 4,6-8 | 0.1 | P |
| 00469 |  | Similar to cell adhesion molecule OCAM; domains: 5 IG |  | 212 | 2 | 2 | 4% | 3,8 | 0.1 | P, ↓ |
| 06930 |  | Hypothetical protein; domains: 8 EGF_like |  | 111 | 1 | 5 | 3% | 5,6,12 | 0.1 | (P) |
| 14830 |  | Similar to fibropellin Ia; domains: 9 x EGF_Ca, astacin_like |  | 321 | 4 | 19 | 3% | 1,3,5-6,8,9 | 0.1 |  |
| 22057 |  | Similar to MGC68835 protein; domains: Sema, PSI, 3 TSP_1 |  | 304 | 3 | 12 | 3% | 5-7 | 0.1 | P |
| 17586 |  | Similar to hepatopancreas kazal-type proteinase inhibitor; domains: 6 KAZAL |  | 166 | 2 | 6 | 2% | 4,5 | 0.1 | P, ↓ |
| 26094 |  | Similar to cathepsin 1; domains: partial pancreatic_lipase_like, peptidase_C1A |  | 192 | 2 | 11 | 3% | 8-10 | 0.1 | P |
| 08863 |  | Hypothetical protein LOC575414; domain: partial NTR_TIMP |  | 180 | 2 | 5 | 3% | 9-12 | 0.1 | P, ↓ |
| 23855 |  | Similar to MGC81998 protein; domain: Glycosyl_transferase_8 |  | 107 | 1 | 3 | 3% | 7,8 | 0.1 | P, ↓ |
| 04532 |  | Hypothetical protein; partial identity to cyt c oxidase subunit Vic (aa317-358) |  | 138 | 1 | 3 | 2% | 12 | 0.1 |  |
| 27169 |  | Similar to fibulin-6 | **T,S** | 152 | 1 | 2 | 3% | 8 | 0.1 | P, ↓ |
| 28091 |  | Similar to echinonectin |  | 170 | 2 | 3 | 2% | 10-12 | 0.1 | P |
| 24564  24565 |  | Similar to thioester-containing protein; domains: A2M_N, A2M_N_2, A2M, A2M_2, A2M_receptor | **T,S** | 319 | 3 | 5 | 1% | 4,6,7 | 0.1 | I |
| 24083 |  | Hypothetical protein; domain: CH (calponin homology) |  | 143 | 1 | 1 | 2% | 7 | 0.1 |  |
| 04850 |  | Similar to Synaptotagmin IX; domains:  partial CLCA_N, vWFA |  | 243 | 2 | 5 | 2% | 8,9 | 0.1 | P, ↓ |
| 00630 |  | Similar to FAM20C2; domain: DUF1193 |  | 98 | 1 | 1 | 2% | 9 | 0.1 |  |
| *09155* |  | *β-catenin* |  | *113* | *2* | *2* | *2%* | *5,7* | *0.1* |  |
| 05385 |  | Similar to membrane-type matrix metalloproteinase 1 alpha/Sp-MMP-f | **T** | 92 | 1 | 1 | 2% | 11 | 0.1 | P, ↓ |
| 05691  15382 |  | Similar to Grp58-prov protein¸domains: 4 PDI |  | 92 | 1 | 2 | 2% | 6 | 0.1 | (P) |
| 26146 |  | Hypothetical protein; domain: PLA2c |  | 147 | 1 | 1 | 2% | 11 | 0.1 | P |
| 16016 |  | Hypothetical protein;domains: 9 EGF_Ca |  | 134 | 2 | 3 | 2% | 2,3 | 0.1 | P |
| 01892 |  | Hypothetical protein LOC592594, partial/similar to IP13724p, partial; domain: partial semaphorin | T | 84 | 1 | 4 | 1% | 3,4,6 | 0.1 | P, ↓ |
| *20322* |  | *Similar to heat shock 90 kDa protein, partial;*  *domains: HATPase_C, partial HSP90* | *T* | *146* | *1* | *1* | *2%* | *7* | *0.1* |  |
| 05992 |  | Similar to spicule matrix protein SM29 | T | 92 | 1 | 1 | 1% | 4 | 0.1 | P, ↓ |
| 19665  24019 |  | Similar to nephrin/tenascin R |  | 137 | 2 | 2 | <1% | 3 | <0.1 | P |
| 15906 |  | Hypothetical protein LOC587327 |  | 161 | 2 | 4 | 1% | 8 | <0.1 | P, ↓ |
| 25502 |  | Hypothetical protein; domains: 7 IGcam, 4 FN3 |  | 194 | 2 | 5 | 2% | 2-4 | <0.1 | P |
| 13301 | P19615 | Major yolk protein | **T** | 219 | 2 | 5 | 1% | 3,4 | <0.1 |  |
| 26949 |  | Similar to Solute carrier family 34 (sodium phosphate), member 2, partial (aa38-698)  Similar to melanotransferrin/EOS47 (aa699-1419);  peptides in transferring domains | **T** | 172 | 2 | 7 | 1% | 5,8 | <0.1 | P, ↓ |
| 23115 |  | Similar to brain RPTPmam4 isoform II; domains: 12 FN3, 2 PTPc (protein tyrosine phosphatase), 1 EGF_Ca |  | 171 | 2 | 6 | <1% | 8,9 | <0.1 | P |
| 00241 |  | Similar to talin |  | 169 | 2 | 2 | <1% | 1 | <0.1 |  |
| 16052 |  | Similar to apolipophorin |  | 191 | 2 | 4 | <1% | 6,7 | <0.1 | P |
| 15404 |  | Similar to DEAH (Asp-Glu-Ala-His) box polypeptide 33; domains: 3 LamG, 1 partial LamG, 1 EGF_Ca |  | 109 | 1 | 2 | <1% | 4 | <0.1 | P, ↓ |
| 13917 |  | Similar to Bent, partial; domains: 17 FN3, 19 IG, 1 Thr/Ser protein kinase |  | 112 | 1 | 1 | <1% | 11 | <0.1 |  |

Proteins are ordered according to decreasing emPAI. The average absolute mass accuracy was 0.67 ppm (p<0.05). Mascot protein scores were calculated with MSQuant from unique peptide scores including MS^3^ scores. If the protein was identified in more than three gel sections only sections containing more than 5% of the total peptide number are indicated. S, also identified in spines; T, also identified in test [29]. Proteins sharing peptides with human entries are shown in *italics*. P, also in powdered tooth matrix; (P), tentatively identified in powdered tooth matrix. ↑, emPAI at least doubled compared to powdered tooth matrix; ↓, emPAI at least halved compared to powdered tooth matrix.
