# Supplementary material for: In-depth, high-accuracy proteomics of sea urchin tooth organic matrix
Source: Proteome Sci. 2008 Dec 9;6:33. doi: 10.1186/1477-5956-6-33 (PMC2614417; doi:10.1186/1477-5956-6-33)
Supplement: Additional file 5 — Proteins tentatively identified in the matrix of intact teeth. Identifications with a single unique peptide showing good quality, manually validated, spectra without MS3 confirmation. [file 1477-5956-6-33-S5.doc]

**Proteins tentatively identified in the matrix of intact teeth**

|  |  |  |  |  |  |  |  |  |  |  |
| --- | --- | --- | --- | --- | --- | --- | --- | --- | --- | --- |
| **GLEAN3**  **accession** | **Swiss-Prot/**  **Trembl**  **accession** | **Protein** |  | **Protein score** | **Unique pep-tides** | **Total**  **accep-ted**  **pep-tides** | **Sequ-ence**  **cover-age** | **Gel**  **section** | **emPAI** |  |
|  |  |  |  |  |  |  |  |  |  |  |
| 11163 |  | Hypothetical protein LOC588710; domain: CLECT |  | 109 | 1 | 18 | 8% | 9-11 | 0.6 | (P) |
| 28880 |  | Hypothetical protein LOC586809;domain: ATP synthase E chain, partial |  | 79 | 1 | 1 | 16% | 12 | 0.5 |  |
| 00164 |  | Similar to SM30 |  | 108 | 1 | 16 | 16% | 3-12 | 0.4 | (P) |
| 28887 |  | Similar to cytoplasmic cystatin |  | 136 | 1 | 6 | 12% | 11 | 0.3 | P, ↓ |
| 14792 |  | Hypothetical protein LOC590042 |  | 46 | 1 | 1 | 7% | 11 | 0.3 |  |
| 03161 |  | Hypothetical protein |  | 55 | 1 | 1 | 14% | 10 | 0.3 |  |
| 09352 |  | Hypothetical protein LOC585121 |  | 107 | 1 | 17 | 7% | 1,2,7-10,13 | 0.2 |  |
| 04876 |  | Similar to ferritin | **S** | 91 | 1 | 1 | 7% | 9 | 0.2 |  |
| 14715 |  | Similar to gelsolin | **S** | 95 | 1 | 2 | 5% | 1 | 0.2 |  |
| *07485*  *07553*  *13414*  *27618* | *Q6QM15* | *Guanine nucleotide-binding protein G(s) alpha subunit* |  | *111* | *1* | *1* | *2%* | *1* | *0.2* |  |
| 03718 |  | Similar to Ribosomal protein S16 |  | 45 | 1 | 1 | 5% | 1 | 0.2 |  |
| 11256 |  | Similar to phospholipase A2 |  | 66 | 1 | 3 | 5% | 10,11 | 0.2 |  |
| 21630  26629  26630 |  | Hypothetical protein LOC582612/similar to fibulin | **T** | 69 | 1 | 1 | 5% | 3,4 | 0.2 |  |
| 15335 | Q9Y0S8 | Vesicle-associated membrane protein |  | 91 | 1 | 1 | 6% | 11 | 0.2 |  |
| 04584 |  | Similar to MGC80358 protein; domains: 3 MIR |  | 57 | 1 | 1 | 7% | 9 | 0.1 |  |
| 26306 |  | Hypothetical protein LOC583480/similar to tetraspanin |  | 78 | 1 | 1 | 4% | 12 | 0.1 |  |
| *23217* |  | *Similar to GTP-binding nuclear protein Ran* | ***T*** | *87* | *1* | *1* | *4%* | *7* | *0.1* |  |
| 00128 |  | Similar to tropomyosin |  | 89 | 1 | 5 | 4% | 3,4,6,7 | 0.1 |  |
| 22047 |  | Similar to phospholipase A2 |  | 81 | 1 | 2 | 3% | 7 | 0.1 |  |
| 06306 |  | Hypothetical protein; domains: 2 Glect |  | 88 | 1 | 1 | 3% | 7 | 0.1 |  |
| 15323 |  | Similar to arginine kinase |  | 111 | 1 | 2 | 3% | 1 | 0.1 |  |
| 13893 |  | Similar to LOC494800 protein; domain: peptidase_C1A_cathepsinX |  | 44 | 1 | 1 | 3% | 8 | 0.1 | (P), ↓ |
| 15856 |  | Hypothetical protein LOC584467; domain: Lamp (lysosome membrane-associated protein |  | 40 | 1 | 1 | 2% | 3 | 0.1 |  |
| 04230 | Q5QIB9 | Phospholipase C delta isoform |  | 67 | 1 | 1 | 1% | 1 | 0.1 |  |
| 03084 |  | Glypican-6 |  | 64 | 1 | 1 | 1% | 10 | 0.1 | (P) |
| 11065 | P92163 | Integrin beta G subunit |  | 92 | 1 | 1 | 1% | 3 | 0.1 | (P), ↓ |
| 03540 |  | Similar to prominin |  | 107 | 1 | 1 | 1% | 5 | 0.1 | P |
| 11293 |  | Similar to HrES-AP (alkaline phosphatase) |  | 80 | 1 | 1 | 1% | 8 | 0.1 | P, ↓ |
| 17159 |  | Similar to chondroitin beta1,4 N-acetylgalactosaminyltransferase |  | 71 | 1 | 1 | 1% | 10 | 0.1 |  |
| 02117 |  | Similar to leishmanolysin-like (metallopeptidase M8 family) |  | 80 | 1 | 1 | 1% | 8 | <0.1 | (P) |
| 10564 |  | Hypothetical protein; domains: 6 LRRCT |  | 60 | 1 | 1 | 1% | 8 | <0.1 |  |
| 18452 |  | Hypothetical protein LOC582531; domain: partial semaphorin |  | 130 | 1 | 1 | 2% | 10 | <0.1 | P |
| 01129 |  | Hypothetical protein LOC763187; domains:LRR_RI, IG |  | 48 | 1 | 1 | 1% | 7 | <0.1 |  |
| 00881  07085 |  | Major Vault Protein |  | 73 | 1 | 2 | 1% | 1 | <0.1 |  |
| 09922 |  | Hypothetical protein; domains: 3 cys_rich_FGFR |  | 78 | 1 | 5 | <1% | 7,8 | <0.1 | P |
| 11588 |  | Hypothetical protein LOC582410/similar to 2 alpha fibrillar collagen (no triple helical sequence) | **S** | 77 | 1 | 4 | <1% | 8,9 | <0.1 | P |
| 16807 |  | Similar to neurogenic locus notch (notch)/similar to fibropellin Ia |  | 85 | 1 | 1 | <1% | 3 | <0.1 |  |

S, also in spines; T, also in test [29]. The average absolute mass accuracy was 0.67 ppm (p<0.05). Proteins sharing their unique peptide with human proteins are shown in *italics*. P, proteins also identified in powdered tooth matrix; (P), tentatively identified in powdered tooth matrix. ↑, emPAI at least doubled compared to powdered tooth matrix; ↓, emPAI at least halved compared to powdered tooth matrix.
