# Supplementary material for: In-depth, high-accuracy proteomics of sea urchin tooth organic matrix
Source: Proteome Sci. 2008 Dec 9;6:33. doi: 10.1186/1477-5956-6-33 (PMC2614417; doi:10.1186/1477-5956-6-33)
Supplement: Additional file 6 — Sequences of unique peptides identified in intact tooth matrix. List of sequences of accepted peptides from intact tooth matrix. [file 1477-5956-6-33-S6.doc]

**Sequences of unique peptides identified in intact tooth matrix**

| **Glean3_entry** | **Sequence** |
| --- | --- |
|  |  |
| *GLEAN3_00128* | *LAQLTDEITTLR* |
| *GLEAN3_00164* | *MSSGESYTATGAR* |
| GLEAN3_00241 | AIAAATSALVK |
| GLEAN3_00241 | VGETQNALLR |
| GLEAN3_00438 | APMDLTGTYITADKPVAVVSGATCTR |
| GLEAN3_00438 | FMFAMPSNFNVASK |
| GLEAN3_00438 | TNDAFVAIPTDALGK |
| GLEAN3_00438 | VMVNIPGFK |
| GLEAN3_00438 | VTLMIGSSNPGIAR |
| GLEAN3_00439 | GQTLTVNVGK |
| GLEAN3_00439 | LSLLIGANEPGLTR |
| GLEAN3_00439 | NILTNYEK |
| GLEAN3_00439 | NVDINPDVAAR |
| GLEAN3_00439 | SGGFIFR |
| GLEAN3_00439 | TFTADATVPLDDVR |
| GLEAN3_00469 | SDSLIINQISK |
| GLEAN3_00469 | TPIQSQCTPDQLR |
| GLEAN3_00475 | GAGTDDDTLIR |
| GLEAN3_00475 | SEIDLVEIK |
| GLEAN3_00595 | IGGIGTVPVGR (EF1 alpha-2) |
| GLEAN3_00595 | LPLQDVYK (EF1 alpha-2) |
| GLEAN3_00595 | STTTGHLIYK (EF1 alpha-2) |
| GLEAN3_00630 | GFVVALDNGK |
| GLEAN3_00685 | DNIQGITKPAIR (histone H4) |
| GLEAN3_00685 | ISGLIYEETR (histone H4) |
| GLEAN3_00685 | TVTAMDVVYALK (histone H4) |
| GLEAN3_00685 | VFLENVIR (histone H4) |
| *GLEAN3_00881* | *IEGEAAVDQAR* |
| *GLEAN3_01129* | *ITQLTANTFR* |
| GLEAN3_01796 | AIGSEGDAGR |
| GLEAN3_01796 | ASNPVSAIAGEDYAGISR |
| GLEAN3_01796 | GYPLVCVSPCNPK |
| GLEAN3_01796 | HPTYEVNENDIR |
| GLEAN3_01796 | LTVTMPHVDGMLPIISTR |
| GLEAN3_01796 | NLDFAPGVNQQTVK |
| GLEAN3_01796 | NPNVVGDTYAYQYSPELR |
| GLEAN3_01796 | QADSDTFFSTTK |
| GLEAN3_01796 | QLSNFELALSK |
| GLEAN3_01796 | SITLDSIYFGPGSR |
| GLEAN3_01796 | TGDLSIESSVR |
| GLEAN3_01796 | VANAIGAEPFTAR |
| GLEAN3_01796 | VDENTPFIEVTVFR |
| GLEAN3_01796 | VFLCTGR |
| GLEAN3_01796 | VYIIDDR |
| GLEAN3_01796 | YTGPADPDYPNK |
| GLEAN3_01874 | DPDSTQLIQVK |
| GLEAN3_01874 | FTLAELQAK |
| GLEAN3_01892 | LLDVDSDR |
| *GLEAN3_02117* | *QSLSTVLLNR* |
| GLEAN3_02219 | AQNNALIAR |
| GLEAN3_02219 | EQTSEQTTLLR |
| GLEAN3_02219 | LATYIDR |
| GLEAN3_02219 | NAAVNSQDEVR |
| GLEAN3_02219 | QEQDDHLEATTLR |
| GLEAN3_02219 | QVEEETLLR |
| GLEAN3_02219 | STVDDLTSEITTIR |
| GLEAN3_02219 | VASLESQIAEK |
| GLEAN3_02503 | AGLQFPVGR (histone H2A) |
| GLEAN3_02503 | HLQLAVR (histone H2A) |
| GLEAN3_02503 | LLGGVTIAQGGVLPNIQAVLLPK (histone H2A) |
| GLEAN3_02503 | STELLIR (histone H3) |
| GLEAN3_02503 | YRPGTVALR (histone H3) |
| GLEAN3_02788 | AVLVDLEPGTMDSVR (β-tubulin) |
| GLEAN3_02788 | EIVHLQAGQCGNQIGAK (β-tubulin) |
| GLEAN3_02788 | EVDEQMLNVQNK (β-tubulin) |
| GLEAN3_02788 | FPGQLNADLR (β-tubulin) |
| GLEAN3_02788 | IMNTFSVVPSPK (β-tubulin) |
| GLEAN3_02788 | INVYYNEATGGK (β-tubulin) |
| GLEAN3_02788 | ISEQFTAMFR (β-tubulin) |
| GLEAN3_02788 | LAVNMVPFPR (β-tubulin) |
| GLEAN3_02788 | MSATFIGNSTAIQELFK (β-tubulin) |
| GLEAN3_02788 | NSSYFVEWIPNNVK (β-tubulin) |
| GLEAN3_02788 | YLTVAAIFR (β-tubulin) |
| *GLEAN3_03084* | *TFGQGLGFGR* |
| *GLEAN3_03161* | *LQEVEAIGGQEHPLEPEEYVVSK* |
| GLEAN3_03536 | QAALSDANISEQPDSYILK |
| GLEAN3_03536 | QVIDPLLAK |
| GLEAN3_03536 | VADYYGPDTVR |
| *GLEAN3_03540* | *VYETESFLR* |
| GLEAN3_03612 | DQSLYDQSLLK |
| GLEAN3_03612 | ICTSFLGR |
| *GLEAN3_03718* | *IAQIYAIR* |
| GLEAN3_03825 | DSTLIMQLLR (14-3-3 theta) |
| GLEAN3_03825 | LAEQAER (14-3-3 theta) |
| GLEAN3_03825 | NLLSVAYK (14-3-3 theta) |
| GLEAN3_03825 | VNEELSVEER |
| GLEAN3_03918 | ANPEFGAPK |
| GLEAN3_03918 | CDCDENYTLENVNDR |
| GLEAN3_03918 | IFDPSTSSEDYIK |
| GLEAN3_03918 | TQLQAENEVVPVVVTDK |
| GLEAN3_04136 | GASIGAEPANSSEER |
| GLEAN3_04136 | IEEGQASGEGAGEEGK |
| GLEAN3_04136 | LEAAPEAPPK |
| GLEAN3_04136 | QPTEAEMDAELQK |
| *GLEAN3_04230* | *VPELALIR* |
| GLEAN3_04532 | TYDAQADFER |
| *GLEAN3_04584* | *AVEGIFVKPSEGSTVS* |
| GLEAN3_04721 | ESTLHLVLR (ubiquitin) |
| GLEAN3_04721 | TITLEVEPSDSIENVK |
| GLEAN3_04721 | TLSDYNIQK (ubiquitin) |
| GLEAN3_04746 | ADPPPVPR |
| GLEAN3_04746 | HAPFLQTDR |
| GLEAN3_04746 | IGAPAIAYMTPTIATTIGSTVR |
| GLEAN3_04746 | NSLAVISKPMSLNVK |
| GLEAN3_04746 | QYQDGVSTLSITPQGR |
| GLEAN3_04746 | SDAHVYAYHSR |
| GLEAN3_04746 | SNPGQLLPSGR |
| GLEAN3_04746 | VSVGPVLSVTVSSISDGGR |
| GLEAN3_04850 | FISLTPGYISNR |
| GLEAN3_04850 | STDPEFIIVR |
| GLEAN3_04867 | IDPVVHDPNR |
| GLEAN3_04867 | MMVEPVPVWIGLHVGPMGR |
| GLEAN3_04867 | QPGFGNPGTPGGR |
| GLEAN3_04867 | QPGQPGVGGQPGVGGR |
| GLEAN3_04867 | QPGVGGQPGFGNPGTPGGR |
| GLEAN3_04867 | QPGVGGQPGVGGR |
| GLEAN3_04867 | QPGWGQPGVGQPGTPGGR |
| *GLEAN3_04876* | *GGSIVFQDIK* |
| GLEAN3_04958 | TGGEAYLLGGGPK |
| GLEAN3_04958 | YEEAFIEK |
| GLEAN3_05061 | DADIGVAEAER (flotillin-2) |
| GLEAN3_05061 | LAAEISAPLSK |
| GLEAN3_05061 | TSEIVLLGDDR |
| GLEAN3_05228 | EGLSFNVGNLPVSVAR |
| GLEAN3_05228 | QETGDGFYPSMYR |
| GLEAN3_05385 | GQVDLGFPR |
| GLEAN3_05420 | EITDCQSDANVICK |
| GLEAN3_05420 | HSLEGNVEISK |
| GLEAN3_05420 | SDANVLCR |
| GLEAN3_05420 | SSQDTGVTDLR |
| GLEAN3_05538 | IESISASTFSGAAK |
| GLEAN3_05538 | LETLDLSR |
| GLEAN3_05538 | LLVPQQAGAGLLAVR |
| GLEAN3_05538 | LTNLLQDAFR |
| GLEAN3_05691 | ESAGIISFMK |
| GLEAN3_05709 | APVIDAVR |
| GLEAN3_05989 | FQHNFLTFTGGANNK |
| GLEAN3_05989 | GYLITAK |
| GLEAN3_05989 | LPFMEAQMQCLSFR |
| GLEAN3_05989 | MPGQAVYPLQGCQPGWTNFGK |
| GLEAN3_05989 | VWMGLAER |
| GLEAN3_05990 | FKPDQPQQNAHR |
| GLEAN3_05990 | FQHNWLR |
| GLEAN3_05990 | FSAQAVPGQRPGFGMPPR |
| GLEAN3_05990 | GHLIVTK |
| GLEAN3_05990 | IWMGLAELPSAPESNR |
| GLEAN3_05990 | LPYDEANMFCAR |
| GLEAN3_05991 | GYLMTSK |
| GLEAN3_05991 | MNWLQAQR |
| GLEAN3_05991 | TEMSFICQYQYML |
| GLEAN3_05991 | VWLGLSEK |
| GLEAN3_05991 | YSALNGTTPR |
| GLEAN3_05992 | TPMSYVCK |
| GLEAN3_06103 | FVSNDFDVR |
| GLEAN3_06103 | QISYSQVASGK |
| GLEAN3_06172 | FQYAVFQFK |
| *GLEAN3_06306* | *IDTIAIDGK* |
| GLEAN3_06387 | DQNPQGQNPNGQSPQGGVTTSR |
| GLEAN3_06387 | GGTGWNNQGPNQGGR |
| GLEAN3_06387 | GGTGWNNQGPQGGGAR |
| GLEAN3_06387 | VPNQGPNQGGR |
| GLEAN3_06812 | CVYGNQFISAR |
| GLEAN3_06812 | GTGETNVTGYR |
| GLEAN3_06812 | HGETAQFTCR |
| GLEAN3_06812 | HGPSDTVLVQGK |
| GLEAN3_06812 | KGEYYLTIR |
| GLEAN3_06812 | LIAGPTVHANSVQR |
| GLEAN3_06812 | NPPMVTVTPPEVNIK |
| GLEAN3_06812 | TCTVMVLK |
| GLEAN3_06930 | IGGETTCYCPR |
| GLEAN3_07484 | LGITMPVVGSTGDF |
| GLEAN3_07484 | NFMIQGGDFASEDGSGSR |
| GLEAN3_07484 | TEDLPPQDEFIVEK |
| GLEAN3_07484 | TVANFLFFADPLSK |
| *GLEAN3_07485* | *LLLLGAGESGK (Guanine-bindig protein G-α)* |
| GLEAN3_07682 | HLAQTYATK |
| GLEAN3_07682 | SFYTASPDDAVFK |
| GLEAN3_07682 | VYTIGTSVK |
| GLEAN3_07930 | YDMVLIQEIR |
| GLEAN3_08305 | FADENFTLK |
| GLEAN3_08305 | VAQGSDVIK |
| GLEAN3_08354 | FEGIQFGTGK |
| GLEAN3_08354 | LNPTSSFLTSGTQSFSSEEGK |
| GLEAN3_08354 | SMSTVDEGQEVR |
| GLEAN3_08354 | TRFEGIQFGTGK |
| GLEAN3_08505 | AVSVHLNPR |
| GLEAN3_08505 | LCPTVTQGNAR |
| GLEAN3_08505 | VAELSSNDYHEDR |
| GLEAN3_08613 | GLVLDAVDIK |
| GLEAN3_08613 | SPPSVIR |
| GLEAN3_08863 | STVNVFTEPEESMCGK |
| GLEAN3_08863 | VYKGEEMISVK |
| GLEAN3_09027 | LLIIGDSGVGK (Rab-1A) |
| GLEAN3_09027 | LQIWDTAGQER (Rab-1A) |
| GLEAN3_09155 | LLWTTSR (β-catenin) |
| GLEAN3_09155 | LVQNCLWTLR (β-catenin) |
| GLEAN3_09165 | GTLEPVENALR |
| GLEAN3_09165 | IINEPTAAAIAYGLDK (HSP70) |
| GLEAN3_09165 | LLQDFFNGK (HSP70) |
| GLEAN3_09165 | TTPSYVAFTDTER (HSP70) |
| GLEAN3_09165 | VEIIANDQGNR (HSP70) |
| *GLEAN3_09352* | *TATNLQQTSR* |
| GLEAN3_09477 | DAGVIAGLNVLR (HSP70) |
| GLEAN3_09481 | AGFAGDDAPR (Actin) |
| GLEAN3_09481 | HQGVMVGMGQK (Actin) |
| GLEAN3_09481 | DSYVGDEAQSK (Actin) |
| GLEAN3_09481 | VAPEEHPVLLTEAPLNPK (Actin) |
| GLEAN3_09481 | DLTDYLMK (Actin) |
| GLEAN3_09481 | GYSFTTTAER (Actin) |
| GLEAN3_09481 | SYELPDGQVITIGNER (Actin) |
| GLEAN3_09481 | IIAPPER (Actin) |
| GLEAN3_09481 | QEYDESGPSIVHR (Actin) |
| *GLEAN3_09922* | *FDNAAAEVCR* |
| GLEAN3_10054 | ALPLEENEFR |
| GLEAN3_10054 | AQLEITQVK |
| GLEAN3_10054 | DLEDYEER |
| GLEAN3_10054 | DQLQITER |
| GLEAN3_10054 | ELQATVDEETR |
| GLEAN3_10054 | IEELEEELEAER (myosin-4) |
| GLEAN3_10054 | LAEGDLQDANDR |
| GLEAN3_10054 | LDEAEAAALK |
| GLEAN3_10054 | LGLGVIQR |
| GLEAN3_10054 | LGSENSDLAR |
| GLEAN3_10054 | QLEESEENVNQNLAK |
| GLEAN3_10054 | VELAETQVNK |
| GLEAN3_10169 | ATFTCALMSTNNIGVR |
| GLEAN3_10169 | GEEELEENEGLR |
| GLEAN3_10169 | QILRPQDTNIQYTCLMR |
| GLEAN3_10169 | VLEIQDIR |
| GLEAN3_10198 | LSETQAAFDEAEK |
| *GLEAN3_10564* | *DFDGLQSLR* |
| GLEAN3_10589 | VIEKPIYIPVSVPR |
| GLEAN3_10644 | FLTPESLYPDK |
| GLEAN3_10644 | FTLGTVLFATPVR |
| GLEAN3_10644 | IVFLLGAVR |
| GLEAN3_10644 | TELAENDNAIDEIR |
| GLEAN3_10644 | VDDDIIINAPLVYNILQTASPTK |
| *GLEAN3_11065* | *YLGELCGGSDR* |
| GLEAN3_11106 | ALDALLS |
| GLEAN3_11106 | GLGTDEAVLVR |
| GLEAN3_11138 | FTDPALTGAVVVEQSMTLSR |
| GLEAN3_11138 | QLNYPGANR |
| GLEAN3_11138 | YPAGDYPMSEFAFR |
| *GLEAN3_11163* | *QQVTDILNFNQMAR* |
| GLEAN3_11180 | GPIVVEPVGPR |
| GLEAN3_11180 | GVCTNDPFTGFK |
| GLEAN3_11180 | VQPGAGPGNNPNTGR |
| GLEAN3_11223 | GVASGALVQTK |
| GLEAN3_11223 | YIAANYTVDMTK |
| *GLEAN3_11256* | *TAALCFAR* |
| *GLEAN3_11293* | *AAVEEALLR* |
| GLEAN3_11332 | ANLMSVVSR |
| GLEAN3_11332 | DVAILIPK |
| GLEAN3_11332 | FISESPEDSQVGIASYSNAGR |
| GLEAN3_11332 | LLALDDVGNR |
| GLEAN3_11332 | NPSGHVYTLR |
| GLEAN3_11332 | STTVAVVDTSR |
| GLEAN3_11332 | SYLQDMTPEGK |
| GLEAN3_11562 | EGVCAGDTFGQSYGYK |
| GLEAN3_11562 | NGPTDGIVPVDQIDK |
| *GLEAN3_11588* | *LGSSFAFGK* |
| GLEAN3_12112 | LVLLGESAVGK (Rab-5C) |
| GLEAN3_12486 | GDSAHVGDVYVIK |
| GLEAN3_12518 | DMLPTDLSCFYR |
| GLEAN3_12518 | KQSPINIESR |
| GLEAN3_12518 | QSPINIESR |
| GLEAN3_12518 | TIAEAVK |
| GLEAN3_12518 | VEVSNDGHTLK |
| GLEAN3_12518 | VEYYAHLPLR |
| GLEAN3_12518 | VSTEGMYVLK |
| GLEAN3_12548 | ITLVDNALR |
| GLEAN3_13077 | NPTSDVITLYK |
| GLEAN3_13077 | YAEAVFNFK |
| GLEAN3_13301 | GQVTFVDQK |
| GLEAN3_13301 | IGSVFESVNR |
| GLEAN3_13669 | DNEVYFVTPK |
| GLEAN3_13669 | GNDAVSNALQTESVFVDMQDIPDAAFIR |
| GLEAN3_13669 | IWEFSQGVYQPR |
| GLEAN3_13669 | RGNDAVSNALQTESVFVDMQDIPDAAFIR |
| GLEAN3_13669 | TNKIDAAFK |
| GLEAN3_13669 | TSYFLIGGQVSTFK |
| GLEAN3_13670 | ADIIVQFAR |
| GLEAN3_13670 | DNEVFFIAETR |
| GLEAN3_13670 | FTLNTDDVR |
| GLEAN3_13670 | GGVSYFLIDK |
| GLEAN3_13670 | GIQSLYGAR |
| GLEAN3_13670 | GTEPVSNALR |
| GLEAN3_13670 | KGTEPVSNALR |
| GLEAN3_13670 | LDFNPTR |
| GLEAN3_13670 | NGPINAAWTEGK |
| GLEAN3_13670 | SLMAPYYQGFQPR |
| GLEAN3_13670 | TEAVFTEMSAVPDAAFIR |
| GLEAN3_13670 | TNGIDAAFK |
| GLEAN3_13736 | EIVEGQMVTTVSK |
| GLEAN3_13756 | DFMIQGGDFTK |
| GLEAN3_13756 | NFIGLATGEK |
| GLEAN3_13756 | VLEGMDVVR |
| GLEAN3_13821 | DGHIDASYNALYK |
| GLEAN3_13821 | ELCSVEVGSQPIAVR |
| GLEAN3_13821 | FNAPAEVQR |
| GLEAN3_13821 | GPECESLAVGDVQGR |
| GLEAN3_13821 | GQTGGMYALNNGVAFK |
| GLEAN3_13821 | IDASSADR |
| GLEAN3_13821 | KTGDLSPESLSFIPPEK |
| GLEAN3_13821 | KYVNPEGTITTVR |
| GLEAN3_13821 | LPVGGGAGGAGGAGGAGGGGGGGGGAGGR |
| GLEAN3_13821 | LYLPFDKLPVGGGAGGAGGAGGAGGGGGGGGGAGGR |
| GLEAN3_13821 | QIATEGPVADIAECGDLVAFTQPGKPHFTDVGSLK |
| GLEAN3_13821 | QLAYVGGGQFVQIVDFSDVVQPK |
| GLEAN3_13821 | QPYTGQLGDPGPHTFSR |
| GLEAN3_13821 | SQTNKPLLLVTGR |
| GLEAN3_13821 | TGDLSPESLSFIPPEK |
| GLEAN3_13821 | YVNPEGTITTVR |
| GLEAN3_13822 | LFNDPALAPLMEQQFIR |
| GLEAN3_13822 | SRPPMVLVTSR |
| GLEAN3_13822 | TDPGMVHIYQK |
| GLEAN3_13822 | VIIVGIK |
| GLEAN3_13822 | VSGTVNLYR |
| GLEAN3_13823 | EFGDSDPESITFIPPEK |
| GLEAN3_13823 | FDSPRPVTDIAECGR |
| GLEAN3_13823 | GTCSGGGNFNPTVTTLDFTK |
| GLEAN3_13823 | HDDVYFYGGR |
| GLEAN3_13823 | LGNLEFSR |
| GLEAN3_13823 | LIFVGIDR |
| GLEAN3_13823 | MGPECESIEIGDVQGTK |
| GLEAN3_13823 | NCEFIVGSRPK |
| GLEAN3_13823 | QPYTGQMGDK |
| GLEAN3_13823 | RYDLDPSPGNAR |
| GLEAN3_13823 | TKEFGDSDPESITFIPPEK |
| GLEAN3_13823 | VGDPSLTMASTFDSQSDK |
| GLEAN3_13823 | VSGTITIYQIR |
| GLEAN3_13823 | VSNAEMLDAFR |
| GLEAN3_13823 | YDIESFR |
| GLEAN3_13823 | YDLDPSPGNAR |
| GLEAN3_13825 | AENALGWR |
| GLEAN3_13825 | IMLSYLTTVFGQGR |
| GLEAN3_13825 | MTFNEATFFCNR |
| GLEAN3_13825 | YGGSLYALDSPSK |
| GLEAN3_13825 | YGGSLYALDSPSKNR |
| *GLEAN3_13893* | *GPMMAEISAR* |
| GLEAN3_13917 | TLLGIEPK |
| GLEAN3_14421 | LELPYIVSNR |
| GLEAN3_14564 | LLAQTTLR (Erythrocyte band 7 protein) |
| GLEAN3_14564 | NLAEILAER (Erythrocyte band 7 protein) |
| *GLEAN3_14715* | *LPDTEVIR* |
| *GLEAN3_14792* | *NVTLGHPTNR* |
| GLEAN3_14830 | IMDAINR |
| GLEAN3_14830 | INSGILSNAR |
| GLEAN3_14830 | NDFSENDIYK |
| GLEAN3_14830 | TNQQNYLHIVPTEGCYSSVGMQGGR |
| GLEAN3_14864 | STAGDTHLGGEDFDNR (HSP70) |
| GLEAN3_14869 | GLFIIDDK (Peroxiredoxin-1) |
| GLEAN3_14869 | LVQAFQFTDK (Peroxiredoxin-1) |
| GLEAN3_14914 | DCQASDIKDEYR |
| GLEAN3_14914 | FADMTEAEFR |
| GLEAN3_14914 | SIPQDVVESALVK |
| GLEAN3_15323 | LGSSEVEQVNR |
| *GLEAN3_15335* | *DQALSVLDDR* |
| GLEAN3_15404 | TCTEDVGATFK |
| GLEAN3_15537 | LGQAVNEYLNTK |
| GLEAN3_15537 | LPFSFLLPEGLPTSFESK |
| GLEAN3_15595 | ANLESFIQGLK |
| GLEAN3_15595 | DQDVFQVNDLYESK |
| GLEAN3_15595 | FAQSQPGYDGPILGPR |
| GLEAN3_15848 | FNSISVTPQPIAMPGTIR |
| GLEAN3_15848 | IAVNVEIVR |
| GLEAN3_15848 | NILQTMSAEITVSR |
| GLEAN3_15848 | SAFTATDCGGR |
| *GLEAN3_15856* | *IQTADIDNNK* |
| GLEAN3_15869 | AEFISSDAMSK |
| GLEAN3_15906 | FEDQNPNQIPIR |
| GLEAN3_15906 | VFPIFPEYSTK |
| GLEAN3_16016 | GYSTTIIEAK |
| GLEAN3_16016 | LVSYANAANR |
| GLEAN3_16052 | GFNDVFVK |
| GLEAN3_16052 | LAANYPFR |
| GLEAN3_16506 | ANNAVVQLILTPGNEAITGFNPMGMK |
| GLEAN3_16506 | APMDGEFSILMDNK |
| GLEAN3_16506 | AQQQQQQGGQPNYPGQGAGAGTR |
| GLEAN3_16506 | FATTQGNCAAQFGHR |
| GLEAN3_16506 | FFHNCETIVIANK |
| GLEAN3_16506 | GTPAADAATNTFTDPEGTISIVR |
| GLEAN3_16506 | GVAETYPQVFNSK |
| GLEAN3_16506 | KAPMDGEFSILMDNK |
| GLEAN3_16506 | KSGTLSMYR |
| GLEAN3_16506 | KWNLLCDAK |
| GLEAN3_16506 | MTVTTIDFR |
| GLEAN3_16506 | NMGDIDPAK |
| GLEAN3_16506 | NPQNPNSGYDYVSFFGGR |
| GLEAN3_16506 | QPNAIESFEIR |
| GLEAN3_16506 | SATPDNYNYLMVMGK |
| GLEAN3_16506 | SFTDAVPFSR |
| GLEAN3_16506 | SGTLSMYR |
| GLEAN3_16506 | VGADPVSLK |
| *GLEAN3_16807* | *NGAICLDGANR* |
| GLEAN3_16845 | AMSIMNSFVNDVFER |
| GLEAN3_16845 | LLLPGELAK (histone H2B) |
| GLEAN3_16845 | QVHPDTGISSR |
| *GLEAN3_17159* | *IITLSGEFSR* |
| GLEAN3_17586 | ANCDGSLVQR |
| GLEAN3_17586 | YACFTNTPK |
| GLEAN3_17587 | APAPPAPVPR |
| GLEAN3_17587 | APAPPAQAPR |
| GLEAN3_17587 | APAPQAPAR |
| GLEAN3_17587 | APAPSAPR |
| GLEAN3_17587 | APAPSVPR |
| GLEAN3_17587 | APAYVPR |
| GLEAN3_17588 | AAPGFAPPGFAPPAPR |
| GLEAN3_17588 | APAPGHSPGYPPAR |
| GLEAN3_17588 | APAPPMPR |
| GLEAN3_17588 | APGGGGGQIPAR |
| GLEAN3_17588 | APGVAQAAPR |
| GLEAN3_17588 | APPPAAAPGSFAPSPGYSAPAGGR |
| GLEAN3_17588 | APSGPYVPNTPR |
| GLEAN3_17588 | APSPPSGAAGASRPR |
| GLEAN3_17588 | APSVPAVPRPR |
| GLEAN3_17588 | APTPGHPGYQPPR |
| GLEAN3_17588 | FVPVVVFPSK |
| GLEAN3_17588 | LYNDKCDLEEEDAYTQQNIR |
| GLEAN3_17588 | RPSAPSVPYAPSGGR |
| GLEAN3_17589 | APAPPTAPGAPYAPSAPLPPQTPYAPPAPR |
| GLEAN3_17589 | APQTPYAPQAPR |
| GLEAN3_17589 | APTFGSPALPR |
| GLEAN3_17589 | APVVAPPLIPR |
| GLEAN3_17589 | APYAPPVPAAPR |
| GLEAN3_17589 | SGVSISVNHK |
| GLEAN3_17589 | TTFQVPR |
| GLEAN3_17589 | TYHNECDVEDDSSR |
| GLEAN3_17589 | VPFPAPR |
| GLEAN3_17589 | VPQVPQTPYTPQAPR |
| GLEAN3_17590 | APAPPAPPAAPAAPAAPAAPAPK |
| GLEAN3_17590 | APAPQAPVVPYAPAVPR |
| GLEAN3_17590 | APGLPFAPQAR |
| GLEAN3_17590 | APSAPVRPYAPAPQAPSAPVRPYAPAPQAPSGPVGK |
| GLEAN3_17590 | APSPAAPSAPR |
| GLEAN3_17590 | APVQVPR |
| GLEAN3_17590 | QGETGYQAPVAPR |
| GLEAN3_17605 | AVDSLVPIGR (ATP synthase) |
| GLEAN3_17605 | VVDALGTPIDGK |
| GLEAN3_17737 | LISEGGSLCTK |
| GLEAN3_17737 | QCPDALDANIAE |
| GLEAN3_18054 | MEEDPDNMVAFDK |
| GLEAN3_18348 | TFQANANSDDVIK |
| GLEAN3_18348 | VVAVATQGR |
| GLEAN3_18348 | YNSNEFVK |
| GLEAN3_18406 | FPNIGTGGYPGSVFPHGPGYPR |
| GLEAN3_18406 | GGGYGGAGGR |
| GLEAN3_18406 | GIHPAAGGPAYNGR |
| GLEAN3_18406 | GVGGAGGAGGGTGAAGR |
| GLEAN3_18406 | HIPGYIPGR |
| GLEAN3_18406 | RPSASDSGSGGTGVNGGTGGGAR |
| GLEAN3_18406 | RTGVGILPDIQVIDPR |
| GLEAN3_18406 | STYPGQNYPGSR |
| GLEAN3_18406 | TGVGILPDIQVIDPR |
| GLEAN3_18406 | YPGYNPR |
| GLEAN3_18406 | YPNVGNPGMNYPGGYPGVGVGGFPGQGGYPGNNYPGQNYPGNNFPGSR |
| *GLEAN3_18452* | *EVAVEYTSGDAVYSR* |
| GLEAN3_18702 | DLPVIAIDSFR |
| GLEAN3_18702 | GNMNFLTADGTK |
| GLEAN3_18810 | ELGTIPGR |
| GLEAN3_18810 | LVASFSQDNQMER |
| GLEAN3_18810 | NPFGMPPGFAPVMR |
| GLEAN3_18810 | SPQENMEIYR |
| GLEAN3_18811 | AFVCEVPAGR |
| GLEAN3_18811 | MASEFCEMVTPCGNGPAK |
| GLEAN3_18811 | NIPIGQQPGMGQGGFGNQQPGMGGR |
| GLEAN3_18811 | QIPQGVGPQWEAVEVTAMR |
| GLEAN3_18811 | QPGFGNQPGMGGQQPGMGGQQPGWGNQPGVGGR |
| GLEAN3_18811 | QPGFGNQPGMGGR |
| GLEAN3_18811 | QPGFGNQPGVGGR |
| GLEAN3_18811 | QPGMGGQPGVGGR |
| GLEAN3_18811 | QPGMGGQQPGMGGQPGVGGR |
| GLEAN3_18811 | QPGMGGQQPGWGNQPGVGGR |
| GLEAN3_18811 | QPGMGGQQPNNPNNPNPNNPNNPNNPNPR |
| GLEAN3_18811 | QPGVGGR |
| GLEAN3_18811 | QPGWGNQPGVGGR |
| GLEAN3_18811 | SWPVNPQNPMSGPPGR |
| GLEAN3_18813 | CMTLPGQMQMNGAVQK |
| GLEAN3_18813 | GGWGQGGQGQGGQGGR |
| GLEAN3_18813 | TFTGCDGISPGHLAAPTTFEER |
| GLEAN3_18813 | TYDMASQSCK |
| GLEAN3_18813 | VENSCYR |
| GLEAN3_18813 | WNPNQGAGAGAGAGGR |
| GLEAN3_18813 | WNPQTPQNPGQGGR |
| GLEAN3_18919 | EISSGEGEQPK |
| GLEAN3_18919 | EVSSGQVEELK |
| GLEAN3_18964 | KLQIGIK |
| GLEAN3_18964 | LEDGTEFDSSIPR |
| GLEAN3_18964 | LVIPSNLGYGDR |
| GLEAN3_19665 | IDATSQLR |
| GLEAN3_19691 | AAAQQFLGK |
| GLEAN3_19691 | IAAEIAAPLSK |
| GLEAN3_19691 | IDTDNVYTR |
| GLEAN3_19691 | ISLNTMTLR |
| GLEAN3_19691 | SQQIQVQEQEIAR |
| GLEAN3_20322 | GVVDSEDLPLNISR (HSP90-β) |
| GLEAN3_20457 | FTCVATNPALQQPSTCSLTPLR |
| GLEAN3_20457 | LNPYGQVGEEQLSR |
| GLEAN3_20612 | DPVVASPPR |
| GLEAN3_20612 | TGTDTASDTASSSSSSSSSHSVSTTGSK |
| GLEAN3_20612 | YPAADPNDGTSTSK |
| GLEAN3_21260 | DVLSYPR |
| GLEAN3_21260 | IVISDPGNGLVVSMNLDGSLPK |
| GLEAN3_21260 | VISSLSSNYK |
| GLEAN3_21260 | VLAATLGAYR |
| GLEAN3_21385 | CGFLVAWITR |
| GLEAN3_21385 | FIQVVDYSR |
| GLEAN3_21385 | ITDPGTVHVWDMYR |
| GLEAN3_21385 | TTAPAADIAR |
| GLEAN3_21385 | YDQGAANSVAFDPASSFAYVAGNK |
| GLEAN3_21511 | EAFSLFDKDGDGTITTK (calmodulin-2) |
| GLEAN3_21511 | VFDKDGNGYISAAELR (calmodulin-2) |
| *GLEAN3_21630* | *GASFAGLGR* |
| GLEAN3_21991 | IAIGTSYEGR |
| GLEAN3_21991 | SVQALEGVHGTR |
| *GLEAN3_22047* | *VDCAESLCR* |
| GLEAN3_22057 | IVDQVSGIAR |
| GLEAN3_22057 | IVVDTVQGR |
| GLEAN3_22057 | NALYVGLPEAIVK |
| GLEAN3_22278 | APLPAPPAPR |
| GLEAN3_22278 | EEGNTRPNASLK |
| GLEAN3_22278 | KVNLFNE |
| GLEAN3_22278 | QGDTPEAPR |
| GLEAN3_23016 | EVFVSILDIDDETPR |
| GLEAN3_23016 | FDITDGTNPLIDR |
| GLEAN3_23016 | FTDMDSNDR |
| GLEAN3_23016 | GGMPISAFSIK |
| GLEAN3_23016 | GHLESTDNPGMPINSFTQLDLAGSK |
| GLEAN3_23016 | GTTVIDNTMLDAIDPDTDR |
| GLEAN3_23016 | GVEPEEDQFTFR |
| GLEAN3_23016 | IIDQPLLR |
| GLEAN3_23016 | ISFTDIDNK |
| GLEAN3_23016 | ISFTDIDNKEPVVR |
| GLEAN3_23016 | LITPFELAIDDR |
| GLEAN3_23016 | LITVDEGGQR |
| GLEAN3_23016 | LVPSNFPCDFGPGEVK |
| GLEAN3_23016 | NQPITSFTQK |
| GLEAN3_23016 | NTGISVPFGR |
| GLEAN3_23016 | QEYFQSMVR |
| GLEAN3_23016 | QSEPLSQIPGR |
| GLEAN3_23016 | VSYRPPSTELGIAPR |
| GLEAN3_23016 | VTVLSSSSGLPR |
| GLEAN3_23016 | VVDSSDPQPNESPVQEFLVK |
| GLEAN3_23052 | CYQCFGLGSR |
| GLEAN3_23052 | DFSQLPNIDEVR |
| GLEAN3_23052 | HPIASCTDIGPQSQTPTIDCTGK |
| GLEAN3_23052 | NQLLAQTASK |
| GLEAN3_23115 | AAAPGQVMVVDVMER |
| GLEAN3_23115 | HGPEVIYHYR |
| *GLEAN3_23217* | *FNVWDTAGQEK (RAN)* |
| GLEAN3_23855 | YASYLNFGNEK |
| GLEAN3_24083 | LGIAPQIQDLYGK |
| GLEAN3_24188 | ALIALAS |
| GLEAN3_24188 | GLGTDEGCLGR |
| GLEAN3_24564 | FTYQIVSR |
| GLEAN3_24564 | IDEYVLPK |
| GLEAN3_24565 | TSDILSDTTK |
| GLEAN3_24800 | AIAAAAAGDISK |
| GLEAN3_25068 | GAIIMELESK |
| GLEAN3_25068 | YNTPGEGGLTK |
| GLEAN3_25235 | DPGQVTEGVR |
| GLEAN3_25235 | DTCAQNPCFEGVR |
| GLEAN3_25235 | GICQPICER |
| GLEAN3_25235 | LTCVDDNECQSNR |
| GLEAN3_25235 | TGTIVTFVK |
| GLEAN3_25235 | TVDVSNPQDVR |
| GLEAN3_25235 | VSETVNYACK |
| GLEAN3_25502 | DAILYTPR |
| GLEAN3_25722 | QPITISR |
| GLEAN3_25722 | LTELFPK |
| GLEAN3_25502 | VSGTLQINNVR |
| GLEAN3_25772 | NEVEDAVVR |
| GLEAN3_25772 | VDATVETQLAGK |
| GLEAN3_25966 | ADAMPENAALK |
| GLEAN3_25966 | CAFPAITTLPK |
| GLEAN3_25966 | ELFLQDNLINSVSR |
| GLEAN3_25966 | GAFSSMTQLQTLR |
| GLEAN3_25966 | IDTSELVCGTPFGVR |
| GLEAN3_25966 | KYGGSLCQILKPGNPTK |
| GLEAN3_25966 | QQATYDPIGHLK |
| GLEAN3_25966 | SIPIHPDVNLFSMGK |
| GLEAN3_25966 | SPQQTYSLGPGAYR |
| GLEAN3_25966 | TLQLADNPLER |
| GLEAN3_25966 | VDGAMFSR |
| GLEAN3_25966 | VFYSLVGDR |
| GLEAN3_25966 | YGGSLCQILKPGNPTK |
| GLEAN3_26000 | CIVILPSGVK |
| GLEAN3_26000 | FLVSADR |
| GLEAN3_26000 | FSIAGDSNLGEHDLMIR |
| GLEAN3_26000 | LIGESTLK |
| GLEAN3_26000 | NQPDTVTVYAGDTIYMR |
| GLEAN3_26000 | SDNTNPQTQYISIR |
| GLEAN3_26000 | SEFTVIR |
| GLEAN3_26000 | VLPADTQPAAVNPR |
| GLEAN3_26000 | VPIPIPTCSMTPPQPSVGQR |
| GLEAN3_26000 | YSDGSIFFTR |
| GLEAN3_26042 | CSVGCGTGTQTR |
| GLEAN3_26042 | LCSYSYYR |
| GLEAN3_26094 | AIAYYTESISK |
| GLEAN3_26094 | ISGLDPAGPGFGGK |
| GLEAN3_26146 | DSYNTVFANFEK |
| *GLEAN3_26306* | *IDDVAIENYR* |
| *GLEAN3_26630* | *SAYGGSSQTYFR* |
| GLEAN3_26949 | ATDSTLTLETLK |
| GLEAN3_26949 | YAGIAVVR |
| GLEAN3_27169 | MQAPAFGLASTTFK |
| GLEAN3_27172 | AVAVDLPGYGESK |
| GLEAN3_27172 | AVVEGQAETTQVCK |
| GLEAN3_27172 | DESIGLESLK |
| GLEAN3_27172 | EVLPATEPK |
| GLEAN3_27172 | GFVPVAPVGTNK |
| GLEAN3_27236 | AVPSSYSDLGK |
| GLEAN3_27236 | ITLSTLIDGK |
| GLEAN3_27236 | LTLDTSFSPQTGK |
| GLEAN3_27236 | SNFALGYK |
| GLEAN3_27236 | YVLDSEASLNAK |
| GLEAN3_27906 | LLDIADFDSFR |
| GLEAN3_27906 | NLYTHTLPFYQPPTGQQLDFIPPEK |
| GLEAN3_27906 | NPGNPNQPVR |
| GLEAN3_28091 | LEAVLTQGR |
| GLEAN3_28091 | TPIVNFFDR |
| GLEAN3_28135 | ADTDAAPAAPAPSTPK |
| GLEAN3_28135 | GVTSGQLR |
| GLEAN3_28221 | AVMVDLEPSVVDEVR |
| GLEAN3_28221 | DVNAAIATIK (α-tubulin) |
| GLEAN3_28221 | EHIDNVMDR |
| GLEAN3_28221 | LIGQIVSSITASLR (α-tubulin) |
| GLEAN3_28221 | SIQFVDWCPTGFK (α-tubulin) |
| GLEAN3_28748 | TYDAITLIR |
| GLEAN3_28748 | VDEGYPTNIK |
| GLEAN3_28749 | GAPSNIDAIFEKPGGTTVMIK |
| GLEAN3_28749 | NSQLVQGYPVR |
| GLEAN3_28749 | NVLEDIPGLPLGIDAAFSSK |
| GLEAN3_28749 | QRVDNNEYPR |
| GLEAN3_28749 | TASYFVR |
| GLEAN3_28749 | TTITFTFDNYTPDLPMNQVR |
| GLEAN3_28749 | VDNNEYPR |
| GLEAN3_28749 | VWSDVTPLK |
| GLEAN3_28749 | YDHSSGSLSQGFPR |
| *GLEAN3_28880* | *APAPLAVSPLIR* |
| *GLEAN3_28887* | *SFQDADAGISAEDR* |
|  |  |

Tentative identifications are shown in italics. Peptides shared with human proteins are shaded yellow. Peptides are arranged according to increasing Glean3 entry number.
