# Supplementary material for: In-depth, high-accuracy proteomics of sea urchin tooth organic matrix
Source: Proteome Sci. 2008 Dec 9;6:33. doi: 10.1186/1477-5956-6-33 (PMC2614417; doi:10.1186/1477-5956-6-33)
Supplement: Additional file 7 — Proteins with Ala- and Pro-rich and acidic Gly-rich motifs. Identification of motifs in proteins with Ala and Pro-rich and acidic Gly-rich sequences contained in entries Glean3:17590, Glean3:17587 and Glean3:17588. [file 1477-5956-6-33-S7.doc]

**Proteins with Ala and Pro-rich and acidic Gly-rich motifs.**

GLEAN3_17590/22278

17590 **1** ***MAVFFWMSFF LVQFPNMDHT NILTALGIVL LLAVLSNG***AP YSNKCTRGVS

22278 **1** ***MNHT NVLAALGIVL YLAVLSKG***AP YLNKCTRGVS

17590 **51** GSVCGDNGVT YDDACDVDDD DDDTNGNGGV QVAHSGPCGG GSQSPAAPR**A**

22278 **35** GTVCGDNGIT YGDACDADDD DDDTNGNGGV QVAHSGPCGG GSQSPAAPR**A**

17590 **101 PAPQAPVVPY APAVPR**APPA SAPSAPYAPA APYAPPASAP SAPYAPAAPY

22278 **85** **PAPQAPVVPY APAVPR**APPA SAPSAPYAPA APYAPPASAP SVPYAPAAPY

17590 **151** APPASAPSAP HAPAAPYAPG GGANGDTSGE TGYEQSPSVG YAPAAPAVGY

22278 **135** APVA------------PYAPG GGANGDTSGE TGYGQSPSVG YAPAAPAVGY

17590 **201** APAAPAVGYA PAAPAVGYAP SVGYAPAVGY APAGTDVGYA PASGDSGYDY

22278 **174** APAAPAVGYA PAAPAVGYAP ---AAPAVGY APAGTDVGYA PASGDSGYDY

17590 **251** VYEDDSDSSE EGYGYEYYGY EAIPAGPVGF APAPAAPAFA PSGSGYAPGG

22278 **221** VYEDDSDSSE EGYGYEYYGN EAVPAGPVGF APA------- ------APGG

17590 **301** QAYAPVGGGA NGCGGSGSGE GCDGGIDTGA AGPIGFAPAP AAPAFAPSGQ

22278 **258** TAYAPGAGGT GGCGGTDSGE GCDG-----A AGPIGFAPVP AGPPFAPSSP

17590 **351** GYAPAAPAYA PVQEGGAGAC EDSGSGEGCD GDESDDEDSG SEEGSDSVAT

22278 **302** GYAPGGPSYA PVPEVSADGC DDSGSGEGCD GDESDGEDSG SVEGSDSAAP

17590 **401** GGVAAPGGAA APAPGAPR**AP GLPFAPQARA PVQVPRAPSA PVRPYAPAPQ**

22278 **352** GGAVAPGGAA APAPGAPPAP GLPYAPPVR**A PLPAP----- ------PAPR**

17590 **451 APSAPVRPYA PAPQAPSGPV GK**DDSSEK**QG ETGYQAPVAP RAPSPAAPSA**

22278 **392** APSAPVRPYA PAPA-PQAPS GPSDSSEK**QG DTPE----AP RAPSPAAPSA**

17590 **501 PRAPAPPAPP AAPAAPAAPA APAPK**APSGG CTAGDAGCNG GSGGGGDNGA

22278 **437** **PRAPAPPAPP AAPAAPAAPA APAPK**APSGG CTVGDVGCSD ---GGGDNGG

17590 **551** NDCNDSG------------------------- -CKDDDDDNG QNIRRLNSNG

22278 **484** EECTESGYSN KCTRGVSGIV CGDNGVTYDD ACDVDDDDDD TNGNGGVQVA

17590 **577** I

22278 **534** HSGSCTRGSH SPAAPRAPAP HAPAAPYAPI APYAPHAPAG GVHAGTSGET

22278 **584** GYGHAPSPGV VGYAPPPYAP GAPYAPHAPA AGVPSGTSGE TGYAPEGPAV

22278 **634** GYAPGGRAPY APGAPYAPNA PAAGVHSGTS GETGYAHVGP AVGYAPGGPA

22278 **684** PYAPGAPYAP YAPAAGVHSD TSGETGYGPV GPAVGYAPAA SGVGYAPEEG

22278 **734** ALGYDFDGDD SDSGEDYHGQ EAAPGAPVGY APAAPVGYAP AAAGGYAPAT

22278 **784** PVGYAPAAPG GYAPSDTGLG CGGSDSEEGG DDSSDEDGCG LAAPPGGGAP

22278 **834** GAAAPAPGYP HAPPAPRAPY APHAPIPGAP SYAPGAPFAP YAPGPNHDAS

22278 **884** SAMDEEEQND QGDEEDNEDE EDQDQEDKDE EDQDEEDKDE EDQDKEDKDE

22278 **934** EDKDEEDKDK EDKDAEEEEE EDNADEEEDA DKDEEDKKDE EDNGDKEDNS

22278 **984** DNEEGCSGAD CAK**EEGNTRP NASLKKVNLF NE**

GLEAN3_17587

**1** ***MNFKTAVALL ALLAVVADLS AA***APREKRDD NGNGCDNGGS DCGSSDPCSA

**51** DPGACAPAAP APAPGPAPR**A PAPPAPVPRA PAPPAQAPR**A PRPRAPAAPR

**101** APMRAPSRAP AAPRAPMRAP ARAPAAPAFA PSAPGEYAAY GGAEGADGAD

**151** GGDYEYGYDY AYGGDSSGEG YGGGCGDSSE ECGGYGDGAG GASSGGGGGG

**201** ASGGGAGGAD GGDGGDYSAA VGGGAAAAPG GYPAAAPGGY PAAAPSRYPA

**251** AAAPRPRAPA R**APAPQAPAR** APAPQAPAR**A PAYVPR**APVY R**APAPSVPRA**

**301 PAPSAPR**APA QRPVPRPAAP APIPAPAPRA PVAPPAPAPP APPAPPAPPA

**351** PAPAPEAPSA PEPESPSDGD NGDNGCGDNG DGGTSNGCDT TTGDYVTIFK

**401** DMYLNRRSYI PASLSRYPTY LFP

GLEAN3_17588

**1** ***MMKTFTGLAL LTCFFLLCRE EEVEAR***VMPE PTDVVMEAVT VVEMADAAEA

**51** GAEEVMEEVT EAVTVVEMAD AAEAGAEEVM AVLAEVATEV VTEDVVVAME

**101** VVTEAVVVET QTEGVQREVL VAVVVVAAAA PAVRRALKAS LEVRRDVKVV

**151** REEKEVKEEE KDAVVKEETV GVVKVVAKVV MVVEMLVVVV EMLVALQVPG

**201** EGTGHMGGST RKKSGNAGSR PRGRSRSEKK NNHSVKESAN KVSSDSHRPI

**251** LHFVPK**FVPV VVFPSK**SPSG GTTLQGGGGG EKRVAGGSED SGKGVAGGSE

**301** DSGKGVAGGS EDGGKGVAGG SEDVGKGVAG DSEDGGDGGD GADGGDRGDK

**351** GGVGGGEGVC EEECDTTDAF IFKRDDNGGT GAECGSTGSC GADPTPCSPA

**401** TPAPAPKPQA PAPPVPAPRA PVSYPKATAP SPRAPRPRAP ARAPKARAPP

**451** ARDLPAFAPS APASGPARDA VDGVEGTADT ADYDGGYGGG SIGEGYGGCG

**501** SDSSEECDSS SPGGVEQSQG GNGGEVAEGG IDFHVVSGSK PAAAPAGSPA

**551** QSPRHRVANA IPRAPAPPAP RAPARRPKIP SPPPRRPASS PAVPKAPQPK

**601** APAPPKPSAP MAPAPEPKAP APPKPSAPEP EAPSGDKGCD ENGENGGSSN

**651** GCDTTTGDNG KTKTMNILRS AVILMLVFSA SLAAPRSPRD SCDRGVSGPV

**701** CGSNGK**LYND KCDLEEEDAY TQQNIR**VTNY EYCRSSGRAP QAPSRPGYNP

**751** PARAPAPYPP ARGPVPRPAP RAPAAPPRAP GFAPAPNAPS PHNPDQRYAG

**801** GSSGSEHNIP GYAPSPGAPG APGYAPPPPA PRAPGYAPLP PRR**AAPGFAP**

**851 PGFAPPAPR**Q FAPSAPGVGY APSAPAAGAF GVFADGGDGA GGEGAEASTD

**901** VSGSDGDDSE SSEEGYDYDG SDGIDGEEET EGADGVNGAD AGTGTTGGVS

**951** GAPSGPGVAG APNGPGVFQR GPNVPGVSRP GGPPR**APSGP YVPNTPR**RPY

**1001** TPTFGRAAPA LPR**APGVAQA APR**APGGSYG TTGCTQNAPG NTCTSYGAND

**1051** NVPRDSCDGS DTRNVCGSNG KSYNNECELE EEASQTGQDV SVSHYGSCQS

**1101** SSGGTQSAPY *APGGGGGQIP AR****APGGGGGQ IPAR****APGGGG GQIPAR***APSV**

**1151 PAVPRPR**APA QGPVAPAPPA HAPIPGPAVP GYQPGAAAPS APQYDQAGGS

**1201** SGSHDSTSQY APAPAAPSAP GYAPSPAAPT RPASAPRPAA PRPAAPRPAA

**1251** PRPAAPRPGP AAAPRPAGPS FAPSAPIPGV GFAPGAPATG GFVAGDAAGA

**1301** AAGAAGAAGD GAAGAAGAGS GSGEGDGSDS SEEAYDYDGS DGAEGADGTE

**1351** GADGADGVEG VDGADGAGEG GGTSAAGVPG TASGVPSTRG GVGVARAPGG

**1401** ARAPGGARAP SRPR**APSPPS GAAGASRPRA PPPAAAPGSF APSPGYSAPA**

**1451 GGRRPSAPSV PYAPSGGR**AL PRAPGGTPGG APGYAPAGAP GYAPVGAPVA

**1501** PGVATGTGTC SEGVSADYGC TEEALYDPNN SGDQDTRDSC DLGTPGAACG

**1551** SDGQSWGAGG TPAR**APAPGH SPGYPPARAP TPGHPGYQPP R**APAPGPYPA

**1601** APHAPGPQQP YAPIAPANAP GVPYAPGNPP NAPPPYYPQA PQAPPPYQPN

**1651** APSPPSYAPN APSMPGFAPG PPGAGVPFGP DGNDAGGSSD QQSADPAPAP

**1701** R**APAPPMPR**A PTFVPAAPRP AAPRPPVYAP AAPRPAAPAP AAPAAPAVPG

**1751** TRFAPAAPGE GGDGGDGVDV GEYDGEAGAD YGHYESEGSS GSDESSGSSE

**1801** EYYDYGSGGI DGGDGADGAD GGDGAGGAGG GDGGDGADGV GGVGAGTGAA

**1851** GGRGGLPRAP SRPGTPRAQG GPRAPGSPRA PGGPRAPGNP R**APGIPR**APG

**1901** IPRAPTRPGV PVRPYAPGPR QSAPRAPGGP GAPGAPGAPG APGVPGAPGA

**1951** PGGGPAHAPS GGSCNNNGPS GQACSGGVSG KR

|  |
| --- |

Predicted Kazal domains, Ala/Pro-rich, acidic and acidic Val-rich motifs are shaded as indicated. Predicted signal peptides printed in ***bold italics***. Peptides sequenced by MS/MS are in red. Identical sequences in the alignment of the sequences of entries Glean3:17590 and Glean3:22278 are underlined. DEED and similar repeats in the C-terminal acidic domain of entry Glean3:22278 are underlined.
